# Exploring the Influence of Spacers in EDTA–β-Cyclodextrin Dendrimers: Physicochemical Properties and In Vitro Biological Behavior

**DOI:** 10.3390/ijms241914422

**Published:** 2023-09-22

**Authors:** Israel González-Méndez, Kendra Sorroza-Martínez, Ignacio González-Sánchez, Jesús Gracia-Mora, María Josefa Bernad-Bernad, Marco Cerbón, Ernesto Rivera, Anatoly K. Yatsimirsky

**Affiliations:** 1 Departamento de Química Inorgánica y Nuclear, Facultad de Química, Universidad Nacional Autónoma de México, Circuito Escolar, Ciudad Universitaria, Mexico City C.P. 04510, Mexico; israel.mendez@uaem.mx (I.G.-M.); jgracia@unam.mx (J.G.-M.); 2 Centro de Investigaciones Químicas-IICBA, Universidad Autónoma del Estado de Morelos, Av. Universidad 1001, Col. Chamilpa, Cuernavaca C.P. 62209, Mexico; 3 Instituto de Investigaciones en Materiales, Universidad Nacional Autónoma de México, Circuito Exterior, Ciudad Universitaria, Mexico City C.P. 04510, Mexico; kendraivn17@gmail.com; 4 Departamento de Biología, Facultad de Química, Universidad Nacional Autónoma de México, Circuito Escolar, Ciudad Universitaria, Mexico City C.P. 04510, Mexico; ignacio.gonzalez.s@gmail.com (I.G.-S.); mcerbon85@yahoo.com.mx (M.C.); 5 Departamento de Farmacia, Facultad de Química, Universidad Nacional Autónoma de México, Circuito Escolar, Ciudad Universitaria, Mexico City C.P. 04510, Mexico; bernadf@quimica.unam.mx

**Keywords:** EDTA dendrimer, β-cyclodextrin, CuAAC, supramolecular chemistry, self-assembly

## Abstract

The synthesis of a new family of ethylenediaminetetraacetic acid (EDTA) core dimers and G0 dendrimers end-capped with two and four β-cyclodextrin (βCD) moieties was performed by click-chemistry conjugation, varying the spacers attached to the core. The structure analyses were achieved in DMSO-*d*_6_ and the self-inclusion process was studied in D_2_O by ^1^H-NMR spectroscopy for all platforms. It was demonstrated that the interaction with adamantane carboxylic acid (AdCOOH) results in a guest-induced shift of the self-inclusion effect, demonstrating the full host ability of the βCD units in these new platforms without any influence of the spacer. The results of the quantitative size and water solubility measurements demonstrated the equivalence between the novel EDTA-βCD platforms and the classical PAMAM-βCD dendrimer. Finally, we determined the toxicity for all EDTA-βCD platforms in four different cell lines: two human breast cancer cells (MCF-7 and MDA-MB-231), human cervical adenocarcinoma cancer cells (HeLa), and human lung adenocarcinoma cells (SK-LU-1). The new EDTA-βCD carriers did not present any cytotoxicity in the tested cell lines, which showed that these new classes of platforms are promising candidates for drug delivery.

## 1. Introduction

The field of pharmaceutical nanotechnology has been of great interest for researchers because it can improve the performance of existing therapies and can reduce the high costs of designing and manufacturing new drugs. Dendrimers are among the nanostructure-based drug delivery systems that have been designed and evaluated for the sustained and controlled delivery of diverse drug molecules [1,2]. Dendrimers are three-dimensional structures bearing a central core connected by successive layers of branching and terminal functional groups. They have unique properties such as nanometric size, monodispersity, high loading capacity, adjustable solubility, biocompatibility, controllable surface functionality, and high loading capacity [3,4]. In recent years, there has been a particular trend in the design and study of nitrogen-containing dendrimers, since their chemical environment resembles that of proteins, making them important candidates for applications in drug delivery [5]. In this regard, ethylenediaminetetraacetic acid (EDTA) is a potential molecule that has been used as new core for the preparation of dendrimers. EDTA has tertiary amines and is tetra-directional, which offers an adequate number of branching sites [6].

The terminal functional groups present in the periphery of the dendrimeric scaffold are the most important factor, since they control the macroscopic physicochemical properties and the behavior in biological media [7,8]. Therefore, the chemical modification of the dendrimer surface is a feasible approach to design and control their biological interactions in order to obtain a new class of dendrimers with improved properties [9,10]. Molecules of different types can be conjugated to the periphery of dendrimers [11,12], such as lipids, amino acids, proteins and peptides, polymers, and carbohydrates. Specifically, the conjugation with carbohydrates to the periphery of dendrimers offers improved properties in their biological behavior, such as lower hemolytic toxicity, reduced immunogenicity, and targeted delivery [13,14]. In this way, modifying the surface of the dendrimers, for example, with β-cyclodextrin (βCD) molecules, allows the creation of a synergy in the properties of both components, such as biocompatibility and solubility [15]. βCD is hydrophilic entity possessing a hydrophobic conical cavity. Besides this, it is also known to form inclusion complexes in the solid state and in aqueous solution with a large variety of organic targets of hydrophobic nature and suitable size and geometry, and is involved in many supramolecular applications [16,17].

Dendrimer chemistry has been constantly updated to offer advanced macromolecules with better structural and functional control. In this regard, the concept of “click chemistry”, using the Cu(I) catalyzed azide alkyne cycloaddition reaction (CuAAC) to produce 1,4-disubstituted 1,2,3-triazoles, has been further utilized to engineer new dendrimer structures as well as for the conjugation of bioactive molecules to dendrimers [18,19]. So, in this work, we present the design, synthesis, characterization by NMR (^1^H and ^13^C), IR (ATR), MS (ESI or MALDI), and the systematic evaluation of a new family of dimers and dendrimers bearing EDTA as a core, functionalized by CuAAC with two and four βCD moieties in the periphery, varying the spacers between the core and the aromatic rings through accessible synthons. Our aim with these new designs is to prove that our molecules have similar properties to PAMAM dendrimers (e.g., size, water solubility) regardless of the spacer used and, according to our synthetic route, with the advantage of their rapid obtention. Moreover, during the synthesis of the final molecules presented here, the CuAAC classical condition was improved to obtain the complete functionalization with high yields.

On the other hand, it has been demonstrated that dimers and tetramers based on βCD are able to adopt unusual conformations and behave as pseudo[1]rotaxanes in aqueous solution through self-inclusion in one of the pyranose units of βCDs [20,21,22]. This inversion phenomenon results in limited accessibility to the βCD cavities, which is of major importance for understanding and controlling systems containing these units [23]. For this reason, in this work, we compare the adopted conformation in three dimers of type EDTA di-βCD and three EDTA G0-βCD dendrimers in DMSO-*d*_6_ and D_2_O. Furthermore, the formation of the inclusion complex (IC) between the compounds and 1-adamantanecarboxylic acid (AdCOOH) is investigated in order to demonstrate that it was entirely possible to reverse the self-inclusion process with the AdCOOH guest for all of the EDTA-βCD platforms. Lastly, we determine the in vitro cellular behavior for these molecules in four different cell lines: two human breast cancer cell lines (MCF-7 and MDA-MB-231), human cervical adenocarcinoma cancer cells (HeLa), and human lung adenocarcinoma cells (SK-LU-1) as a part of the rational evaluation, since our perspective is the potential use of these platforms as potential carriers of anticancer drugs such as camptothecin, tamoxifen, resveratrol, or doxorubicin.

## 2. Results and Discussion

### 2.1. Synthesis

**Scheme 1:**
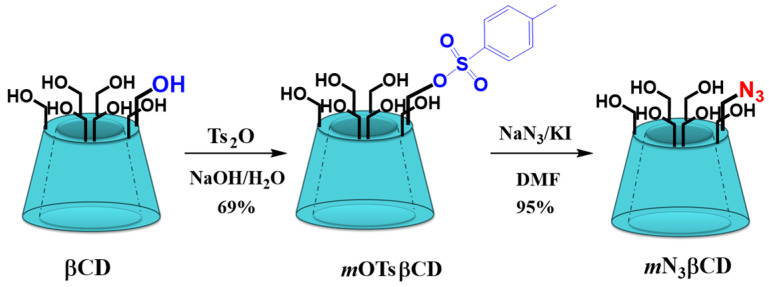
Synthetic route for the obtention of *m*N_3_βCD.

A key step to obtain the proposed compounds lies on the mono-functionalization with one azide group at the primary face of βCD. This was performed in two steps from βCD by following standard literature procedures [24,25,26] to afford the 6-*O*-monoazido-β-cyclodextrin (*m*N_3_βCD) in a quantitative yield (95%), as shown in Scheme 1.

**Scheme 2:**
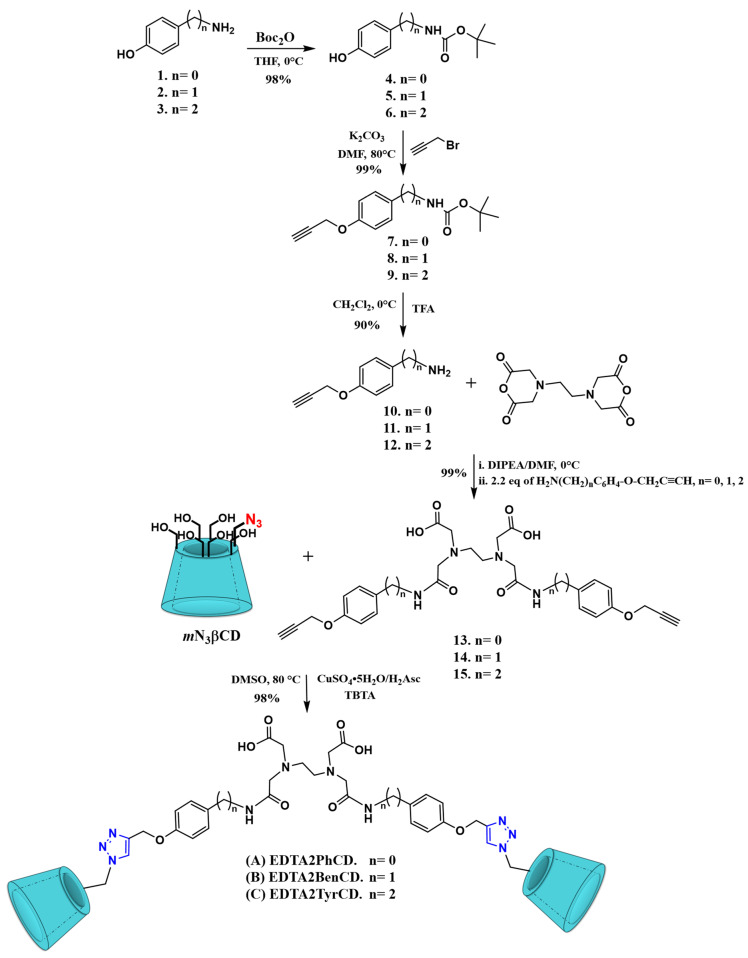
Synthetic route of dimers EDTA-βCD.

For the selection of the spacers presented in the EDTA-βCD molecules (**A**–**F**), we looked for easily accessible synthons that did not imply an additional step of synthesis; thus, our design started preparing analogs of the PAMAM G0 dendrimers that can be easily synthesized and scaled. In this way, we selected 4-aminophenol (Ph) (**1**), 4-hydroxybenzylamine (Ben) (**2**), and tyramine (Tyr) (**3**) as starting materials and we took their acronyms to identify the final compounds, naming them according to the compound from which they came, as shown in Scheme 2. The employed synthesis strategy was based on the objective of functionalizing the EDTA core with the appropriate derivative in such a way that the spacer grew from the core to the periphery (divergent synthesis), maintaining for each of them the *para*-substituted aromatic ring with a propargyl ether terminal as a source of alkyne groups. So, the corresponding aromatic (**1**) or aliphatic amine (**2**–**3**) was selectively protected by the slow addition of di-*tert*-butyl dicarbonate (Boc_2_O) under low-temperature conditions that allowed us to obtain the corresponding protected amines (**4**–**6**) with yields higher than 95%.

Afterwards, the intermediates **4**–**6** were reacted in the presence of propargyl bromide in basic conditions via a classical Williamson etherification to obtain terminal propargylated intermediates **7**–**9** with yields higher than 90% [27,28]. According to our previous experience in the *N*-Boc deprotection of other derivatives [29], the intermediate compounds **7**–**9** were treated with an equivalent dichloromethane:trifluoro acetic acid (CH_2_Cl_2_:TFA) mixture at low temperature [30]. To avoid exposing the products to a strongly acidic medium for an excessive time that could cause a decrease in yields, an acid–base extraction was performed once the deprotection reaction was completed in all cases. The deprotected compounds **10**–**12** in their –NH_3_^+^ forms were transferred into the aqueous phase. Then, the extraction of corresponding compounds **10**–**12** in the organic phase was carried out after adjusting the pH value to 12 in the aqueous layer, making possible to recover the desired compounds in the -NH_2_ form in an average 95% yield without the need for chromatography purification.

**Scheme 3:**
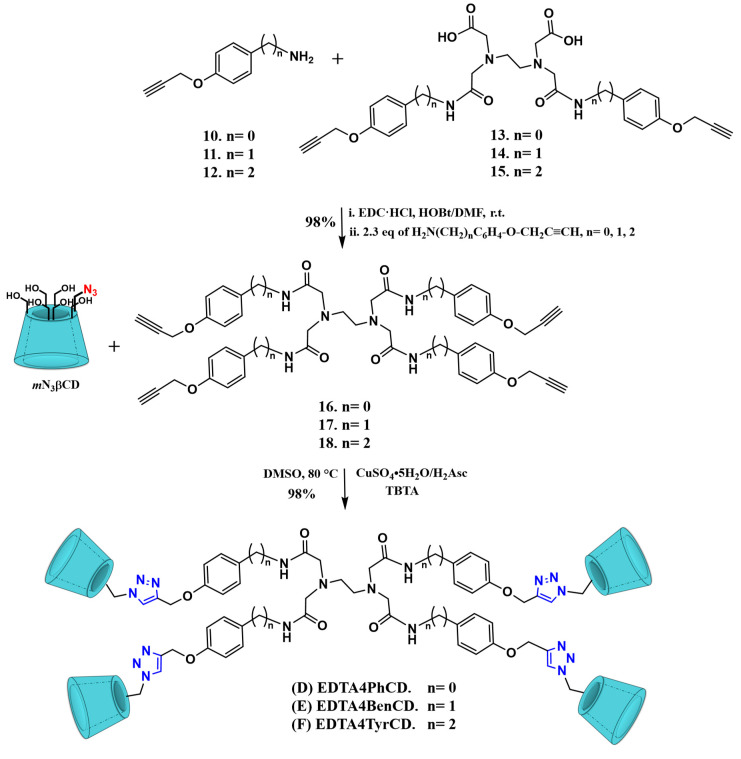
Synthetic route for dendrimers EDTA-βCD.

For the obtention of the disubstituted alkyne intermediates bearing an EDTA core (**13**–**15**), EDTA dianhydride was used as starting material and reacted through nucleophilic addition with the selected amine under basic conditions. For this reaction, 2.2 eq of amines **10**–**12** was required and a subsequent recrystallization from MeOH was necessary for obtaining a 99% yield in all cases, as shown in Scheme 2. On the other hand, the corresponding amide bond formation by condensation between carboxylic acids present in compounds **13**–**15** and the selected amine **10**–**12** was performed according to previous reports [29,31], using *N*-(3-dimethylaminopropyl)-*N*-ethylcarbodiimide hydrochloride (EDC·HCl) and 1-hydroxybenzotriazole hydrate (HOBt), to obtain the corresponding tetrasubstituted EDTA G0-alkynes **16**–**18** with 98% yield for all of the projected derivatives, as shown in Scheme 3.

To obtain the final projected EDTA di-βCD molecules (**A**–**C**) and EDTA G0-βCD dendrimers (**D**–**F**), CuAAC “click chemistry” was employed as a robust and versatile tool for the dendrimer construction [32,33,34]. According to the experience of our research group and in agreement with the synthesis methodologies previously described, we started with the classical reaction conditions employing a Cu(I) source generated in situ when the copper (II) in the salt (CuSO_4_•5H_2_O) was reduced in the presence of ascorbic acid (H_2_Asc), in a degassed dimethylsulfoxide:water (DMSO:H_2_O) solvent mixture in the proportion of 1:1. This reaction condition appeared to be optimal for the kind of alkynes obtained by us and allowed the obtention of the products with high yields. This reaction condition was found to be optimal for click reactions between the di-alkynes **13**–**15** or dendritic alkynes **16**–**18** with the *m*N_3_βCD; however, this was only true for the final compounds **C** and **F**. We believe that these results are due to the high rotational freedom of the ethylene spacer present in these alkynes, which allows all of the alkyne groups, two in intermediate **15** and four in dendrimer **18**, to carry out the Hüisgen [3 + 2] copper catalyzed cycloaddition, without any interference. Unfortunately, when trying to replicate these reaction conditions for the obtention of the rest of the final compounds, this was not possible since the reaction of the dialkyne **13** with 2.4 eq of *m*N_3_βCD did not result in the expected dimer formation, and only monosubstituted intermediates accompanied by unreacted substrates were identified. Moreover, the same phenomenon was observed for the corresponding tetra-alkyne **16** when it was reacted with 4.4 eq of *m*N_3_βCD.

This same behavior was observed by Chmurski et al. [23] for the preparation of other types of dimers. The explanation is that once the first triazole is formed by the click reaction, the remaining alkynes form intramolecular IC with the recently bounded neighboring βCD cavity, which causes the hiding of the remaining alkynes, thereby preventing the progress reaction for the formation of the triazole ring. To overcome this limitation, we decided to combine two strategies. Firstly, we completely removed the aqueous medium that promotes intramolecular IC formation and only used DMSO as a reaction medium. Secondly, a small change in the order of addition was very useful for us, since the independent preparation of the strongly chelated copper (I) source with TBTA as ligand and the subsequent addition of this freshly prepared mixture on top of the alkyne solution and the azide source allowed the completion of the reactions with high yields (>90%). Thus, this methodology was implemented for the synthesis of all of the final compounds **A**–**F**.

### 2.2. Characterization 

In order to demonstrate the obtention of the synthesized intermediates and final compounds, key signal changes were tracked by NMR spectroscopy using DMSO-*d*_6_ as a solvent, and the functional group modifications on each synthetic step were followed by IR spectroscopy. The molecular weights of the intermediates and final compounds were corroborated by mass spectrometry using DART, ESI, or MALDI techniques, depending on the largeness of molecular weight. 

Regarding intermediates **4**–**12**, since we used protection–deprotection methods and the corresponding addition of propargyl ether based on previous reports [35,36,37], the follow-up of the signals is in agreement with the literature in all cases, and the same was observed for the βCD *mono*-modified intermediates (see Figures S1–S36 in the Supplementary Materials).

In the ^1^H-NMR spectra of the final alkyne intermediaries **13**–**18**, the proton assigned to amide groups appeared between 10.10 and 7.96 ppm, and then aromatic protons of the *para*-substituted ring were observed at 7.56 and 6.87 ppm. The terminal alkyne proton signal appeared at 3.55–3.56 ppm in all of the molecules. The signals corresponding to the Ha protons of the EDTA core appeared centered at 2.53–2.87 ppm as singlet signals in the ^1^H-NMR spectrum (see Figures S37, S41, S45, S49, S53, and S59 in the Supplementary materials). The nonequivalent H-b,b′ protons appeared at 3.47–3.18 ppm for the intermediates **13**–**15** as two well-differentiated signals. The methylene protons used as spacers between the core and the aromatic rings were observed as a double signal centered at 4.23 ppm for compound **14**, while for compound **15**, two characteristic signals at 3.36 ppm, overlapping with H_2_O, were assigned to Hc, and the signal at 2.86 ppm arose from Hd (see Figures S41 and S45 in the Supplementary Materials). The ^1^H-NMR spectra of intermediates **16** and **18** showed a pattern of signals equivalent to that described for the dimers from which they came, except for compound **17** (see Figures S49, S53 and S59 in the Supplementary Materials), which exhibited a particular behavior, since the Hf signal of methylene belonging to the propargyl ether appeared to overlap as a multiple signal at 4.76 ppm with the Hc protons of the spacer, unlike the rest of the alkynes, where these signals are differentiable. This phenomenon was also observed for the Hb equivalent protons that appear to overlap with the Hg proton signal of the terminal alkynes; these assignments were corroborated with HMQC and COSY experiments (see Figures S55 and S56 in the Supplementary Materials). Furthermore, the structure of the intermediaries **13**–**18** was confirmed by 13C NMR (see Figures S38, S42, S46, S50, S54, S60 in the Supplementary Materials), IR (see Figures S39, S43, S47, S51, S57, S61 in the Supplementary Materials) and mass spectrometry using the ESI technique (see Figures S40, S44, S48, S52, S58, and S62 in the Supplementary Materials), where the molecular ions corresponded to the expected molecular weights.

The full characterization of the new EDTA di-βCD dimers (**A**–**C**) and EDTA-βCD dendrimers (**D**–**F**) was performed using NMR techniques in DMSO-*d*_6_ (^1^H-,^13^C-NMR and 2D NMR HMQC, COSY, IR, and MALDI-TOF (see Figures S63–S68, S72–S77, S80–S85, S88–S93, S96–S100, and S102–S106 in the Supplementary Materials). We also compared the behavior of all platforms in D_2_O. In previous work, we carried out a detailed analysis of similar platforms to those presented here, and according to our experience and in agreement with the previous work, we can affirm with certainty that in DMSO-*d*_6_, all of the platforms based on EDTA-βCD present a symmetrical conformation (Figure 1A) [31]. Thus, the signal analysis for all of the final molecules in DMSO-*d*_6_ showed a singlet at 8.16 ppm assigned to the classical proton in the 1,4-disubstituted triazole group, followed by four peaks in the aromatic region of the platforms (see Figure 1A, Figures S63, S72, S80, S88 and S102 in the Supplementary Materials), which indicates that all aromatic and triazole fragments are located in an isotropic environment.

An analysis of the ^1^H-NMR spectra recorded in D_2_O revealed a multiplication of the aromatic and H-triazole proton signals. So, as mentioned above, the H-triazole signal that appeared as a singlet in DMSO-*d*_6_ was observed as multiple signals in D_2_O between 8.04 and 7.49 ppm (see Figures S69, S78, S86, S94, and S107 in the Supplementary Materials). The same signal multiplication was observed for the aromatic fragments. For instance, in EDTA2PhCD dimer **A**, the signals at 7.25 and 6.77 ppm (Figure S69 in the Supplementary Materials) correspond to nonequivalent protons named Hc*i* and Hd*i* and the equivalent aromatic protons Hc and Hd appeared at 7.06 and 6.58 ppm, just like in DMSO-*d*_6_. NOESY experiments showed that the signals at 7.25 and 6.77 ppm gave cross-peaks with H-3 and H-5 protons (3.85, 3.79, and 3.64 ppm) present in the βCD inner cavity. Moreover, in the HMQC experiments, a proton signal appeared at 7.14 ppm, which overlapped with the previously described signals, giving a cross-peak with a carbon signal at 121.62 ppm (Figures S70 and S71 in the Supplementary Materials). That means that there is a nonequivalent triazole in this zone in addition to the multiple signals at 7.55–7.49 ppm in the proton spectrum, indicating a self-inclusion process presented in both βCD units, as illustrated in Figure S109 in the Supplementary Materials. A similar behavior was observed in D_2_O for the EDTA-βCD dimers (**A**–**C**, see Figures S79 and S87 in the Supplementary Materials) and EDTA-βCD dendrimers (**D** and **F**, see Figures S95 and S108 in the Supplementary Materials).

**Figure 1:**
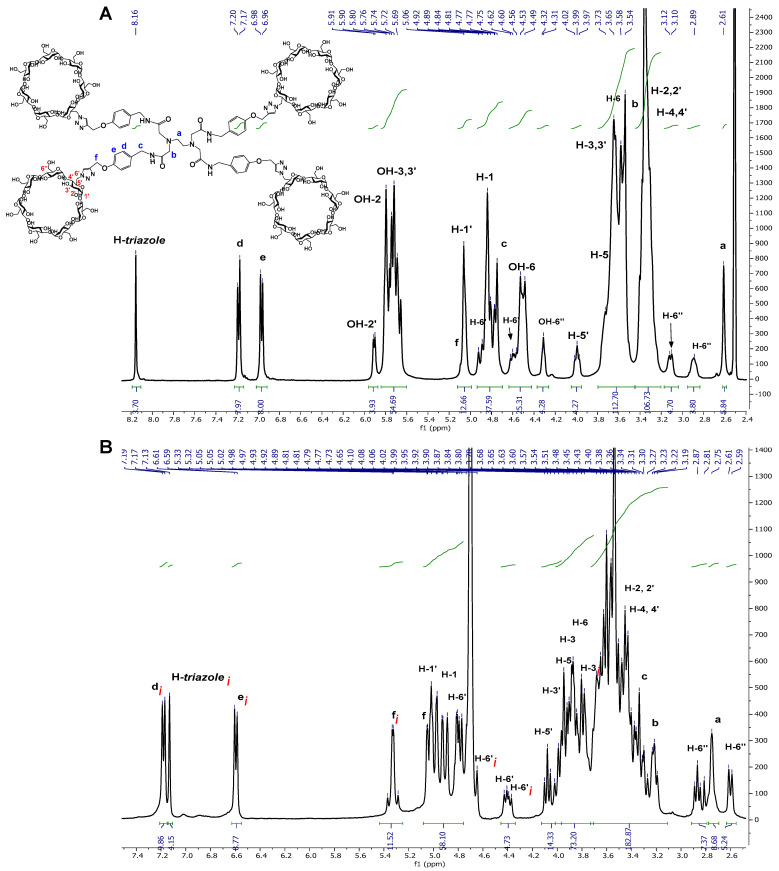
Comparison of ^1^H-NMR spectra of EDTA4BenCD dendrimer (**E**), (**A**) acquired in DMSO-*d*_6_ and (**B**) acquired in D_2_O.

**Figure 2:**
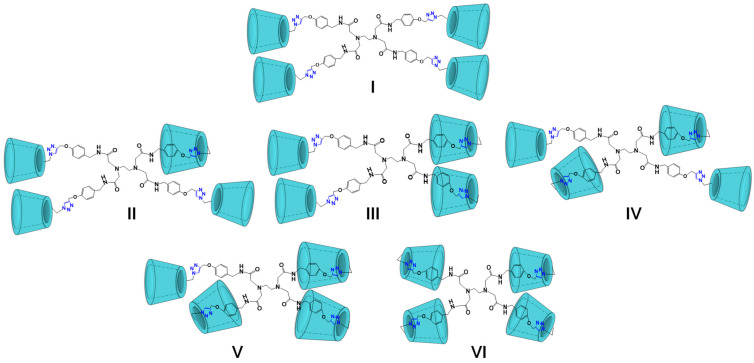
Possible conformations adopted for EDTA4BenCD dendrimer **E**, with 0 to 4 reversed cavities.

In the case of EDTA4BenCD dendrimer (**E**) in D_2_O, we observed a particular behavior in their ^1^H-NMR signals that differ significantly from what was previously described, since two symmetric doublets appeared at 7.19–7.17 ppm and 6.61–6.59 ppm due to the protons of the *para*-substituted aromatic ring and a singlet signal at 7.13 ppm corresponding to a single H-triazole signal; in other words, a multiplication of signals in the aromatic zone was observed for its analogs **D** and **F** (Figures S94 and S107 in SI), but not for **E** (see Figure 1B). In this way, the proton signals in the NMR spectrum of compound **E** are in an isotropic environment, but do not correspond to an extended conformation as presented in DMSO-*d*_6_, since in the NOESY experiments, all of the signals in the aromatic region gave cross-peaks with H-3 and H-5 protons of βCD (Figure S101 in SI). So, as we have previously observed, for a tetramer of PAMAM-βCD [31] in D_2_O, this type of platform can adopt six different conformations, as shown in Figure 2. However, given the symmetry of the signals in the aromatic zone and the cross-peaks observed in the NOESY experiments, we can affirm that in this EDTA4BenCD dendrimer (**E**), the conformation VI predominates, where the four βCD units are self-included.

### 2.3. Availability of the βCD Cavities in EDTA di-βCD (***A**–**C***) and EDTA G0-βCD Dendrimers (***D**–**F***)

Since we observed a self-inclusion process for both EDTA-βCD dimers (**A**–**C**) and EDTA-βCD dendrimers (**D**–**F**), and this phenomenon could be interpreted as a limited capacity for IC formation, which, in principle, could block at least one of the hosting βCD cavities [38,39,40] in the case of dimers or, in the worst scenario, the four βCD units in the dendrimers (e.g., for compound **E**), we decided to analyze the capacity of IC formation through a titration experiment, increasing the amounts of a host with high affinity for the βCD inner cavity. For this, we selected adamantane carboxylic acid (AdCOOH) and recorded the changes in the ^1^H-NMR spectra. We selected the platforms with more significative changes in their ^1^H-NMR in D_2_O, so that we analyzed the EDTA2TyrCD dimer (**C**) (see Figure S86 in SI) and EDTA4BenCD dendrimer (**E**) (Figure 1B).

**Figure 3:**
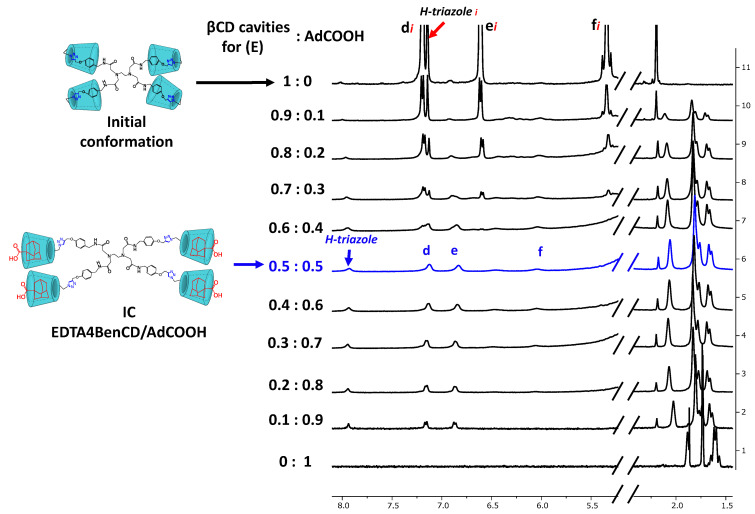
^1^H-NMR titration experiment of EDTA4BenCD dendrimer (**E**). The 5.0–8.2 ppm and 1.5–3.0 ppm regions are shown for the mixtures of **E** and AdCOOH in D_2_O with decreasing molar fraction from top to bottom, expressed with respect to the concentration in βCD cavities for EDTA4BenCD dendrimer (**E**) (XCD = [βCD]/([βCD] + [AdCOOH]). Total concentration [βCD] + [AdCOOH] = 7.3 mM.

For the sake of simplicity, each spectrum shown in Figure 3 was identified using the molar fraction XCD = [βCD]/([βCD] + [AdCOOH]; for example, the first spectrum with a 0.9 XCD molar ratio of EDTA4BenCD dendrimer (**E**) maintained the same signals as those observed for pure dendrimer **E** in D_2_O. This behavior was practically uniform up to a 0.6:0.4 ratio dendrimer (**E**):AdCOOH. Therefore, as the XCD ratio decreased according to the molar ratio, the spectrum shape became similar to the shape of the spectrum obtained in DMSO-*d*_6_ (see Figure 1A); thus, the H-triazole and Hd and He protons of the aromatic region appeared in an isotropic environment (see 0.5:0.5 ratio in Figure 3). Additionally, the progressive shift from up-field to down-field of the initially inverted Hf signal at 5.33 ppm (named Hfi in the first spectrum in Figure 3), assigned to the protons of methylene group next to triazole ring, was significantly affected by the self-inclusion process and reflected the reversion of this process for all of the βCD moieties.

**Figure 4:**
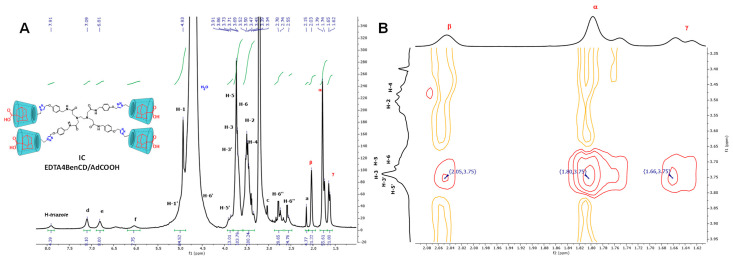
(**A**) ^1^H-NMR spectra of IC EDTA4BenCD dendrimer (**E**) with AdCOOH in D_2_O. (**B**) Amplification of aliphatic zone in the 2D NMR NOESY spectrum of IC EDTA4BenCD dendrimer (**E**) with AdCOOH in D_2_O.

Besides the well-defined signals in the aromatic region of the ^1^H-NMR spectrum of the IC of EDTA4BenCD dendrimer (**E**) with AdCOOH in the equimolar ratio shown in Figure 4A, between 3.91 and 3.34 ppm, we observed the progressive disappearance of multiple signals for H-3,3′, H-5,5′, H-6, and H-2,2′, 4,4′ protons (compared to the same region in Figure 1B), which reflected the reversal of the self-inclusion process described above. In order to reinforce this affirmation, long-range interactions were tracked through 2D NOESY NMR experiments (see Figure 4B and Figure S110 for dimer **C**). Only cross-peaks between the βCD inner protons (H-3,3′; H-5,5′) and those corresponding to AdCOOH (H-β; H-α; H-γ) were observed, which indicates that βCD cavities only hosted the AdCOOH molecules. All of the above observations confirmed the complete availability of the βCD host cavities for the AdCOOH guests, despite the initial reversed conformation in D_2_O.

In summary, we demonstrated that there is an 1:1 stoichiometry and an extended spatial conformation for dendrimer EDTA4BenCD (**E**), which forms a host–guest IC with AdCOOH in D_2_O, although this particular compound started exhibiting four self-included βCD units.

### 2.4. Determination of Quantitative Water Solubility for EDTA di-βCD (***A**–**C***) and EDTA G0-βCD Dendrimers (***D**–**F***)

Water solubility is a key property influencing behavior of one molecule in nature, science, and particularly when the design of new entities focuses on their potential biological applications [41]. Dendrimers have stood out as a novel type of material with a unique structure and suitable properties for multiple applications. In general, dendrimers can have a hydrophobic interior and are covered by a hydrophilic shell (e.g., carbohydrates) involved in interactions with water, promoting their solubilization [41,42,43,44]. 

In the case of our molecules, we noticed that the conjugation of the EDTA core with hydrophilic molecules, such as βCD moieties, significantly increased their water solubility. For this reason, the quantitative solubility in the water of the all platforms was determined by the method reported by Jozwiakowski and Connors [45], and the results are presented in Table 1. 

For dimers (**A**–**C**) an average solubility value of 1.589 g/mL was obtained, and for dendrimers (**D**–**F**), an average value of 1.606 g/mL was obtained. As can be observed, in terms of solubility values, there is no significant difference between both designs, which can be explained in the case of dimers (**A**–**C**) because although they only have two βCD units, the carboxylic acid functional groups that emerge from the core act synergistically with the βCD moieties, and in this way, they do not limit the solubility of the final platforms. Indeed, the platforms presented here significantly exceed the water solubility value of the native βCD (0.0185 g/mL) [46]. If we compare our novel platforms with the more soluble βCD commercial derivatives such as sulfobutylether-βCD (>0.5 g/mL), O-methyl-βCD (>0.5 g/mL), and 2-hydroxypropyl-βCD (0.6 g/mL) [46], all dimers (**A**–**C**) and dendrimers (**D**–**F**) exhibit higher solubility than all of them and show an additional advantage, namely, the ability to include two or four hydrophobic molecules depending on the selected design, which puts these designs above those previously reported.

### 2.5. Dynamic Light Scattering Size Analysis of the EDTA di-βCD (***A**–**C***) and EDTA-βCD Dendrimers (***D**–**F***)

**Figure 5:**
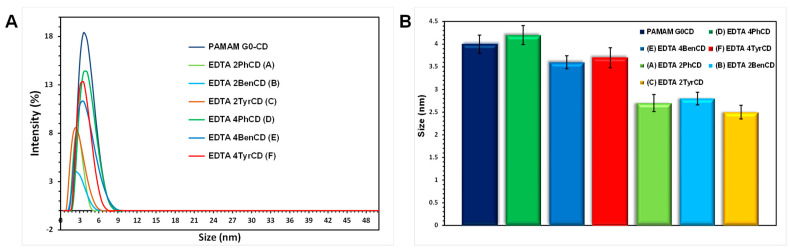
(**A**) Determination of particle size by DLS. (**B**) Comparison of the determined sizes for dendrimers EDTA-βCD (**D**–**F**) and dimers EDTA-βCD (**A**–**C**).

One goal for the design of dendrimers (**D**–**F**) based on EDTA-βCD is that they keep structurally similar properties compared to a classical PAMAM dendrimer. If we consider that dendrimers **D**–**F** are perfect analogs of PAMAM, the new EDTA-βCD dendrimers should have the same hydrodynamic size. In this regard, we determined the hydrodynamic diameter in water for dendrimers **D**–**F** by DLS measurements and the corresponding dimers of the intermediates from which they came (**A**–**C**). For a reference molecule, we took a PAMAM G0-βCD dendrimer previously reported by us [31]. For this molecule, the DLS measurement showed a size of 4 nm in diameter, as shown in Figure 5, so based on this parameter, we carried out a comparative analysis with the new dendrimeric platforms (**D**–**F**). The average size found for these new platforms was 3.85 nm, as shown in Figure 5B. The three EDTA-βCD analogs presented practically the same particle size, which confirms that there is no direct relationship between the spacer used between the EDTA core and the βCD units of the periphery with a change in the size of the final dendrimer, since for these three analogs (**D**–**F**), the particle size was practically the same as that of the reference molecule.

Regarding dimers **A**–**C**, we found an average particle size of 2.7 nm. These molecules, which only contain two βCD units, had a change of 1.15 nm with respect to the fully functionalized dendrimers (**D**–**F**), which represents a change of 30% in size between the dimer and its corresponding dendrimer. It is important to mention that during the analysis, we did not observe the formation of aggregates for any of the evaluated platforms.

### 2.6. Viability Studies

In order to study the in vitro behavior of EDTA-βCD dimers **A**–**C** and EDTA-βCD dendrimers **E**–**F** and to corroborate that these news designs are not undesirable toxic nanocarriers, we decided perform cell viability assays for all platforms. For this, these dendrimers were assessed against three different cancer cell lines: MCF-7, MDA-MB-231, and SK-LU-1, which were selected as specific cell lines due to the projection that these platforms may have as carriers in chemotherapeutics; and HeLa cells, which are commonly used as cellular model for preliminary screening of cytotoxicity [47], since the composition and morphology of engineered nanomaterials have been shown to influence their biological response. For this reason, these cells lines were selected as the model in this study. 

According to our previous experience in viability assays for modified dendrimers [48], in this evaluation, six different concentrations from 2 μM to 100 μM were tested, taking 100% of the viability to untreated cells. It was observed that at concentrations below 90 μM, neither platform caused the inhibition of the treated cells, so we focused on analyzing the highest concentration (100 μM) where we began to observe changes in the viability percentage. 

**Figure 6:**
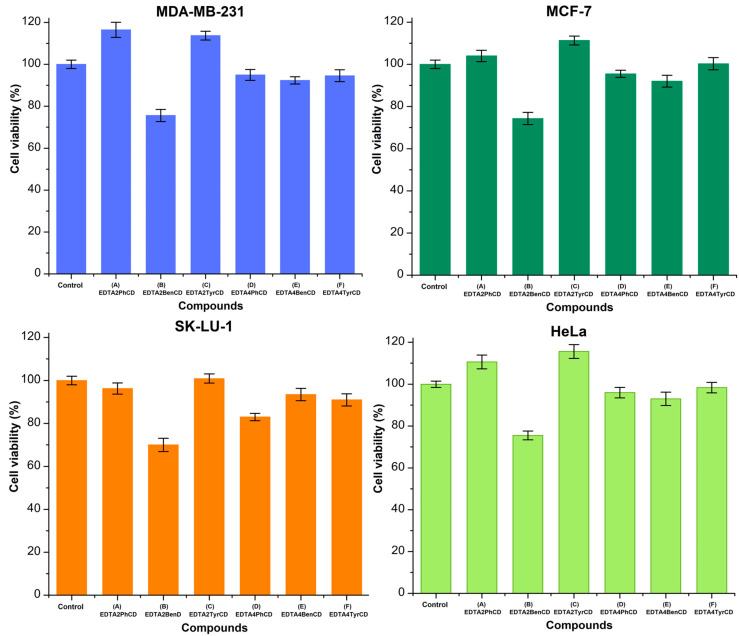
Cell viability in different cell lines for EDTA βCD molecules (**A**–**F**). The treated cells were cultured in six wells by concentration, and the experiments were performed independently three times. Data are means ± SD.

It is well known that low-generation PAMAM dendrimers (G0–3) containing a few free amino groups on the surface present neither cellular toxicity nor carrying capacity of molecules due to their open and flexible structure [48,49,50,51,52]. In this way, the design of dendrimers based on the EDTA-βCD (**D**–**F**) presented in this work can be considered as perfect analogs of the PAMAM G0 dendrimer, which, as described above, have the capacity to form ICs with defined stoichiometry. To corroborate the non-cytotoxic activity for platforms **D**–**F** and the corresponding dimers **A**–**C**, we tested them in the four cell lines mentioned above. The results are shown in Figure 6, where we can see the dimers **A**–**C** followed by their dendrimeric counterparts **D**–**F** until all of the platforms are covered. It is evident that, in general, platforms **A**, **C**, and **D**–**F** do not present any toxicity in the tested cell lines. Dimer **B** showed a particular behavior, since it induced between 15 and 20% inhibition in the tested cell lines, which may be due to its likely ability to complex ions that are important for cell growth [53,54]. However, at the tested concentration, it is far from being considered a cytotoxic compound. It is important to mention that this behavior was not reflected in the analogs **A** and **C** and can be related to the twistability of the tested spacers. 

According to our results, it is important to notice that none of the EDTA-CD platforms (**A**–**F**) show any significant inhibition percentage on the tested cell lines. In concordance with previous reports [48], for these new designs, the conjugation of βCD units on the platform surface prevents the interaction with the cell membrane, protecting it from being compromised, thereby avoiding cell toxicity. However, if we make a strict comparison between the dimers and the completely modified platforms, we can state with certainty that the latter produce practically non-toxic nanocarriers in all of their versions presented here. 

## 3. Materials and Methods

### 3.1. General Notes 

All of the starting materials were purchased from Merck Sigma-Aldrich Mexico and used without any further purification. Tyramine (Tyr), 4-aminophenol (Ph), 4-hydroxybenzylamine (Ben), di-*tert*-butyl dicarbonate (Boc_2_O), *N*,*N*-diisopropyl ethyl amine (DIPEA), β-cyclodextrin (βCD), *p*-toluenesulfonyl chloride (ClOTs), *p*-toluenesulfonic acid (HOTs), *N*,*N*-dimethylformamide anhydrous (DMF anh.), ammonium chloride (NH_4_Cl), sodium sulfate (Na_2_SO_4_), potassium carbonate (K_2_CO_3_), propargyl bromide solution ~80% in toluene, dimethylsulfoxide (DMSO), ascorbic acid (H_2_Asc), sodium bicarbonate (NaHCO_3_), potassium iodide (KI), sodium azide (NaN_3_), trifluoracetic acid (TFA), 1-ethyl-3-(3-dimethylaminopropyl)carbodiimide hydrochloride (EDC**·**HCl), *N*-hydroxybenzotriazole (HOBt), sodium hydroxide (NaOH), copper sulfate pentahydrate (CuSO_4_•5H_2_O), Tris[(1-benzyl-1H-1,2,3-triazol-4-yl)methyl]amine (TBTA), dichloromethane (CH_2_Cl_2_), ethyl acetate (EtOAc), tetrahydrofuran anhydrous (THF), hexane (Hex), methanol (MeOH), and acetone were purchased from Sigma-Aldrich (St. Louis, MO, USA). Distilled water was used in all experiments. Reactions were performed under a nitrogen atmosphere and monitored by analytical TLC on pre-coated silica gel 60 F254 plates (Aldrich) with detection carried out under UV light. Purifications by column chromatography were performed on silica gel at 60–200 μm (Sigma-Aldrich, St. Louis, MO, USA). Size exclusion chromatography (SEC) was performed using water as the eluent with resin Bio-Gel^®^ P-6 medium (BIO-RAD Laboratory, Hercules, CA, USA). Deuterated dimethyl sulfoxide (DMSO-*d*_6_) and deuterium oxide (D_2_O) with an isotopic purity of 99.9% was obtained from Cambridge Isotope Laboratories, Inc., Cambridge, UK. Tetramethylsilane (TMS), an internal NMR reference, was purchased from Merck Sigma-Aldrich Mexico. Then, ^1^H and ^13^C-DEPTQ NMR as well as 2D HMQC, COSY, and NOESY experiments were performed at 298 K on a Bruker Advance 400 MHz instrument. Chemical shifts (δ) are reported in ppm and coupling constants (*J*) in Hz. Multiplicities are reported using the following abbreviations: *s* = singlet, *d* = doublet, *t* = triplet, *br* = broad, and *m* = multiplet. Proton and carbon signal assignments are indicated in the spectra and reported in SI. Infrared spectra (IR) were recorded at room temperature using a Nicolet FT-5SX spectrometer equipped with an ATR detector with a diamond crystal. A sample of the tested compound was mechanically pressed onto the diamond crystal. Interferograms of 64 scans were performed in averages for each spectrum at a resolution of 4 cm^−1^. DART, ESI, and MALDI-TOF mass measurements were recorded on a JEOL JMS-AX505-HA instrument (Peabody, MA, USA) and on a Bruker Daltonics Flex Analysis instrument (Bruker, Beerlika, MA, USA), respectively. Finally, 2,5-Dihydroxybenzoic acid (DHB) was used as a matrix for MALDI-TOF. 

### 3.2. Synthetic Procedures 

#### 3.2.1. Mono-tosyl-βCD (*m*OTsβCD)

According to the procedures previously informed [44] with some brief modifications, in a round flask, HOTs (1.92 g, 10.1 mmol) and ClOTs (7.5 g, 39 mmol) were dissolved in 50 mL of CH_2_Cl_2_ and stirred at room temperature for 12 h. Then, the reaction mixture was filtered, and the filtrate was concentrated under reduced pressure. The residue was recrystallized by triplicate with cold Hex and the product Ts_2_O was filtered and allowed to dry under vacuum. Afterwards, in a round flask, βCD (5.75 g, 5.1 mmol) and Ts_2_O (2.48 g, 7.6 mmol) were dissolved in 150 mL of H_2_O with vigorous stirring for 2 h at room temperature; after this time, a 2.5 M aqueous NaOH solution was added. Then, the mixture was stirred for an additional 10 min. Subsequently, the unreacted Ts_2_O was filtered, and the recovered liquid phase was adjusted with solid NH_4_Cl until pH = 8 in an ice bath at 0 °C to give a solid white precipitate. The remanent liquid was decanted and an additional portion of 100 mL of cold water was added; then, the solid was filtered under vacuum. The solid residue was recrystallized three times in a mixture of acetone:water (in the proportion 90:10). Finally, the white solid was recrystallized one time from MeOH to obtain pure *m*OTsβCD (4.5 g, 3.5 mmol, 69% yield). ^1^H-NMR (400 MHz, DMSO-*d*_6_, δ ppm): 7.75 (b, 2H) a, 7.44 (b, 2H) b, 5.83 (d, *J* = 6.4 Hz, 1H) OH2′, 5.78 (b, 6H) OH2, 5.71 (b, 7H) OH3, 4.84 (d, *J* = 3.9 Hz, 6H) H1, 4.76 (d, *J* = 3.9 Hz, 1H) H1′, 4.50 (m, 6H) OH6, 4.35 (m, 2H) H6′ab, 4.19 (m, 1H) H5′, 3.65 (m, 12H) H6ab, 3.60 (b, 7H) H3, 3.51 (m, 7H) H5, 3.30 (m, 7H) H2, 3.22 (m, 7H) H4, 2.42 (d, 3H) c. ^13^C-DEPTQ NMR (101 MHz, DMSO-*d*_6_, δ ppm): 145.25, 133.09, 130.29, 128.03, 102.39, 101.74, 81.95, 81.21, 73.43, 73.16, 72.84, 72.48, 70.17, 69.36, 60.28, 21.62. MALDI-TOF (*m*/*z*): 1311.59 [M + Na]^+^.

#### 3.2.2. Mono-azide-βCD (*m*N_3_βCD) 

According to the literature [25], in a round flask, *m*OTsβCD (2.8 g, 2.17 mmol), NaN_3_ (0.422 g, 6.52 mmol), and KI (0.180 g, 1.09 mmol) were dissolved in 8 mL of DMF anh. The reaction mixture was stirred at 80 °C for 48 h. After this time, DMF was evaporated under reduced pressure and the residue was recrystallized in a mixture of H_2_O:acetone (10:90) and allowed to dry under vacuum to obtain *m*-N_3_βCD as a white solid (1.2 g, 1.03 mmol, 95% yield). ^1^H-NMR (400 MHz, DMSO-*d*_6_, δ ppm): 5.74 (m, 7H) OH2, 5.67 (m, 6H) OH3, 5.62 (d, *J* = 2.4 Hz, 1H) OH3′, 4.88 (d, *J* = 3.5 Hz, 1H) H1′, 4.83 (m, 6H) H1, 4.48 (m, 6H) OH6, 3.77 (m, 2H) H6′, 3.68 (m, 12H) H6, 3.60 (m, 7H) H3, 3.55 (m, 7H) H5, 3.39 (m, 7H) H4, 3.29 (m, 7H) H2. ^13^C-DEPTQ NMR (101 MHz, DMSO-*d*_6_, δ ppm): 102.38, 102.04, 83.41, 81.99, 73.50, 73.30, 72.85, 72.67, 72.46, 70.63, 60.30, 51.53. MALDI-TOF (*m*/*z*): 1182.76 [M + Na]^+^.

#### 3.2.3. General Procedure for the Synthesis of *Tert*-butyl Carbamate Intermediates (**4**–**6**)

The synthesis of the intermediates **4**–**6** was carried out according to previously reported procedures [37,55]. Under an inert atmosphere, a solution of Boc_2_O (1.2 eq) in 10 mL of THF was added dropwise to a suspension prepared with 1 eq. of 4-aminophenol (**1**), 4-hydroxybenzylamine (**2**), or 4-tyramine (**3**) in 30 mL of THF at 0 °C over a period of 30 min with vigorous stirring. The reaction mixture was maintained at 0 °C for an additional period of 20 min. Then, it was allowed to slowly warm to rt and stirred overnight. The complete disappearance of amines was observed by TLC monitoring, and then the solvent was removed under reduced pressure. The semisolid residue was dissolved in EtOAc and the organic layer was washed once with a saturated solution of NaHCO_3_ and twice with brine, dried over Na_2_SO_4_. Then, the solvent was removed under reduced pressure. The corresponding crude product was purified by flash chromatography (silica gel, CH_2_Cl_2_ as eluent). 

##### *Tert*-butyl(4-hydroxyphenyl)carbamate (**4**)

The intermediary (**1**) was obtained as a white solid (98% yield). R_f_ = 0.3 (CH_2_Cl_2_). ^1^H NMR (400 MHz, DMSO-*d*_6_, δ ppm): 9.04 (s, 1H, -NH-), 8.98 (br, 1H, Ph-OH), 7.21 (d, *J* = 8.2 Hz, 2H, Hb), 6.64 (d, *J* = 8.2 Hz, 2H, Hc), 1.45 (s, 9H, Ha); ^13^C-DEPTQ NMR (101 MHz, DMSO-*d*_6_, δ ppm): 153.80, 153.30, 131.81, 120.85, 115.81, 79.22, 29.00; IR (ATR, cm^−1^): 3400 O-H (st), 2988-2975 CH_3_ (st), 1885 aromatic (st), 1695 C=O (st); MS (DART^+^) *m*/*z*: [M + H]^+^ calcd for C_11_H_15_NO_3_ 209.24, found 210.

##### *Tert*-butyl(4-hydroxybenzyl)carbamate (**5**)

The intermediary (**2**) was obtained as a colorless oil (95% yield). R_f_ = 0.35 (CH_2_Cl_2_). ^1^H NMR (400 MHz, DMSO-*d*_6_, δ ppm): 9.25 (s, 1H, Ph-OH), 7.25 (m, 1H, -NH-), 7.03 (d, *J* = 8.6 Hz, 2H, Hc), 6.70 (d, *J* = 8.6 Hz, 2H, Hd), 4.01 (m, 2H, Hb), 1.39 (s, 9H, Ha); ^13^C-DEPTQ NMR (101 MHz, DMSO-*d*_6_, δ ppm): 156.90, 156.52, 131.16, 129.06, 115.72, 78.39, 43.71, 29.07; IR (ATR, cm^−1^): 3338 O-H (st), 2977 CH_2_ (st), 2817 CH_3_ (st), 1882 aromatic (st), 1675 C=O (st); MS (DART^+^) *m*/*z*: [M + H]^+^ calcd for C_12_H_17_NO_3_ 223.27, found 224.

##### *Tert*-butyl(4-hydroxyphenetyl)carbamate (**6**)

The intermediary (**3**) was obtained as colorless crystals (98% yield). R_f_ = 0.5 (CH_2_Cl_2_). ^1^H NMR (400 MHz, DMSO-*d*_6_, δ ppm): 9.16 (s, 1H, Ph-OH), 6.98 (d, *J* = 8.3 Hz, 2H, Hd), 6.82 (m, 1H, -NH-), 6.68 (d, *J* = 8.3 Hz, 2H, He), 3.05 (m, 2H, Hb), 2.56 (t, *J* = 6.9 Hz, 2H, Hc), 1.37 (s, 9H, Ha); ^13^C-DEPTQ NMR (101 MHz, DMSO-*d*_6_, δ ppm): 156.38, 156.30, 130.27, 130.24, 115.86, 78.23, 42.70, 36.52, 29.08; IR (ATR, cm^−1^): 3370 O-H (st), 2981, 2939 CH_2_ (st), 2864 CH_3_ (st), 1892 aromatic (st), 1683 C=O (st); MS (DART^+^) *m*/*z*: [M + H]^+^ calcd for C_13_H_19_NO_3_ 237.30, found 238.

#### 3.2.4. General Procedure for the Synthesis of *tert*-butyl 4-(Prop-2-yn-1-yloxy)carbamates Intermediaries (**7**–**9**)

The synthesis of alkyne intermediaries **7**–**9** was carried out according to previously reported procedures, with brief modifications [27,28]**.** Briefly, 1 eq of the corresponding *tert-*butyl carbamates intermediaries (**4**–**6**) was dissolved in 250 mL of DMF and K_2_CO_3_ 1.4 eq was added. The reaction mixture was heated at 80 °C for a period of 1 h. Afterwards, 1.3 eq of propargyl bromide solution was added and the resulting mixture was refluxed for 24 h. Then, the reaction mixture was cooled to rt, filtered, and the filtrate was evaporated under reduced pressure. The resulting brown oil was dissolved in 150 mL of CH_2_Cl_2_ and the solution was extracted with sodium hydroxide (NaOH 1 M, 200 mL) and then with water (3 × 50 mL). The organic layer was dried over Na_2_SO_4_ and the solvent was evaporated under reduced pressure. The crude product was purified by flash chromatography (silica gel, CH_2_Cl_2_ as eluent).

##### *Tert*-butyl (4-(Prop-2-yn-1-yloxy)phenyl)carbamate (**7**)

The intermediary (**7**) was obtained as a pale-yellow solid (92% yield). R_f_ = 0.6 (CH_2_Cl_2_). ^1^H NMR (400 MHz, DMSO-*d*_6_, δ ppm): 9.17 (s, 1H, -NH-), 7.36 (d, *J* = 8.7 Hz, 2H, Hb), 6.89 (d, *J* = 8.7 Hz, 2H, Hc), 4.73 (d, 2H, Hd), 3.54 (t, 1H, He), 1.47 (s, 9H, Ha); ^13^C-DEPTQ NMR (101 MHz, DMSO-*d*_6_, δ ppm): 153.71, 153.13, 134.10, 120.44, 115.88, 79.53, 78.82, 56.40, 28.96; IR (ATR, cm^−1^): 3361.84 N-H (st), 3283 C-H alkyne (st), 2983 CH_3_ (st), 2134 C≡C (st),1870 aromatic (st), 1690 C=O (st); MS (DART^+^) *m*/*z*: [M + H]^+^ calcd for C_14_H_19_NO_3_ 249.31, found 250.

##### *Tert*-butyl (4-(Prop-2-yn-1-yloxy)benzyl)carbamate (**8**)

The intermediary (**8**) was obtained as yellow oil (90% yield). R_f_ = 0.63 (CH_2_Cl_2_). ^1^H NMR (400 MHz, DMSO-*d*_6_, δ ppm): 7.33 (t, 1H, -NH-), 7.17 (d, *J* = 8.4 Hz, 2H, Hc), 6.92 (d, *J* = 8.4 Hz, 2H, Hd), 4.77 (d, 2H, He), 4.06 (d, 2H, Hb), 3.54 (m, 1H, Hf), 1.39 (s, 9H, Ha); ^13^C-DEPTQ NMR (101 MHz, DMSO-*d*_6_, δ ppm): 156.81, 156.55, 133.76, 128.98, 115.40, 78.90, 78.50, 56.15, 43.57, 29.06; IR (ATR, cm^−1^): 3351.21 N-H (st), 3302 C-H alkyne (st), 2123 C≡C (st), 2978 CH_2_ (st), 2869 CH_3_ (st), 1885 aromatic (st), 1677 C=O (st); MS (DART^+^) *m*/*z*: [M + H]^+^ calcd for C_15_H_19_NO_3_ 261.32, found 262.

##### *Tert*-butyl (4-(Prop-2-yn-1-yloxy)phenetyl)carbamate (**9**)

The intermediary (**9**) was obtained as a yellow liquid (95% yield). R_f_ = 0.65 (CH_2_Cl_2_). ^1^H NMR (400 MHz, DMSO-*d*_6_, δ ppm): 7.13 (d, *J* = 8.2 Hz, 2H, Hd), 6.92 (d, *J* = 8.2 Hz, 2H, He), 6.85 (m, 1H, -NH-), 4.76 (d, 2H, Hf), 3.53 (t, 1H, Hg), 3.14 (m, 2H, Hb), 2.66 (m, 2H, Hc), 1.38 (s, 9H, Ha); ^13^C-DEPTQ NMR (101 MHz, DMSO-*d*_6_, δ ppm): 156.41, 156.32, 132.94, 130.34, 115.46, 80.19, 78.78, 78.26, 56.12, 42.52, 35.42, 29.06; IR (ATR, cm^−1^): 3357.65 N-H (st), 3292 C-H alkyne (st), 2121 C≡C (st), 2977, 2932 CH_2_ (st), 2868 CH_3_ (st),1884 aromatic (st), 1693 C=O (st); MS (DART^+^) *m*/*z*: [M + H]^+^ calcd for C_16_H_21_NO_3_ 275.35, found 276.

#### 3.2.5. General Procedure for the Synthesis of 4-(Prop-2-yn-1-yloxy)amines Intermediaries (**10**–**12**)

The *N*-boc deprotection of intermediaries **7**–**9** was carried out according to previously reported procedures with some modifications [29,30]. A solution of 10 mmol of **7**, **8**, or **9** dissolved in 7 mL CH_2_Cl_2_ was prepared in an ice bath with vigorous stirring, and then 7 mL of TFA was added dropwise to the reaction mixture and kept for 30 min. After this time, the ice bath was removed and the corresponding mixture was allowed to reach rt and stirred for 0.5 h for **10**, 2.5 h for **11**, and 3 h for **12**. The complete disappearance of the *N*-boc-protected compound was monitored by TLC; then, 150 mL of water was added to the reaction mixture and the organic phase was separated. The aqueous layer was treated with a solution of 5 M of NaOH until it reached a pH of 12 and was extracted with three portions of 50 mL of CH_2_Cl_2_. The organic phase was dried over anhydrous Na_2_SO_4_, and then the solvent was evaporated under reduced pressure. The corresponding amines **10**–**12** were obtained without any further purification.

##### 4-(Prop-2-yn-1-yloxy)aniline (**10**)

The compound **10** was obtained as a red oil (95% yield). Rf = 0.25 (CH_2_Cl_2_/MeOH (9:1 *v*:*v*)). ^1^H NMR (400 MHz, DMSO-*d*_6_, δ ppm): 6.72 (d, *J* = 8.7 Hz, 2H, Ha), 6.52 (d, *J* = 8.7 Hz, 2H, Hb), 4.68 (br, 2H, -NH_2_), 4.61 (d, 2H, Hc), 3.48 (t, 1H, Hd); ^13^C-DEPTQ NMR (101 MHz, DMSO-*d*_6_, δ ppm): 149.32, 143.96, 116.84, 115.52, 80.82, 78.40, 56.94; IR (ATR, cm^−1^): 3426, 3352 N-H (st), 3275 C-H alkyne (st), 2118 C≡C (st), 1857 aromatic (st); MS (DART^+^) *m*/*z*: [M + H]^+^ calcd for C_9_H_9_NO 147.17, found 148.

##### [4-(Prop-2-yn-1-yloxy)phenyl]methanamine (**11**)

The compound **11** was obtained as a white solid (95% yield). Rf = 0.3 (CH_2_Cl_2_/MeOH (9:1 *v:v*)). ^1^H NMR (400 MHz, DMSO-*d*_6_, δ ppm): 7.27 (d, *J* = 8.6 Hz, 2H, Hb), 6.92 (d, *J* = 8.6 Hz, 2H, Hc), 4.75 (d, 2H, Hd), 3.65 (s, 2H, Ha), 3.54 (t, 1H, He), 2.51 (br, 2H, -NH_2_) overlapped with DMSO; ^13^C-DEPTQ NMR (101 MHz, DMSO-*d*_6_, δ ppm): 156.48, 137.90, 128.89, 115.28, 80.26, 78.83, 56.15, 45.84; IR (ATR, cm^−1^): 3366 N-H (st), 3284.65 C-H alkyne (st), 2918, 2863 CH_2_ (st), 2118.66 C≡C (st), 1888.6 aromatic (st); MS (DART^+^) *m*/*z*: [M + H]^+^ calcd for C_10_H_11_NO 161.2, found 162.

##### 2-(4-(Prop-2-yn-1-yloxy)phenyl)ethan-1-amine (**12**)

The compound **12** was obtained as a yellow liquid (93% yield). Rf = 0.3 (CH_2_Cl_2_/MeOH (9:1 *v:v*)). ^1^H NMR (400 MHz, DMSO-*d*_6_, δ ppm): 7.14 (d, *J* = 8.2 Hz, 2H, Hc), 6.91 (d, *J* = 8.2 Hz, 2H, Hd), 4.75 (d, 2H, He), 3.55 (t, 1H, Hf), 2.76 (m, 2H, Ha), 2.60 (m, 2H, Hb), 2.19 (br, 2H, -NH_2_); ^13^C-DEPTQ NMR (101 MHz, DMSO-*d*_6_, δ ppm): 156.23, 133.94, 130.34, 115.43, 80.21, 78.85, 56.13, 44.65; IR (ATR, cm^−1^): 3356 N-H (st), 3284 C-H alkyne (st), 2926, 2863 CH_2_ (st), 2119 C≡C (st), 1886 aromatic (st); MS (DART^+^) *m*/*z*: [M + H]^+^ calcd for C_11_H_13_NO 175.23, found 176.

#### 3.2.6. General Procedure for the Synthesis of Disubstituted EDTA Alkynes (**13**–**15**)

According to the previously reported procedures [29,56], EDTA dianhydride 1 eq and DIPEA 2.2 eq were suspended in 5 mL of DMF anh. at 0 °C and the reaction mixture was stirred for 20 min. Subsequently, a solution of compound **10**, **11**, or **12** (2.2 eq) in 1 mL of DMF anh. was added dropwise, and the reaction mixture was stirred at 0 °C for an additional period of 10 min and then at rt for 12 h. The solvent was removed under reduced pressure. Finally, the corresponding product was purified by recrystallization from MeOH. 

##### Disubstituted EDTA Alkyne (**13**)

The di alkyne **13** was obtained as a white solid (99% yield). ^1^H NMR (400 MHz, DMSO-*d*_6_, δ ppm): 12.34 (br, 2H, –COOH), 9.98 (s, 2H, –CONH–), 7.56 (d, *J* = 8.3 Hz, 4H, Hc), 6.92 (d, *J* = 8.3 Hz, 4H, Hd), 4.75 (d, 4H, He), 3.55 (s, 2H, Hf), 3.44–3.18 (m, 8H, Hb,b′ overlapped with H_2_O), 2.83 (s, 4H, Ha); ^13^C-DEPTQ NMR (101 MHz, DMSO-*d*_6_, δ ppm): 173.78, 169.97, 153.90, 133.37, 121.35, 115.72, 80.19, 78.90, 58.87, 56.27, 53.02, 49.41; IR (ATR, cm^−1^): 3329 C(O)O–H (st), 3279, 3248 N–H (st), 2123.39 C≡C (st), 1880 aromatic (st), 1697 C=O (st); MS (ESI^+^) *m*/*z*: [M]^+^ calcd for C_28_H_30_N_4_O_8_ 550.57, found 551.3.

##### Disubstituted EDTA Alkyne (**14**)

The di alkyne **14** was obtained as a white solid (99% yield). ^1^H NMR (400 MHz, DMSO-*d*_6_, δ ppm): 8.42 (t, 2H, –CONH–), 7.20 (d, *J* = 8.3 Hz, 4H, Hd), 6.93 (d, *J* = 8.3 Hz, 4H, He), 4.76 (d, 4H, Hf), 4.22 (d, 4H, Hc), 3.54 (t, 2H, Hg), 3.37–3.27 (m, 8H, Hb,b′ overlapped with H_2_O), 2.73 (m, 4H, Ha); ^13^C-DEPTQ NMR (101 MHz, DMSO-*d*_6_, δ ppm): 173.42, 171.24, 156.86, 133.02, 129.23, 115.42, 80.15, 78.92, 58.26, 56.16, 56.08, 53.12, 42.14; IR (ATR, cm^−1^): 3263 N–H (st), 3081 C–H alkyne (st), 2116 C≡C (st), 1885 aromatic (st), 1655 C=O (st); MS (ESI^+^) *m*/*z*: [M]^+^ calcd for C_30_H_34_N_4_O_8_ 578.62, found 579. 

##### Disubstituted EDTA Alkyne (**15**)

The di alkyne **15** was obtained as a white solid (99% yield). ^1^H NMR (400 MHz, DMSO-*d*_6_, δ ppm): 7.96 (s, 2H, –CONH–), 7.20 (d, *J* = 8.21 Hz, 4H, He), 6.95 (d, *J* = 8.22 Hz, 4H, Hf), 4.77 (d, 4H, Hg), 3.56 (t, 2H, Hh), 3.36 (m, 8H, Hc overlapped with H_2_O), 3.01–2.97 (m, 4H, Hb,b’), 2.86 (s, 4H, Hd), 2.81 (m, 4H, Ha); ^13^C-DEPTQ NMR (101 MHz, DMSO-*d*_6_, δ ppm): 173.68, 156.79, 131.01, 130.50, 115.74, 78.97, 78.85, 59.51, 56.15, 50.92, 33.24; IR (ATR, cm^−1^): 3390 C(O)O–H (st), 3250 N-H (st), 3215 C–H alkyne (st), 2124 C≡C (st), 1888 armatic (st), 1630 C=O (st); MS (ESI^+^) *m*/*z*: [M]^+^ calcd for C_32_H_38_N_4_O_8_ 606.68, found 606.9. 

#### 3.2.7. General Procedure for the Synthesis of Tetrasubstituted EDTA G0-alkyne (**16**–**18**)

The synthesis of compounds **16–18** was performed according to a previously reported method with some modifications [29,31]. A solution of 1 eq of compound **13**, **14**, or **15**, EDC·HCl (2.5 eq), and HOBt (2 eq) in DMF (4 mL) was stirred for 2 h at rt, protected from light. Then, according to the desired alkyne, a solution of **10**, **11**, or **12** (3 eq) in 2 mL of DMF was added dropwise. The resulting mixture was kept in the dark with stirring for 24 h at rt. After evaporation of the solvent under vacuum, the corresponding pure compound was obtained after recrystallization from cold MeOH three times. 

#### Tetrasubstituted EDTA G0-Alkyne (**16**)

The tetrasubstituted alkyne **16** was obtained as a white powder (98% yield). ^1^H NMR (400 MHz, DMSO-*d*_6_, δ ppm): 10.10 (t, 4H, –CONH–), 7.54 (d, *J* = 8.3 Hz, 8H, Hc), 6.90 (d, *J* = 8.3 Hz, 8H, Hd), 4.74 (d, 8H, He), 3.55 (t, 4H, Hf), 3.47 (m, 8H, Hb), 2.87 (s, 4H, Ha); ^13^C-DEPTQ NMR (101 MHz, DMSO-*d*_6_, δ ppm): 170.12, 153.98, 133.30, 121.60, 115.71, 80.17, 78.91, 59.35, 56.37, 53.51; IR (ATR, cm^−1^): 3390 N-H (st), 3271 C–H alkyne (st), 2923, 2832 CH_2_ (st), 2790 C–H (st), 2116 C≡C (st), 1894 aromatic (st), 1729 C=O (st); MS (ESI^+^) *m*/*z*: [M + Na]^+^ calcd for C_46_H_44_N_6_O_8_Na 840.89, found 840.

#### Tetrasubstituted EDTA G0-Alkyne (**17**)

The tetrasubstituted alkyne **17** was obtained as a beige powder (98% yield). ^1^H NMR (400 MHz, DMSO-*d*_6_, δ ppm): 7.20 (d, *J* = 8.32 Hz, 8H, Hd), 6.91 (d, *J* = 8.32 Hz, 8H, He), 4.76 (m, 16H, Hf,c), 3.55 (m, 12H, Hg,b), 2.61 (s, 4H, Ha); ^13^C-DEPTQ NMR (101 MHz, DMSO-*d*_6_, δ ppm): 171.11, 157.13, 130.57, 129.89, 115.46, 80.07, 79.00, 56.43, 56.15, 52.46, 41.47; IR (ATR, cm^−1^): 3390 N-H (st), 3271 C–H alkyne (st), 2966, 2923 CH_2_ (st), 2832 C–H (st), 2116 C≡C (st), 1894 aromatic (st), 1729 C=O (st); MS (ESI^+^) *m*/*z*: [M]^+^ calcd for C_50_H_52_N_6_O_8_ 865, found 865.6.

#### Tetrasubstituted EDTA G0-Alkyne (**18**)

The tetrasubstituted alkyne **18** was obtained as a white powder (98% yield). ^1^H NMR (400 MHz, DMSO-*d*_6_, δ ppm): 8.18 (t, 4H, –CONH–), 7.12 (d, *J* = 8.22 Hz, 8H, He), 6.89 (d, *J* = 8.21 Hz, 8H, Hf), 4.74 (d, 8H, Hg), 3.54 (t, 4H, Hh), 3.30 (m, 8H, Hc), 3.06 (s, 8H, Hb), 2.68 (m, 8H, Hd), 2.53 (m, 4H, Ha); ^13^C-DEPTQ NMR (101 MHz, DMSO-*d*_6_, δ ppm): 170.92, 156.43, 132.82, 130.30, 115.48, 80.21, 78.88, 59.11, 56.12, 53.60, 40.12, 35.22; IR (ATR, cm^−1^): 3300 N–H (st), 3280 C–H alkyne (st), 2930, 2879 CH_2_ (st), 2832 C–H (st), 2130 C≡C (st), 1883 aromatic (st); MS (ESI^+^) *m*/*z*: [M + Na]^+^ calcd for C_54_H_59_N_6_O_8_Na 943.09, found 943.

#### 3.2.8. General Procedure for the Synthesis of Dendritic Compounds EDTA di-βCD (**A**, **B**, **C**)

A solution of CuSO_4_•5H_2_O (0.54 eq), H_2_Asc (1.62 eq) and TBTA (0.54 eq) in 2 mL of DMSO was added dropwise to a solution of disubstituted EDTA alkyne **16**, **17**, or **18** (1 eq) with 2.4 eq of ***m*-N_3_βCD** dissolved in 8 mL of DMSO previously degassed by bubbling with N_2_. The reaction mixture was heated to 80 °C with vigorous stirring under N_2_ atmosphere for 12 h. At the end of this period, the reaction mixture was cooled and precipitated into cold acetone (200 mL). The corresponding formed solid was filtered and purified by SEC through Bio-Gel^®^ P-6 medium using water as eluent. The adequate fraction was lyophilized to obtain the corresponding dendritic compounds EDTA di-βCD.

##### EDTA2PhCD (**A**)

The final compound **A** was obtained as a beige solid (90% yield). ^1^H NMR (400 MHz, DMSO-*d*_6_, δ ppm): 10.04 (br, 2H, –COOH), 8.34, (s, 2H, –NH–), 8.15 (s, 2H, H-triazole), 7.56 (d, 4H, Hc), 6.97 (d, 4H, Hd), 5.72 (br, 26H, OH-2,2′, OH-3,3′), 5.10 (s, 4H, He), 5.05 (s, 2H, H-1′), 4.90–4.77 (m, 18H, H-6, H-1, H-6′), 4.71–4.61 (br, 4H, H-6′, OH-6), 4.11 (br, 2H, OH-6″), 3.99 (m, 2H, H-5), 3.79–3.55 (m, 60H, H-6, H-3,3′, H-5), 3.36–3.32 (m, 42H, H-4,4′, H-2,2′ overlapped with H_2_O), 3.12 (m, 4H, H-6″), 3.04 (m, 8H, Hb,b′), 2.92–2.83 (m, 6H, H-6″, Ha); ^13^C-DEPTQ NMR (101 MHz, DMSO-*d*_6_, δ ppm): 171.18, 161.67, 143.45, 133.02, 132.94, 126.15, 121.44, 115.49, 102.70, 102.08, 84.19, 82.37, 82.17, 81.83, 73.93, 73.25, 72.85, 70.72, 60.66, 48.35; IR (ATR, cm^−1^): 3338 O–H (st), 2927 CH_2_ (st), 1026 C–O (st);); MS (MALDI-TOF^+^) *m*/*z*: [M + 4H]^+^ calcd for C_112_H_172_N_10_O_76_ 2874.57, found 2874.33.

##### EDTA2BenCD (**B**)

The final compound **B** was obtained as a white solid (85% yield). ^1^H NMR (400 MHz, DMSO-*d*_6_, δ ppm): 8.23 (br, 2H, –NH–), 8.15 (s, 2H, H-triazole), 7.18 (d, 4H, Hd), 6.98 (m, 4H, He), 5.73 (br, 26H, OH-2,2′, OH-3,3′), 5.07 (s, 4H, Hf), 5.06 (s, 2H, H-1′), 4.84–4.73 (m, 18H, H-6, H-1, H-6′), 4.59 (br, 12H, OH-6), 4.22 (br, 2H, OH-6″, Hc), 3.99 (m, 2H, H-5′), 3.64–3.58 (m, 60H, H-6, H-3,3’, H-5), 3.36–3.32 (m, 43H, H-4,4′, H-2,2′ overlapped with H_2_O), 3.13 (m, 4H, H-6″), 2.91 (m, 8H, H-6″, Hb,b′), 2.74 (br, 4H, Ha); ^13^C-DEPTQ NMR (101 MHz, DMSO-*d*_6_, δ ppm): 171.96, 162.59, 143.70, 132.38, 130.36, 130.29, 125.23, 115.93, 103.02, 102.81, 82.39, 74.00, 72.83, 60.65, 52.72, 51.54, 50.97, 45.37; IR (ATR, cm^−1^): 3338 O–H (st), 2925 CH_2_ (st), 1025 C–O (st); MS (MALDI-TOF^+^) *m*/*z*: [M + Na]^+^ calcd for C_114_H_172_N_10_O_76_Na 2921.62, found 2921.33

##### EDTA2TyrCD (**C**)

The final compound **C** was obtained as a beige solid (99% yield). ^1^H NMR (400 MHz, DMSO-*d*_6_, δ ppm): 8.17 (br, 2H, H-triazole), 7.22 (br, 4H, He), 7.0 (m, 4H, Hf), 5.79 (br, 26H, OH-2,2′, OH-3,3′), 5.09 (s, 4H, Hg), 5.03 (s, 2H, H-1′), 4.87–4.77 (m, 18H, H-6, H-1, H-6′), 4.62 (br, 4H, OH-6, OH-6″), 3.99 (m, 2H, H-5), 3.64–3.56 (m, 60H, H-6, H-3,3′, H-5), 3.36–3.32 (m, 43H, Hc, H-4,4′, H-2,2′ overlapped with H_2_O), 3.08 (m, 4H, H-6″), 2.85 (m, 8H, H-b,b′,d,a); ^13^C-DEPTQ NMR (101 MHz, DMSO-*d*_6_, δ ppm): 171.32, 162.38, 143.35, 132.97, 130.60, 130.56, 125.16, 115.67, 102.73, 101.86, 84.25, 82.86, 82.15, 82.08, 73.84, 73.27, 72.55, 70.99, 60.70, 51.89, 51.06, 50.03; IR (ATR, cm^−1^): 3282 O–H (st), 2926 CH_2_ (st), 1023 C–O (st); MS (ESI-TOF^+^) *m*/*z*: [M + 2K]^+^ calcd for C_116_H_174_N_10_O_76_K_2_ 3002.86, found 1500.

#### 3.2.9. General Procedure for the Synthesis of EDTA-βCD Dendrimers (**D**–**F**) 

According to the procedure described above for dendritic compounds **EDTA di-βCD**, a corresponding solution of 1 eq of EDTA G0-alkyne **16**, **17**, or **18** with 4.4 eq of ***m*-N_3_βCD** was dissolved in 8 mL of DMSO previously degassed by bubbling with N_2_. Subsequently, a solution of CuSO_4_•5H_2_O (0.54 eq), H_2_Asc (1.62 eq), and TBTA (0.54 eq) in 2 mL of DMSO was added dropwise. The reaction mixture was heated to 80 °C with vigorous stirring under a N_2_ atmosphere for 12 h. Then, the reaction mixture was cooled and precipitated into cold acetone (200 mL) and the corresponding formed solid was filtered under vacuum. The final products were purified by SEC through Bio-Gel^®^ P-6 medium using water as an eluent. The adequate fraction was lyophilized to obtain the corresponding **EDTA-βCD** dendrimers.

##### EDTA4PhCD Dendrimer (**D**) 

The final dendrimer **D** was obtained as a beige solid (95% yield). ^1^H NMR (400 MHz, DMSO-*d*_6_, δ ppm): 10.10 (s, 4H, –NH–), 8.16 (s, 4H, H-triazole), 7.57 (d, *J* = 8.3 Hz, 8H, Hc), 6.99 (d, *J* = 8.3 Hz, 8H, Hd), 5.89–5.66 (m, 62H, OH-2,2′, OH-3,3′), 5.05 (br, 14H, He, H-1′), 4.92–4.79 (m, 31H, H-6′, H-1), 4.62–4.31 (m, 31H, OH-6, H-6’, OH-6″), 4.00 (m, 6H, H-5′), 3.74–3.49 (m, 97H, H-6, H-3,3′, H-5), 3.37–3.22 (m, 63H, H-4,4′, H-2,2′ overlapped with H_2_O), 3.15–2.92 (m, 16H, H-6″, Hb), 2.90 (s, 4H, Ha); ^13^C-DEPTQ NMR (101 MHz, DMSO-*d*_6_, δ ppm): 170.20, 155.01, 143.32, 132.97, 126.41, 121.66, 115.47, 103.00, 102.10, 84.18, 82.31, 72.56, 70.75, 61.87, 60.65, 59.80, 53.23, 51.08, 40.91; IR (ATR, cm^−1^): 3325 O–H (st), 2922 CH_2_ (st), 1025 C–O (st); MS (MALDI-TOF^+^) *m*/*z*: [M + Na]^+^ calcd for C_214_H_320_N_18_O_144_Na 5471.90, found 5471.01.

##### EDTA4BenCD Dendrimer (**E**) 

The final dendrimer **E** was obtained as a beige solid (97% yield). ^1^H NMR (400 MHz, DMSO-*d*_6_, δ ppm): 8.16 (s, 4H, H-triazole), 7.17 (d, *J* = 8.33 Hz, 8H, Hd), 6.97 (d, *J* = 8.33 Hz, 8H, He), 5.91–5.69 (m, 62H, OH-2,2′, OH-3,3′), 5.06 (br, 14H, Hf, H-1′), 4.92–4.77 (m, 31H, H-6′, H-1), 4.62–4.31 (m, 31H, Hc, H-6′, OH-6, OH-6″), 4.02 (m, 6H, H-5′), 3.73–3.54 (m, 97H, H-6, H-3,3′, H-5), 3.46–3.22 (m, 63H, H-4,4′, H-2,2′ overlapped with H_2_O), 3.12–3.10 (m, 4H, H-6″), 2.89 (m, 4H, H-6″), 2.61 (m, 4H, Ha); ^13^C-DEPTQ NMR (101 MHz, DMSO-*d*_6_, δ ppm): 171.11, 158.10, 143.27, 130.10, 129.99, 126.25, 115.28, 102.81, 102.07, 84.25, 82.86, 82.32, 81.75, 73.80, 73.14, 72.85, 72.54, 70.76, 61.70, 60.95, 60.71, 60.64, 59.74, 56.45, 52.47, 51.15, 41.57; IR (ATR, cm^−1^): 3328 O–H (st), 2925 CH_2_ (st), 1023 C–O (st); MS (MALDI-TOF^+^) *m*/*z*: [M + K]^+^ calcd for C_218_H_328_N_18_O_144_K 5545, found 5545.

##### EDTA4TyrCD Dendrimer (**F**) 

The final dendrimer **F** was obtained as a beige solid (95% yield). ^1^H NMR (400 MHz, DMSO-*d*_6_, δ ppm): 8.21 (br, 4H, –CONH–), 8.15 (s, 4H, H-triazole), 7.14 (d, *J* = 8.22 Hz, 8H, He), 6.96 (d, *J* = 8.21 Hz, 8H, Hf), 5.91–5.67 (m, 62H, OH-2,2′, OH-3,3′), 5.06 (br, 14H, Hg, H-1′), 4.92–4.79 (m, 31H, H-6′, H-1), 4.61–4.32 (m, 31H, OH-6, H-6′, OH-6″), 3.99 (m, 6H, H-5′), 3.73–3.59 (m, 97H, H-6, H-3,3′, H-5), 3.46–3.22 (m, 63H, H-4,4′, H-2,2′ overlapped with H_2_O), 3.14–3.10 (m, 13H, H-6″, Hc), 2.91 (m, 4H, H-6″), 2.68 (br, 10H, H-b,d), 2.57 (m, 15H, Ha overlapped with DMSO); ^13^C-DEPTQ NMR (101 MHz, DMSO-*d*_6_, δ ppm): 171.02, 157.40, 143.38, 132.33, 130.35, 126.36, 115.34, 102.72, 102.10, 84.21, 84.19, 82.84, 82.30, 73.80, 73.16, 72.55, 60.76, 54.66, 40.70, 33.42; IR (ATR, cm^−1^): 3306 O–H (st), 2928 CH_2_ (st), 1023 C–O (st); MS (MALDI-TOF^+^) *m*/*z*: [M + K]^+^ calcd for C_222_H_337_N_18_O_144_K 5601.20, found 5601.

### 3.3. Job Plot Method 

The Job plot method was carried out according to the previously reported methodology with some modifications [31,57]. Two stock solutions, a Solution Host (Sol. *H*) of dendrimer EDTA4BenCD (**E**) 7.3 mM in βCD cavities, and a Solution Guest (Sol. *G*) of AdCOOH 7.3 mM, were prepared in D_2_O. With these solutions, a series of nine samples in NMR tubes containing both **E** and AdCOOH with a total concentration ([AdCOOH]+[**E**]) fixed at 7.3 mM were prepared. This was accomplished by introducing increasing portions of 50 μL, up to 500 μL of Sol. *H* in the adequate NMR tube, and then in the corresponding tube, decreasing amounts were added starting from 500 μL, up to 50 μL of Sol. *G*. Thus, solutions with constant volume with varying βCD molar fractions (XCD = [**E**]/([**E**] + [AdCOOH])) in a complete range (0.1 < r < 0.9) were obtained. The NMR measurements were performed using the signal of D_2_O as the internal standard. The continuous variation of the ^1^H-NMR chemical shift change “Δδ × XCD” (Δδ taken for the adamantyl H-γ; see Figure 3 for (**E**) and S111 in SI for (**C**)) was plotted against XCD.

### 3.4. Determination of Water Solubility for EDTA di-βCD (***A**–**C***) and EDTA-βCD Dendrimers (***D**–**F***)

The determination of the water solubility of all EDTA-**β**CD platforms was carried out according to the method reported by Jozwiakowski and Connors [45] with some modifications. Excess amounts of the corresponding compound (**A**–**D**) were placed in three amber vials of 5 mL with a screw cap, which were sealed with parafilm to avoid water evaporation. They were stirred in an oil bath at a constant temperature of 25 ± 0.01 °C for 48 h. The supernatant was separated from the solid phase by filtration through a Milli-Q membrane (pore size of 0.45 μm) by injection of the mixture into disposable plastic syringes of 3 mL at 25 °C. The supernatant of each sample was placed in three different vials. The samples were lyophilized for 48 h and the obtained solid was weighted on a scale with an uncertainty of ±0.001 g.

### 3.5. DLS Measurements 

DLS particle size measurements were conducted at 20 °C using a Nano Zetasizer (Malvern Instruments Ltd., Malvern, UK) operating at 633 nm and recording the back-scattered light at an angle of 173° and 1.33 as the refractive index. The sample temperature was allowed to equilibrate for 10 min before each measurement. Dimers and dendrimer samples were prepared at a concentration of 1 mg/mL in water and filtered through a 0.02 μm filter. The light scattering was recorded for 200 s, with a minimum of twelve measurements taken per sample. Hydrodynamic size is reported as the intensity-weighted average.

### 3.6. Biological Procedures

Cell culture. The human cancer cell lines MCF-7 and MDA-MB-231 (breast), HeLa (cervical adenocarcinoma), and SK-LU-1 (lung adenocarcinoma) were selected for this study and were purchased from the ATCC^®^ (Manassas, VA, USA). Cell lines were grown in DMEM with 10 mM of non-essential amino acids, supplemented with 10% heat-inactivated fetal bovine serum (BioWest, Riverside, MO, USA). These four adherent epithelial cell lines were maintained in proliferation in a humidified atmosphere containing 5% CO_2_ at 37 °C.

Viability studies. MTT assay was carried out in order to determine the cytotoxic activity of the EDTA di-βCD (**A**–**C**) and EDTA-βCD dendrimers (**D**–**F**) in the selected cell lines. Briefly, the cell lines were seeded in a 96-well plate at a density of 6.5 × 10^3^ cells per well in 200 µL of medium. After 24 h of incubation, the cells formed a monolayer, and 50 µL of the medium with different concentrations of the dendrimer were added. Cell viability was determined after 48 h exposure to the dendrimer molecules by adding 20 µL of 2.5 mg/mL 3-(4,5-dimethylthiazol-2-yl)-2,5-diphenyl tetrazoliumbromide (MTT). After an incubation period of 2 h at 37 °C, the supernatant was removed, and insoluble formazan-formed crystals were dissolved by adding 200 µL of DMSO. The absorbance of each well was measured at 570 nm using an Epoch microplate spectrophotometer (Biotek^®^, Winooski, VT, USA). Absorbance is directly related to the percentage of cell viability (%), and control cells without treatment were considered at 100% of viability.

## 4. Conclusions

In this work, we synthesized a highly water-soluble family of platforms based on an EDTA core and βCD moieties in the periphery using click chemistry as an assembling strategy for the surface modification of this type of molecule. Furthermore, we demonstrated that despite the occurrence of the self-inclusion process in water presented by all of the synthesized platforms, it was possible to form [1:2] and [1:4] ICs with AdCOOH in that medium, resulting in the full host ability of the βCD units in water for dimers and dendrimers, respectively. We assert that with any selected spacer in the new EDTA-βCD dimers or EDTA-βCD dendrimers, the physicochemical properties and their inclusion capacity are conserved and are equivalent to those of a classical PAMAM dendrimer. Finally, we are sure that the field of applications for this new family of platforms based on EDTA-βCD could be greatly broadened and their potential use as drug nanocarriers could be also exploited.

## Figures and Tables

**Table 1:** Water solubility results for the platforms based on EDTA-βCD **A**–**F** ^1^.

Compound	Solubility (g/mL)	Compound	Solubility (g/mL)
EDTA2PhCD (**A**)	1.582 ± 0.002	EDTA4PhCD (**D**)	1.594 ± 0.003
EDTA2BenCD (**B**)	1.594 ± 0.001	EDTA4BenCD (**E**)	1.601 ± 0.002
EDTA2TyrCD (**C**)	1.591 ± 0.003	EDTA4TyrCD (**F**)	1.623 ± 0.002

^1^ Data represent mean ± SD (n = 3).

## Data Availability

Not applicable.

## Supplementary Materials

The following supporting information can be downloaded at: https://www.mdpi.com/article/10.3390/ijms241914422/s1: IR, NMR, and MS spectra of the intermediate and final compounds.
