# Supplementary material for: Exploring the Influence of Spacers in EDTA–β-Cyclodextrin Dendrimers: Physicochemical Properties and In Vitro Biological Behavior
Source: Int J Mol Sci. 2023 Sep 22;24(19):14422. doi: 10.3390/ijms241914422 (PMC10572662; doi:10.3390/ijms241914422)
Supplement: Supplementary file 1 [file ijms-24-14422-s001.zip › ijms-2610362-supplementary.pdf]

Exploring the Influence of Spacers in EDTA-βCD Dendrimers: Physicochemical Properties and In Vitro Biological Behavior

Israel González-Méndez [1,2], Kendra Sorroza-Martínez [3], Ignacio González-Sánchez [4], Jesús Gracia-Mora [1], María Josefa Bernad-Bernad [5], Marco Cerbón [4], Ernesto Rivera [3,*] and Anatoly K. Yatsimirsky [1,*]

Supplementary Information

Analysis data of

Figure S1. ^{1}H-NMR spectrum of *tert*-butyl(4-hydroxyphenyl)carbamate in DMSO-d_6. p6

Figure S2. ^{13}C-NMR spectrum of *tert*-butyl(4-hydroxyphenyl)carbamate in DMSO-d_6. p6

Figure S3. IR spectrum of *tert*-butyl(4-hydroxyphenyl)carbamate. p7

Figure S4. DART of *tert*-butyl(4-hydroxyphenyl)carbamate. p7

Figure S5. ^{1}H-NMR spectrum of *tert*-butyl(4-(prop-2-yn-1-yloxy)phenyl)carbamate in DMSO-d_6. p8

Figure S6. ^{13}C-NMR spectrum of *tert*-butyl(4-(prop-2-yn-1-yloxy)phenyl)carbamate in DMSO-d_6. p8

Figure S7. IR spectrum of *tert*-butyl(4-(prop-2-yn-1-yloxy)phenyl)carbamate. p9

Figure S8. DART of *tert*-butyl(4-(prop-2-yn-1-yloxy)phenyl)carbamate. p9

Figure S9. ^{1}H-NMR spectrum of 4-(prop-2-yn-1-yloxy)aniline in DMSO-d_6. p10

Figure S10. ^{13}C-NMR spectrum of 4-(prop-2-yn-1-yloxy)aniline in DMSO-d_6. p10

Figure S11. IR spectrum of 4-(prop-2-yn-1-yloxy)aniline. p11

Figure S12. DART of 4-(prop-2-yn-1-yloxy)aniline. p11

Figure S13. ^{1}H-NMR spectrum of *tert*-butyl(4-hydroxybenzyl)carbamate in DMSO-d_6. p12

Figure S14. ^{13}C-NMR spectrum of *tert*-butyl(4-hydroxybenzyl)carbamate in DMSO-d_6. p12

Figure S15. IR spectrum of *tert*-butyl(4-hydroxybenzyl)carbamate. p13

Figure S16. DART of *tert*-butyl(4-hydroxybenzyl)carbamate. p13

Figure S17. ^{1}H-NMR spectrum of *tert*-butyl (4-(prop-2-yn-1-yloxy)benzyl)carbamate in DMSO-d_6. p14

Figure S18. ^{13}C-NMR spectrum of *tert*-butyl(4-(prop-2-yn-1-yloxy)benzyl)carbamate in DMSO-d_6. p14

Figure S19. IR spectrum of *tert*-butyl(4-(prop-2-yn-1-yloxy)benzyl)carbamate. p15

Figure S20. DART of *tert*-butyl(4-(prop-2-yn-1-yloxy)benzyl)carbamate. p15

Figure S21. ^{1}H-NMR spectrum of [4-(prop-2-yn-1-yloxy)phenyl]methanamine in DMSO-d_6. p16

Figure S22. ^{13}C-NMR spectrum of [4-(prop-2-yn-1-yloxy)phenyl]methanamine in DMSO-d_6. p16

Figure S23. IR spectrum of [4-(prop-2-yn-1-yloxy)phenyl]methanamine. p17

Figure S24. DART of [4-(prop-2-yn-1-yloxy)phenyl]methanamine. p17

Figure S25. ^{1}H-NMR spectrum of *tert*-butyl (4-hydroxyphenethyl)carbamate in DMSO-d_6. p18

Figure S26. ^{13}C-NMR spectrum of *tert*-butyl (4-hydroxyphenethyl)carbamate in DMSO-d_6. p18

Figure S27. IR spectrum of *tert*-butyl (4-hydroxyphenethyl)carbamate. p19

Figure S28. DART *tert*-butyl (4-hydroxyphenethyl)carbamate. p19

Figure S29. ^{1}H-NMR spectrum of *tert*-butyl (4-(prop-2-yn-1-yloxy)phenetyl)carbamate in DMSO-d_6. p20

Figure S30. ^{13}C-NMR spectrum of *tert*-butyl (4-(prop-2-yn-1-yloxy)phenetyl)carbamate in DMSO-d_6. p20

Figure S31. IR spectrum of *tert*-butyl (4-(prop-2-yn-1-yloxy)phenetyl)carbamate. p21

Figure S32. DART spectrum of *tert*-butyl (4-(prop-2-yn-1-yloxy)phenetyl)carbamate. p21

Figure S33. ^{1}H-NMR spectrum of 2-(4-(prop-2-yn-1-yloxy)phenyl)ethan-1-amine in DMSO-d_6. p22

Figure S34. ^{13}C-NMR spectrum of 2-(4-(prop-2-yn-1-yloxy)phenyl)ethan-1-amine in DMSO-d_6. p22

Figure S35. IR spectrum of 2-(4-(prop-2-yn-1-yloxy)phenyl)ethan-1-amine. p23

Figure S36. DART spectrum of 2-(4-(prop-2-yn-1-yloxy)phenyl)ethan-1-amine. p23

Figure S37. ^{1}H-NMR spectrum of disubstituted EDTA alkyne **13** in DMSO-d_6. p24

Figure S38. ^{13}C-NMR spectrum of disubstituted EDTA alkyne **13** in DMSO-d_6. p24

Figure S39. IR spectrum of disubstituted EDTA alkyne **13**. p25

Figure S40. DART spectrum of disubstituted EDTA alkyne **13**. p25

Figure S41. ^{1}H-NMR spectrum of disubstituted EDTA alkyne **14** in DMSO-d_6. p26

Figure S42. ^{13}C-NMR spectrum of disubstituted EDTA alkyne **14** in DMSO-d_6. p26

Figure S43. IR spectrum of disubstituted EDTA alkyne **14**. p27

Figure S44. DART spectrum of disubstituted EDTA alkyne **14**. p27

Figure S45. ^{1}H-NMR spectrum of disubstituted EDTA alkyne **15** in DMSO-d_6. p28

Figure S46. ^{13}C-NMR spectrum of disubstituted EDTA alkyne **15** in DMSO-d_6. p28

Figure S47. IR spectrum of disubstituted EDTA alkyne **15**. p29

Figure S48. ESI spectrum of disubstituted EDTA alkyne **15**. p29

Figure S49. ^{1}H-NMR spectrum of EDTA G0-alkyne **16** in DMSO-d_6. p30

Figure S50. ^{13}C-NMR spectrum of EDTA G0-alkyne **16** in DMSO-d_6. p30

Figure S51. IR spectrum of EDTA G0-alkyne **16**. p31

Figure S52. ESI spectrum of disubstituted EDTA alkyne **16**. p31

Figure S53. ^{1}H-NMR spectrum of EDTA G0-alkyne **17** in DMSO-d_6. p32

Figure S54. ^{13}C-NMR spectrum of EDTA G0-alkyne **17** in DMSO-d_6. p32

Figure S55. 2D NMR HMQC spectrum of EDTA G0-alkyne **17** in DMSO-d_6. p33

Figure S56. 2D NMR COSY spectrum of EDTA G0-alkyne **17** in DMSO-d_6. p33

Figure S57. IR spectrum of EDTA G0-alkyne **17**. p34

Figure S58. ESI spectrum of disubstituted EDTA alkyne **17.** p34

Figure S59. ^{1}H-NMR spectrum of tetrasubstituted EDTA G0-alkyne **18** in DMSO-d_6. p35

Figure S60. ^{13}C-NMR spectrum of tetrasubstituted EDTA G0-alkyne **18** in DMSO-d_6. p35

Figure S61. IR spectrum of tetrasubstituted EDTA G0-alkyne **18** p36

Figure S62. ESI spectrum of tetrasubstituted EDTA G0-alkyne **18**. p36

Figure S63. ^{1}H-NMR spectrum of dendritic EDTA2PhCD (**A**) in DMSO-d_6. p37

Figure S64. ^{13}C-NMR spectrum of dendritic EDTA2PhCD (**A**) in DMSO-d_6. p37

Figure S65. 2D NMR HMQC spectrum of dendritic EDTA2PhCD (**A**) in DMSO-d_6. p38

Figure S66. 2D NMR COSY spectrum of dendritic EDTA2PhCD (**A**) in DMSO-d_6. p38

Figure S67. IR spectrum of dendritic EDTA2PhCD (**A**). p39

Figure S68. MALDI-TOF spectrum of dendritic EDTA2PhCD (**A**). p39

Figure S69. ^{1}H-NMR spectrum of dendritic EDTA2PhCD (**A**) in D_2O. p40

Figure S70. 2D NMR NOESY spectrum of EDTA2PhCD (**A**) in D_2O. p40

Figure S71. 2D NMR HMQC spectrum of EDTA2PhCD (**A**) in D_2O. p41

Figure S72. ^{1}H-NMR spectrum of dendritic EDTA2BenCD (**B**) in DMSO-d_6. p41

Figure S73. ^{13}C-NMR spectrum of dendritic EDTA2BenCD (**B**) in DMSO-d_6. p42

Figure S74. 2D NMR HMQC spectrum of EDTA2BenCD (**B**) in DMSO-d_6. p42

Figure S75. 2D NMR COSY spectrum of EDTA2BenCD (**B**) in DMSO-d_6. p43

Figure S76. IR spectrum of dendritic EDTA2BenCD (**B**). p43

Figure S77. MALDI-TOF spectrum of dendritic EDTA2BenCD (**B**). p44

Figure S78. ^{1}H-NMR spectrum of dendritic EDTA2BenCD (**B**) in D_2O. p44

Figure S79. 2D NMR NOESY spectrum of EDTA2BenCD (**B**) in D_2O. p45

Figure S80. ^{1}H-NMR spectrum of dendritic EDTA2TyrCD (**C**) in DMSO-d_6. p45

Figure S81. ^{13}C-NMR spectrum of dendritic EDTA2TyrCD (**C**) in DMSO-d_6. p46

Figure S82. 2D NMR HMQC spectrum of EDTA2TyrCD (**C**) in DMSO-d_6. p46

Figure S83. 2D NMR COSY spectrum of dendritic EDTA2TyrCD (**C**) in DMSO-d_6. p47

Figure S84. IR spectrum of dendritic EDTA2TyrCD (**C**). p47

Figure S85. ESI-TOF spectrum of dendritic EDTA2TyrCD (**C**). p48

Figure S86. ^{1}H-NMR spectrum of dendritic EDTA2TyrCD (**C**) in D_2O. p48

Figure S87. 2D NMR NOESY spectrum of dendritic EDTA2TyrCD (**C**) in D_2O. p49

Figure S88. ^{1}H-NMR spectrum of EDTA4PhCD dendrimer (**D**) in DMSO-d_6. p49

Figure S89. ^{13}C-NMR spectrum of EDTA4PhCD dendrimer (**D**) in DMSO-d_6. p50

Figure S90. 2D NMR HMQC spectrum of EDTA4PhCD dendrimer (**D**) in DMSO-d_6. p50

Figure S91. 2D NMR COSY spectrum of EDTA4PhCD dendrimer (**D**) in DMSO-d_6. p51

Figure S92. IR spectrum of EDTA4PhCD dendrimer (**D**). p51

Figure S93. MALDI-TOF spectrum of EDTA4PhCD dendrimer (**D**). p52

Figure S94. ^{1}H-NMR spectrum of EDTA4PhCD dendrimer (**D**) in D_2O. p52

Figure S95. 2D NMR NOESY spectrum of EDTA4PhCD dendrimer (**D**) in D_2O. p53

Figure S96. ^{13}C-NMR spectrum of EDTA4BenCD dendrimer (**E**) in DMSO-d_6. p53

Figure S97. 2D NMR HMQC spectrum of EDTA4BenCD dendrimer (**E**) in DMSO-d_6. p54

Figure S97. 2D NMR HMQC spectrum of EDTA4BenCD dendrimer (**E**) in DMSO-d_6. p54

Figure S99. IR spectrum of EDTA4BenCD dendrimer (**E**). p55

Figure S100. MALDI-TOF spectrum of EDTA4BenCD dendrimer (**E**). p55

Figure S101. 2D NMR NOESY spectrum of EDTA4BenCD dendrimer (**E**) in D_2O. p56

Figure S102. ^{1}H-NMR spectrum of EDTA4TyrCD dendrimer (**F**) in DMSO-d_6. p56

Figure S103. ^{13}C-NMR spectrum of EDTA4TyrCD dendrimer (**F**) in DMSO-d_6. p57

Figure S104. 2D NMR COSY spectrum of EDTA4TyrCD dendrimer (**F**) in DMSO-d_6. p57

Figure S105. IR spectrum of EDTA4TyrCD dendrimer (**F**). p58

Figure S106. MALDI-TOF spectrum of EDTA4TyrCD dendrimer (**F**). p58

Figure S107. ^{1}H-NMR spectrum of EDTA4TyrCD dendrimer (**F**) in D_2O. p59

Figure S108. 2D NMR NOESY spectrum of EDTA4TyrCD dendrimer (**F**) in D_2O. p59

Figure S109. Possible conformations adopted for dimer EDTA2TyrCD (**C**), with 0 to 2 reversed cavities. p60

Figure S110. Amplification of aliphatic zones in 2D NMR NOESY spectrum of IC EDTA2TyrCD (**C**) with AdCOOH in D_2O. p60

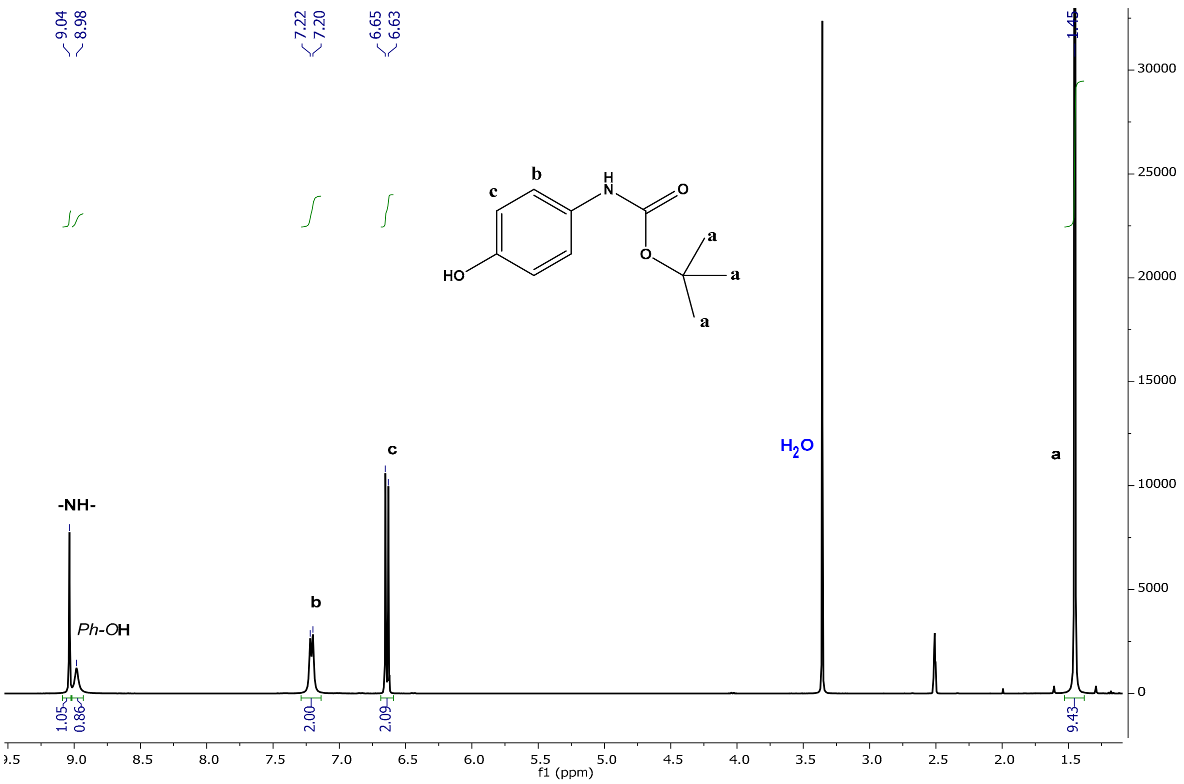

Figure S1. ^{1}H-NMR spectrum of *tert*-butyl(4-hydroxyphenyl)carbamate in DMSO-d_6.

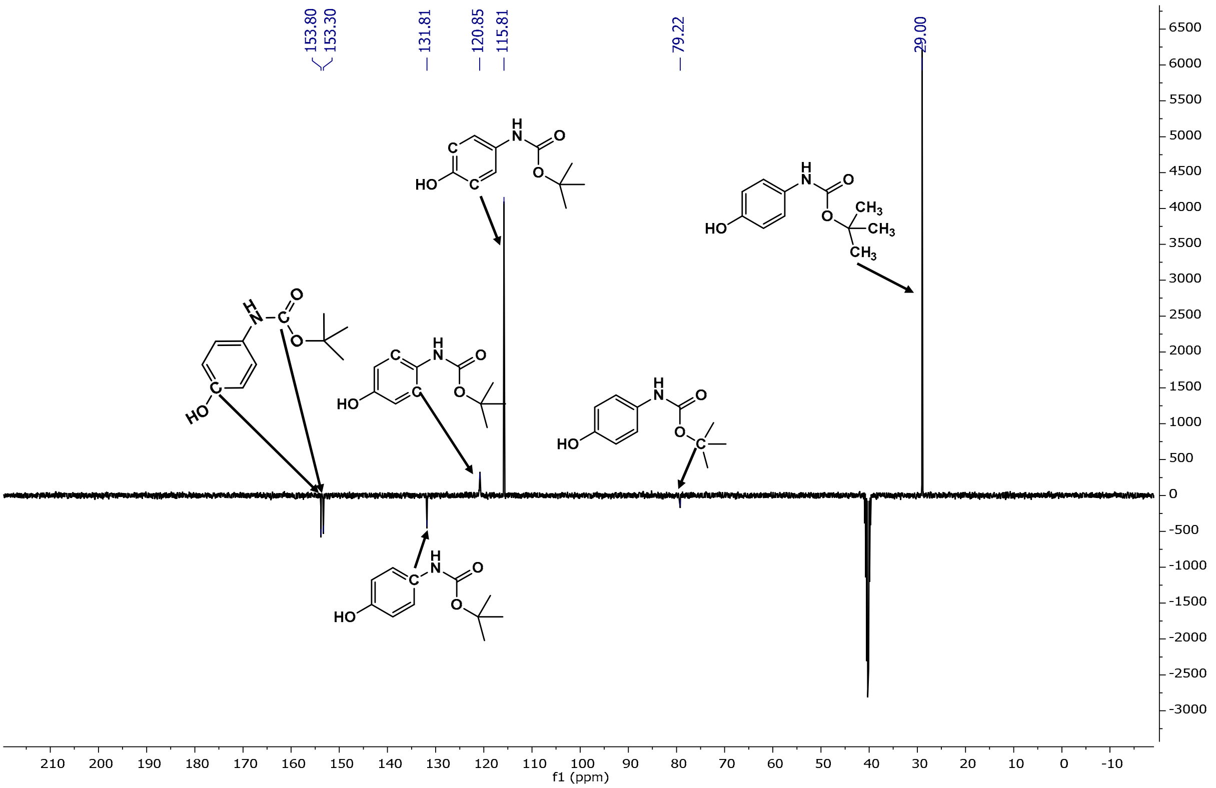

Figure S2. ^{13}C-NMR spectrum of *tert*-butyl(4-hydroxyphenyl)carbamate in DMSO-d_6.

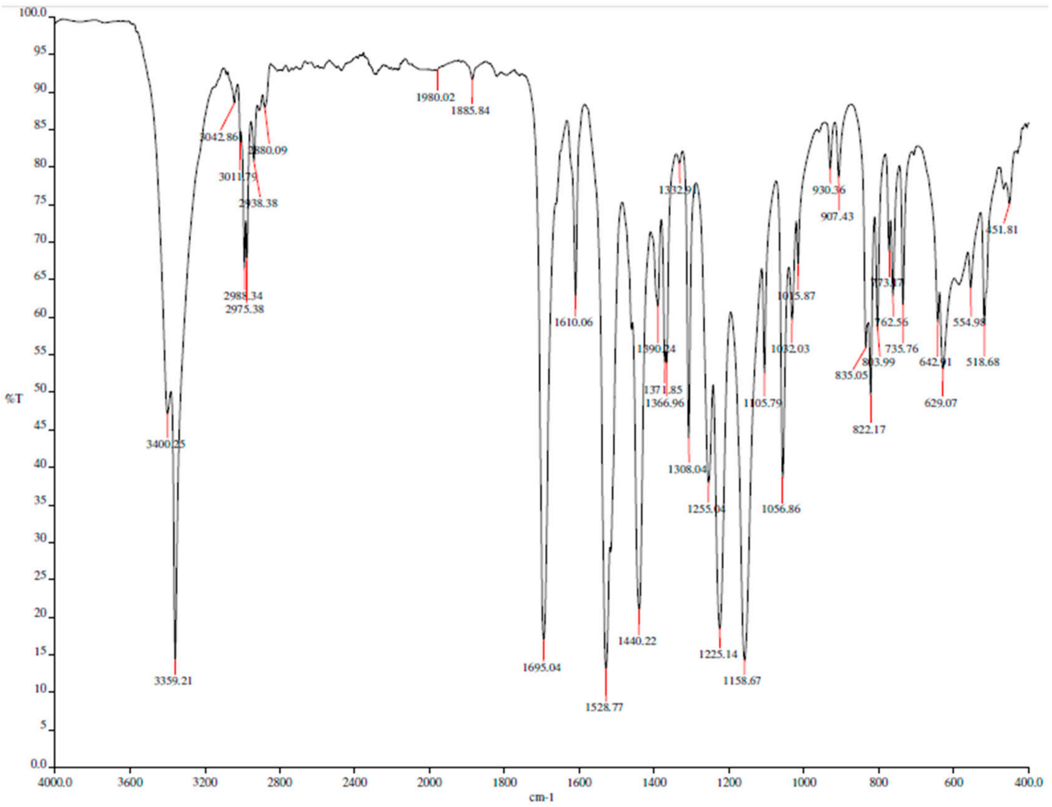

Figure S3. IR spectrum of *tert*-butyl(4-hydroxyphenyl)carbamate.

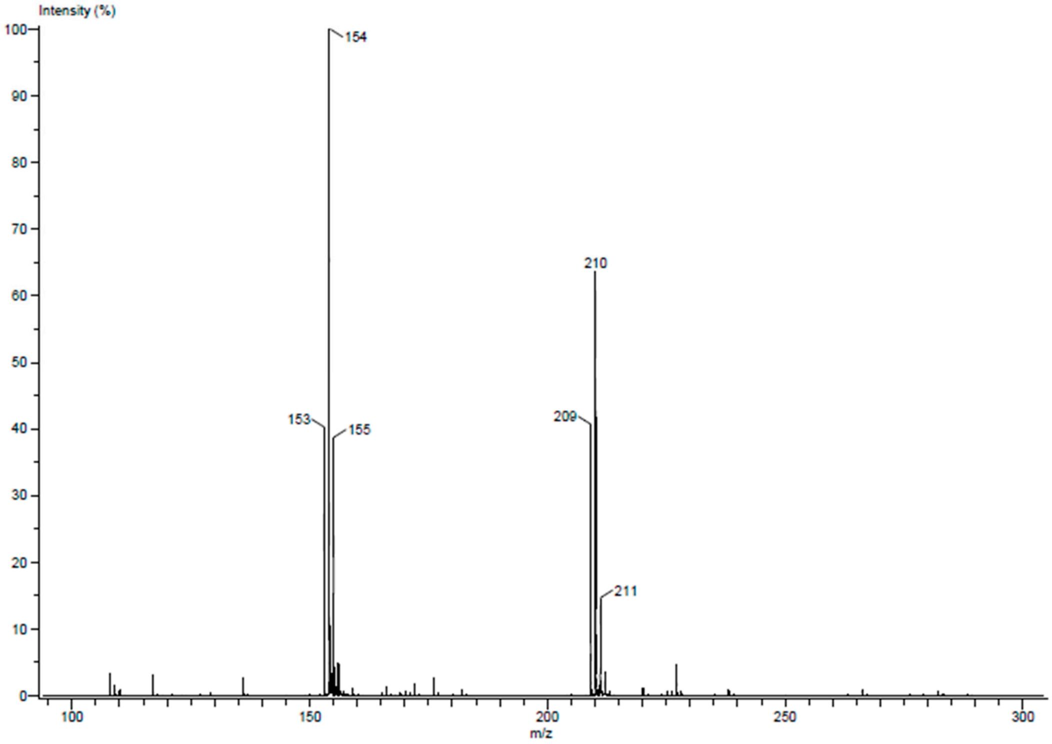

Figure S4. DART of *tert*-butyl(4-hydroxyphenyl)carbamate.

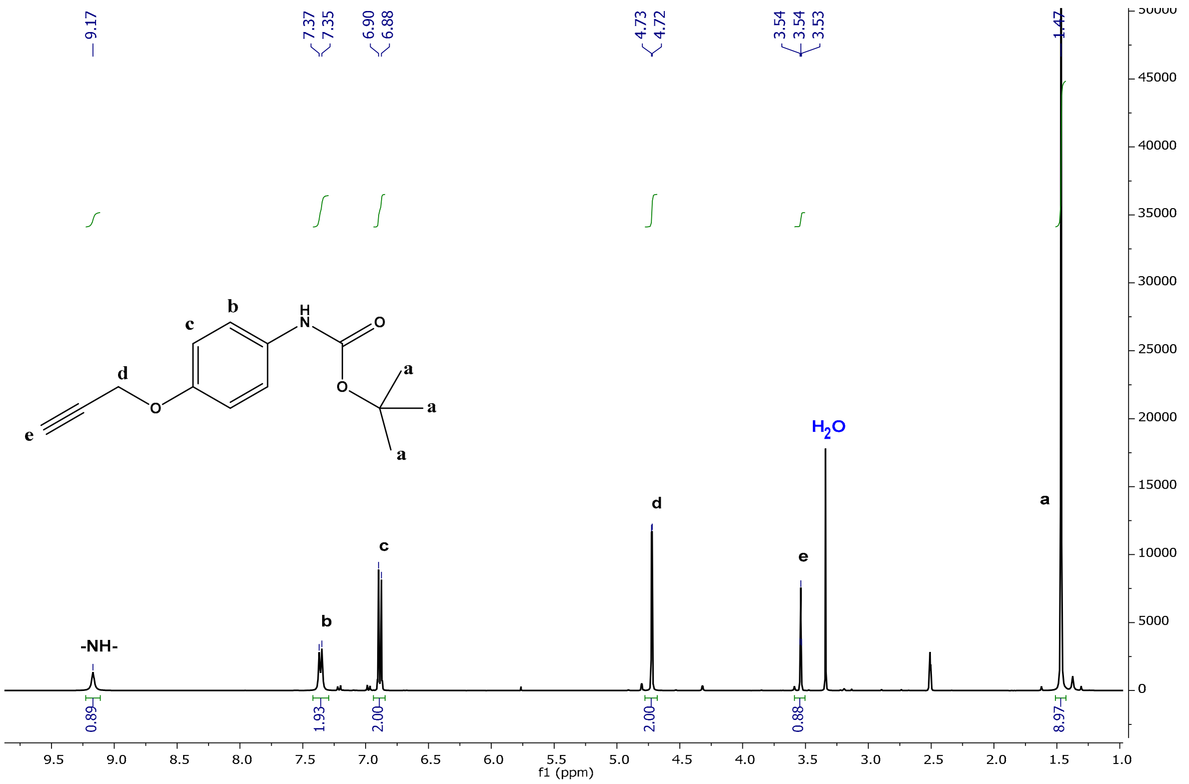

Figure S5. ^{1}H-NMR spectrum of *tert*-butyl(4-(prop-2-yn-1-yloxy)phenyl)carbamate in DMSO-d_6.

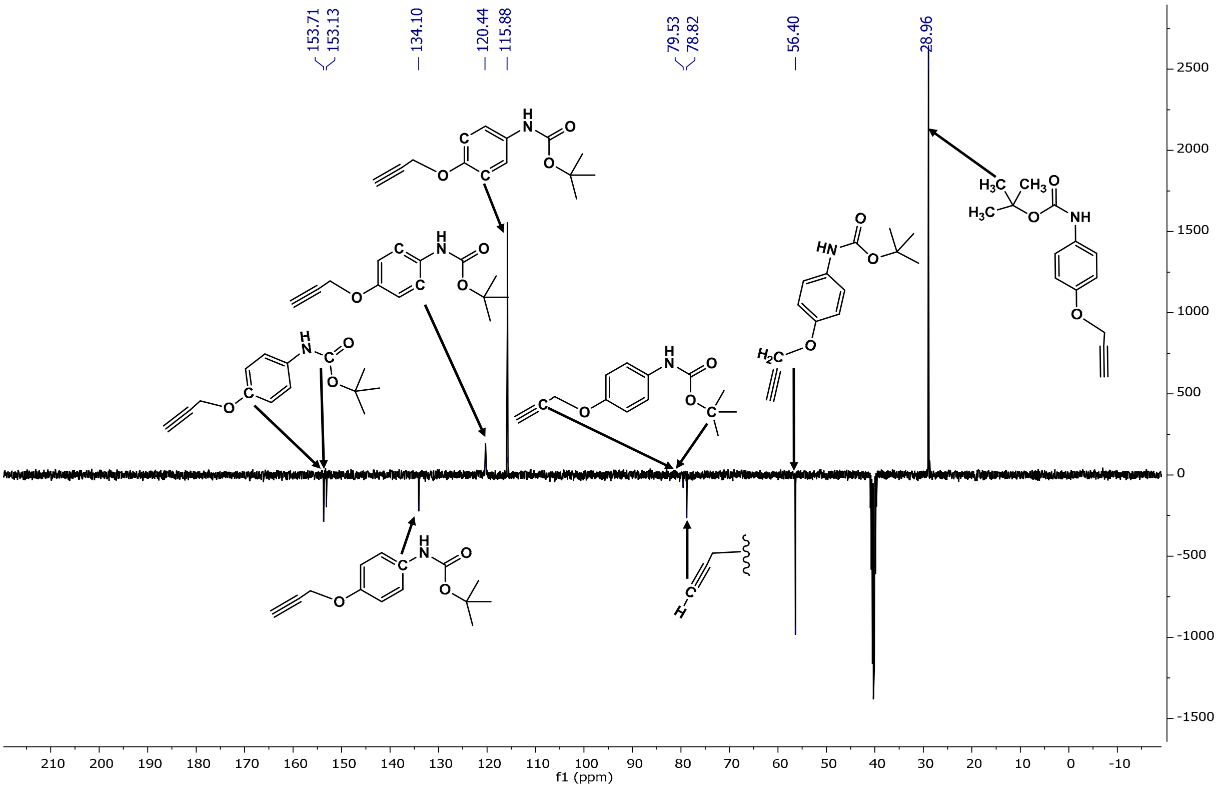

Figure S6. ^{13}C-NMR spectrum of *tert*-butyl(4-(prop-2-yn-1-yloxy)phenyl)carbamate in DMSO-d_6.

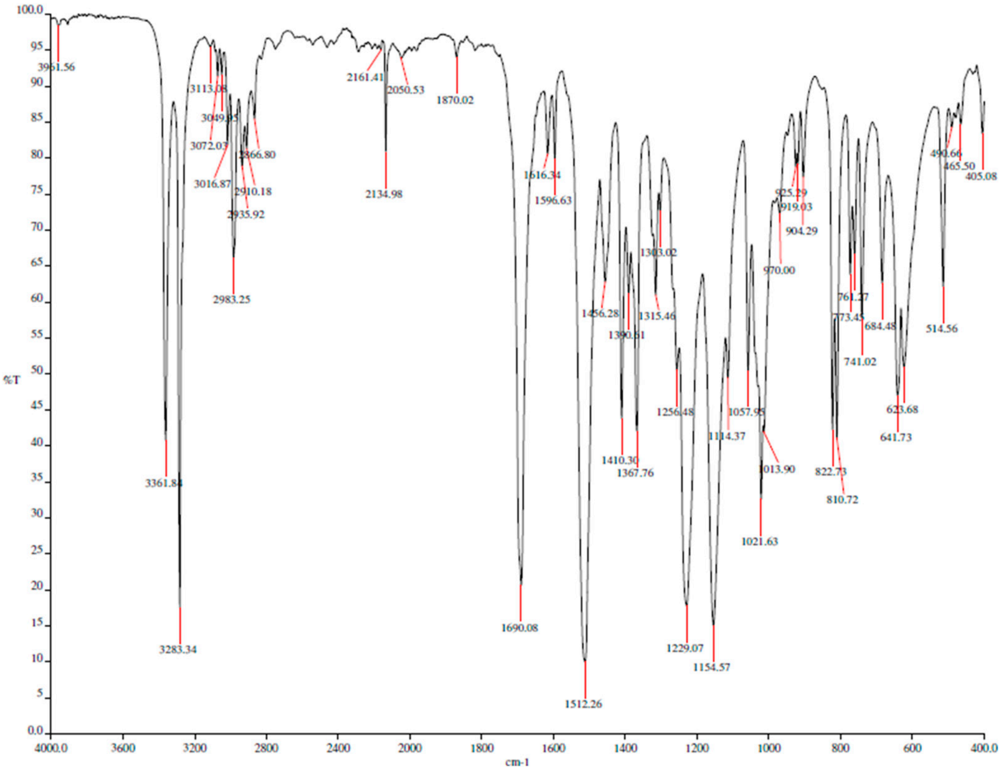

Figure S7. IR spectrum of *tert*-butyl(4-(prop-2-yn-1-yloxy)phenyl)carbamate.

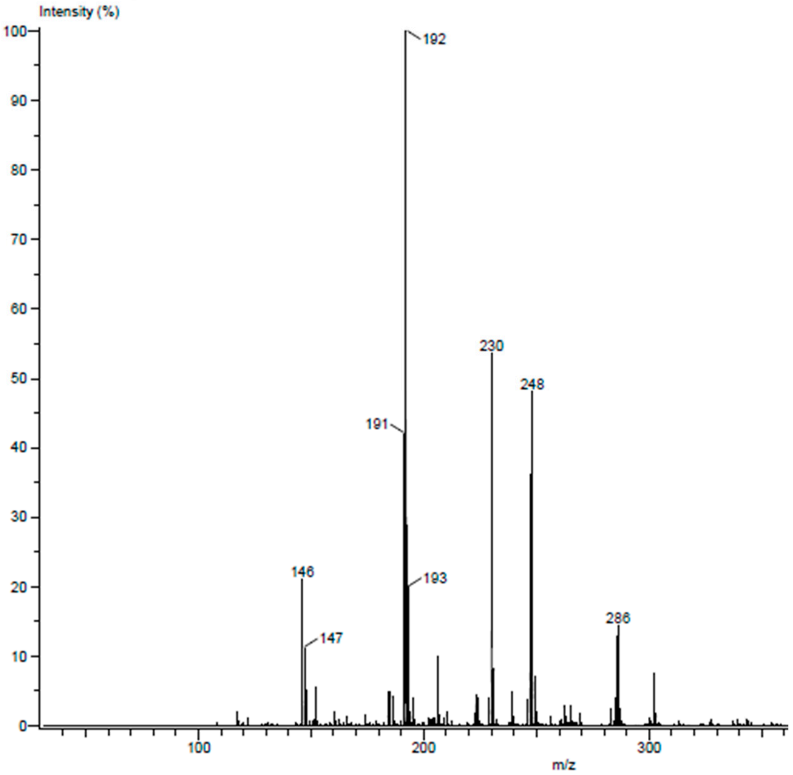

Figure S8. DART of *tert*-butyl(4-(prop-2-yn-1-yloxy)phenyl)carbamate.

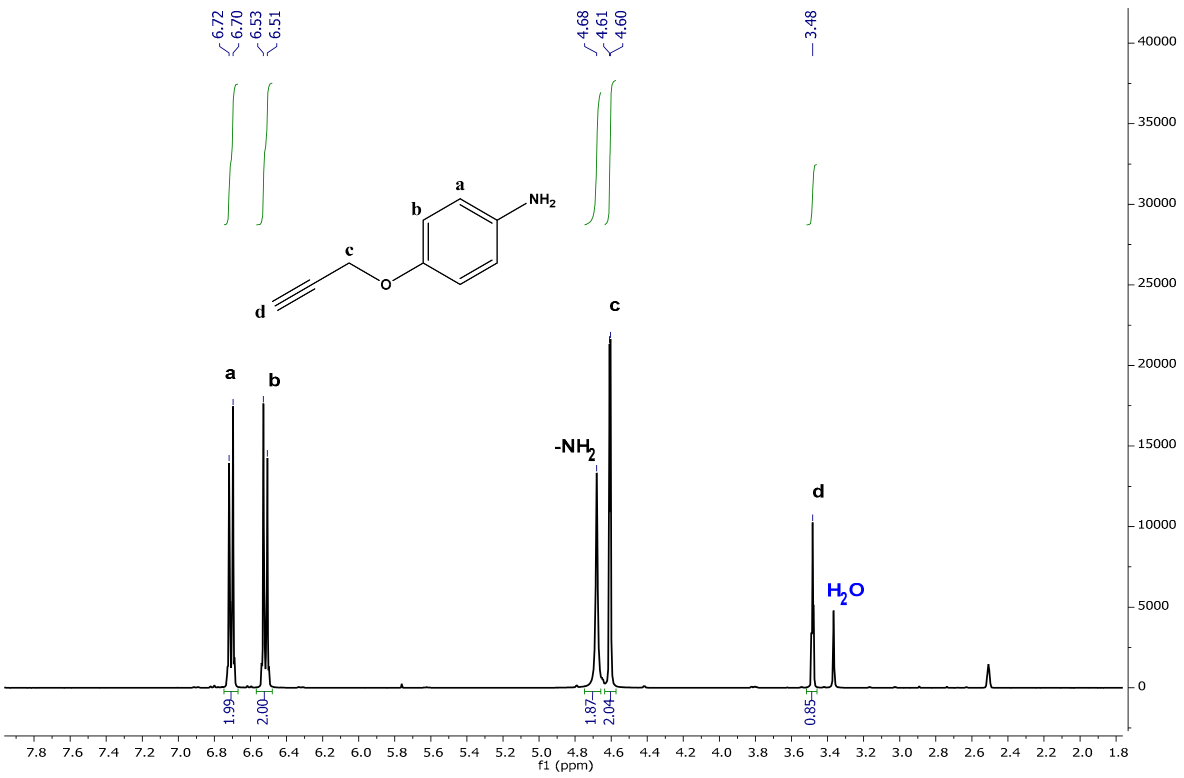

Figure S9. ^{1}H-NMR spectrum of 4-(prop-2-yn-1-yloxy)aniline in DMSO-d_6.

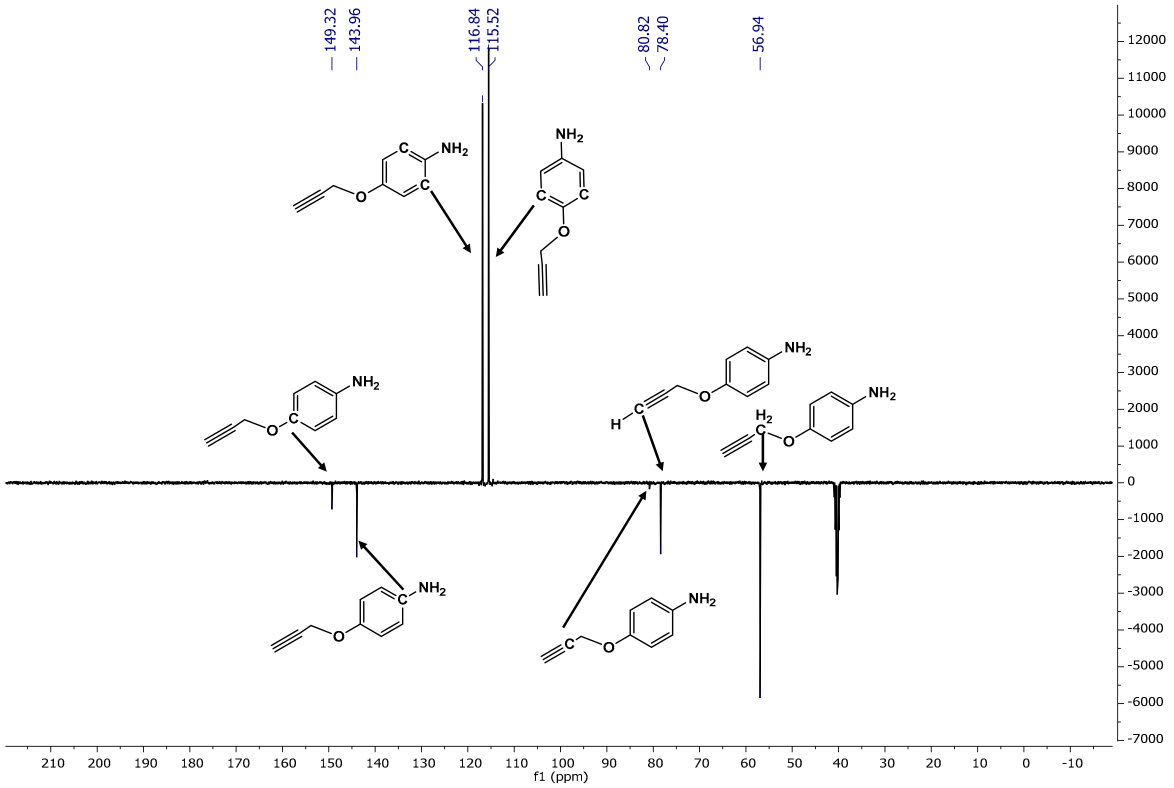

Figure S10. ^{13}C-NMR spectrum of 4-(prop-2-yn-1-yloxy)aniline in DMSO-d_6.

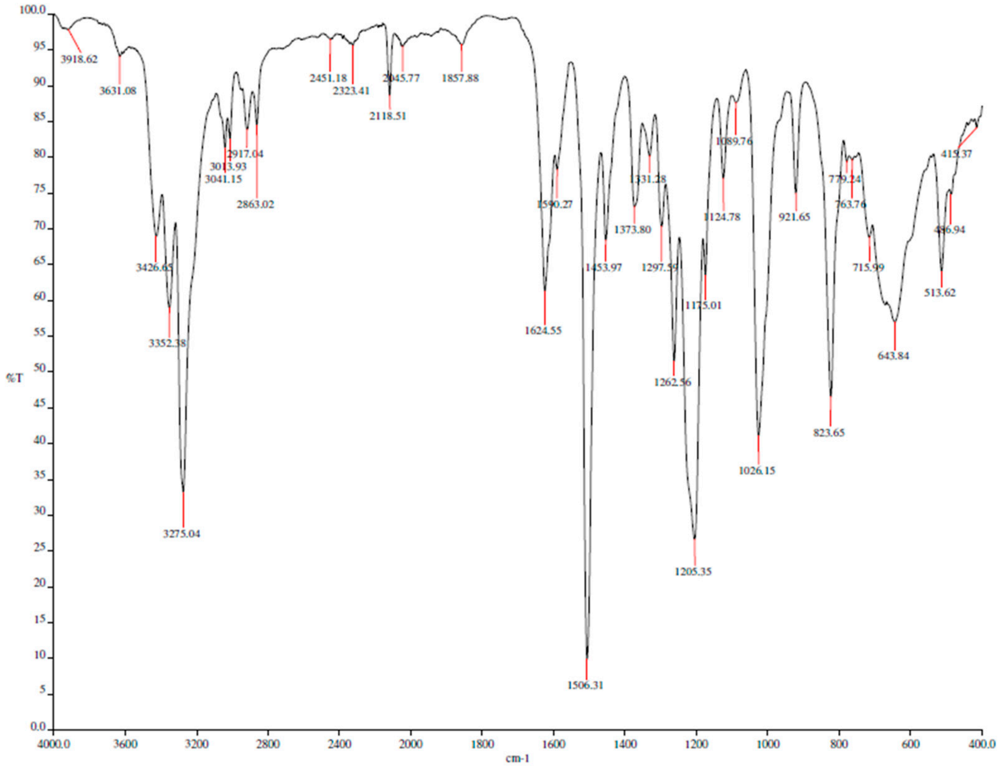

Figure S11. IR spectrum of 4-(prop-2-yn-1-yloxy)aniline.

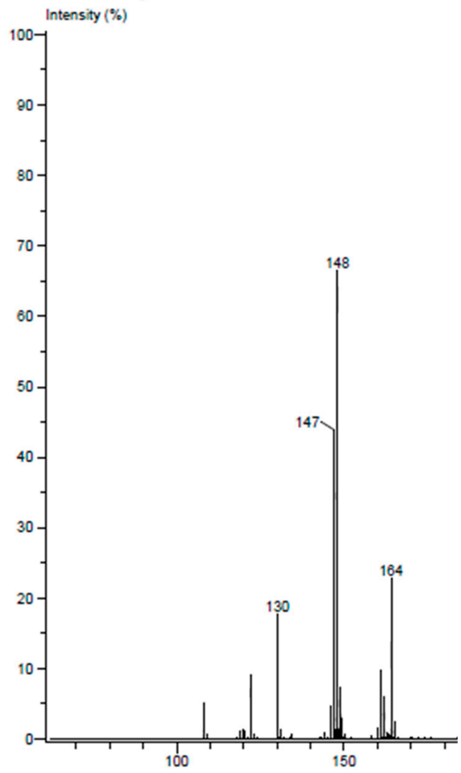

Figure S12. DART of 4-(prop-2-yn-1-yloxy)aniline.

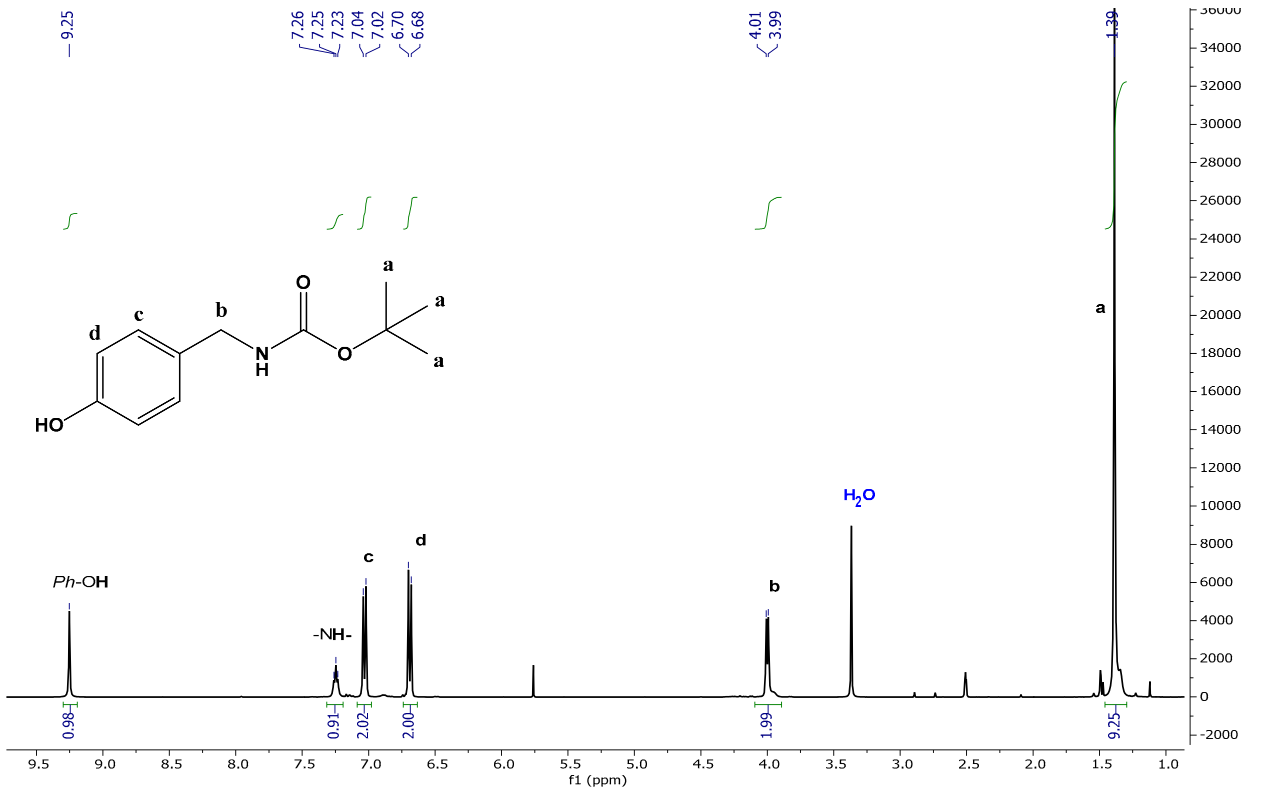

Figure S13. ^{1}H-NMR spectrum of *tert*-butyl(4-hydroxybenzyl)carbamate in DMSO-d_6.

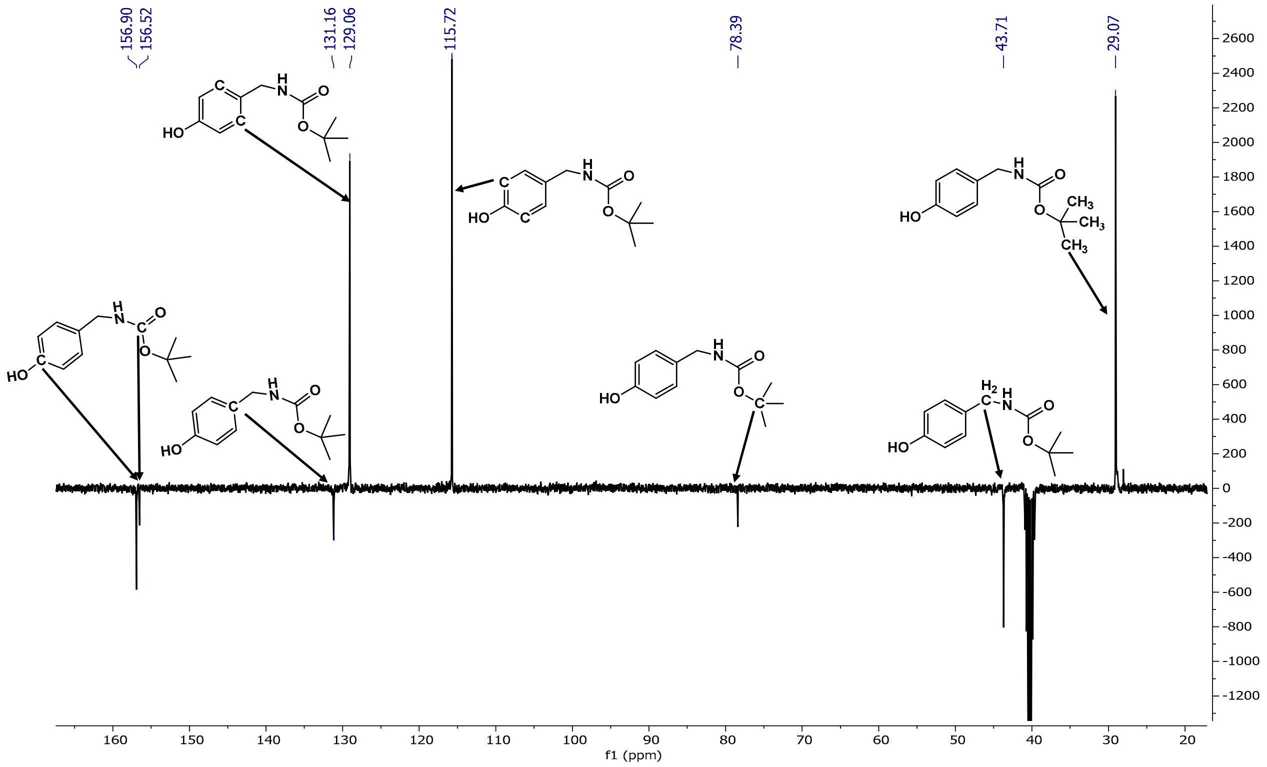

Figure S14. ^{13}C-NMR spectrum of *tert*-butyl(4-hydroxybenzyl)carbamate in DMSO-d_6.

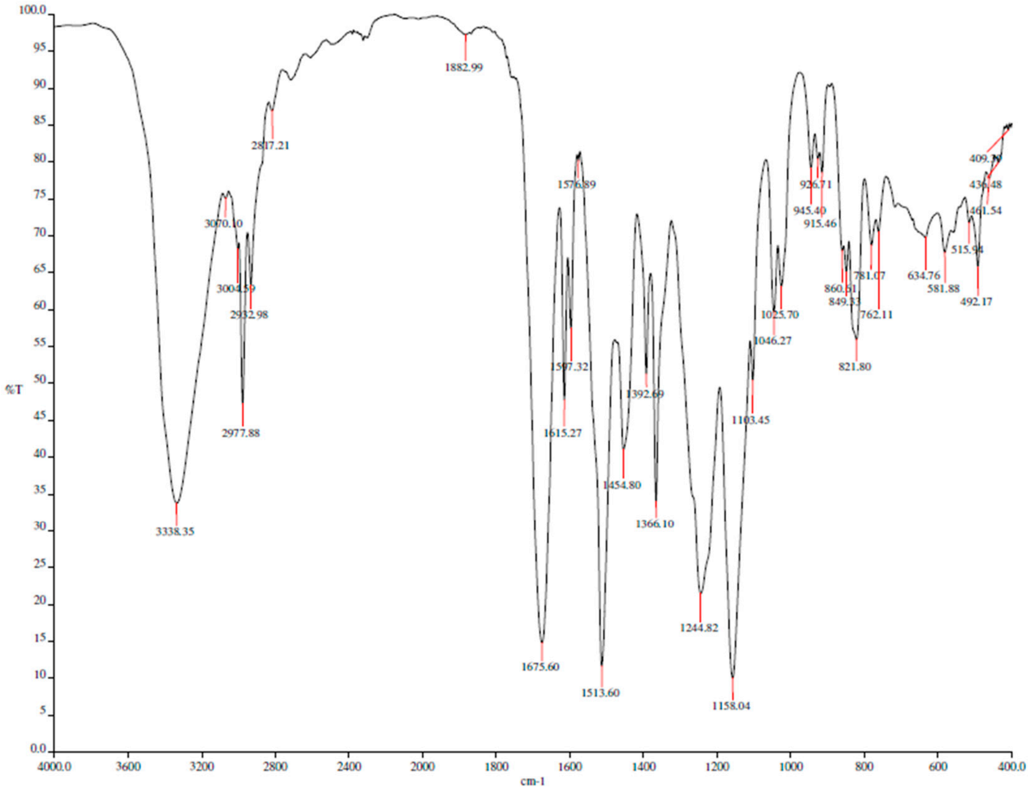

Figure S15. IR spectrum of *tert*-butyl(4-hydroxybenzyl)carbamate.

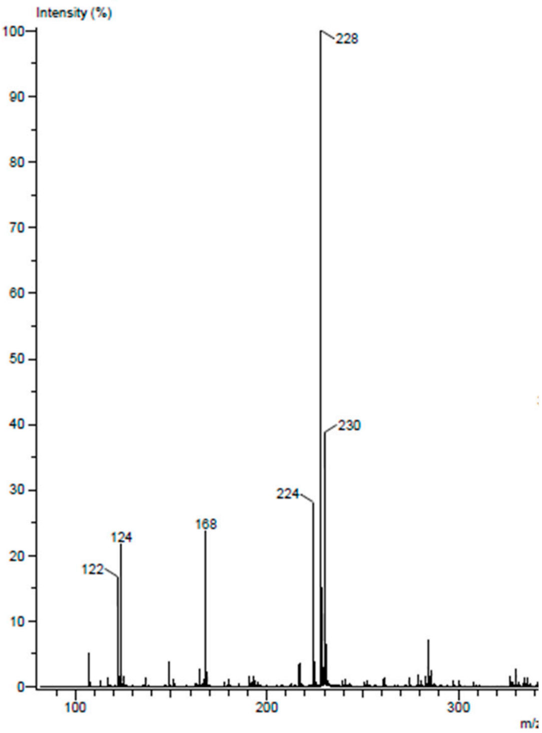

Figure S16. DART of *tert*-butyl(4-hydroxybenzyl)carbamate.

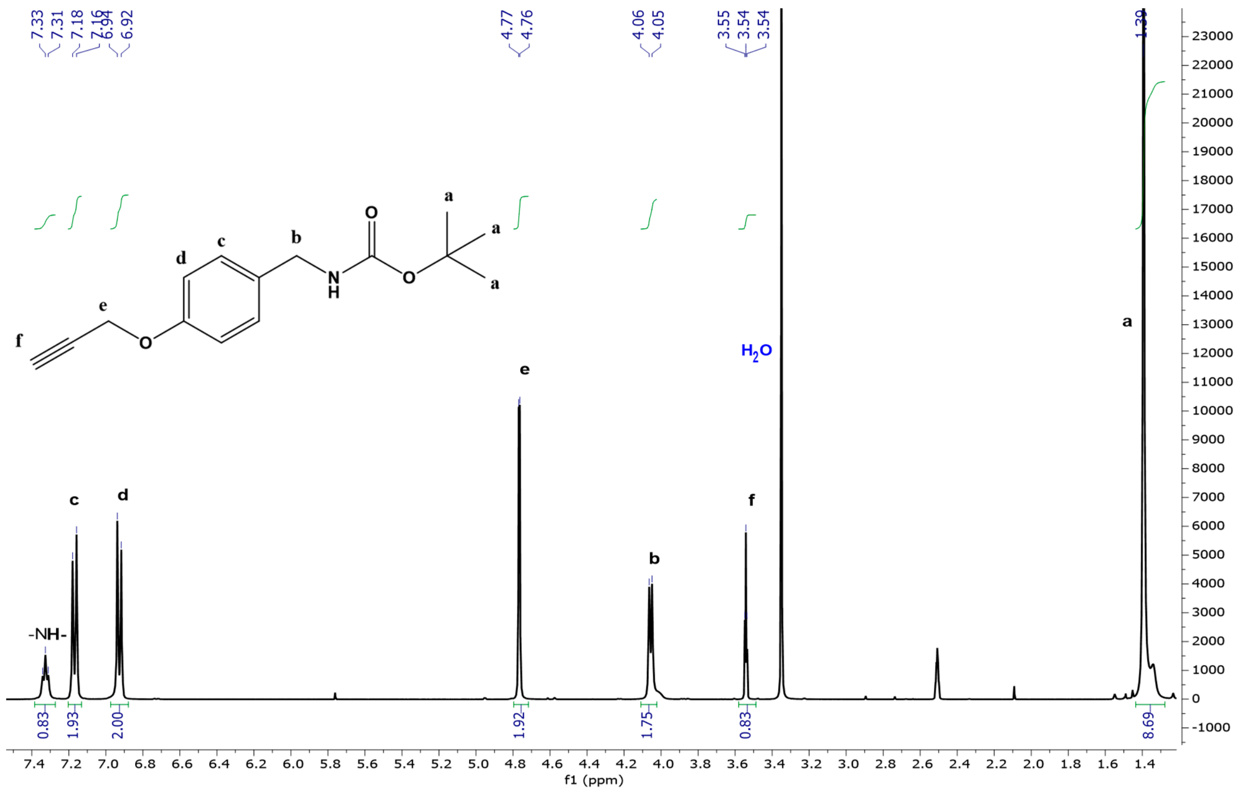

Figure S17. ^{1}H-NMR spectrum of *tert*-butyl (4-(prop-2-yn-1-yloxy)benzyl)carbamate in DMSO-d_6.

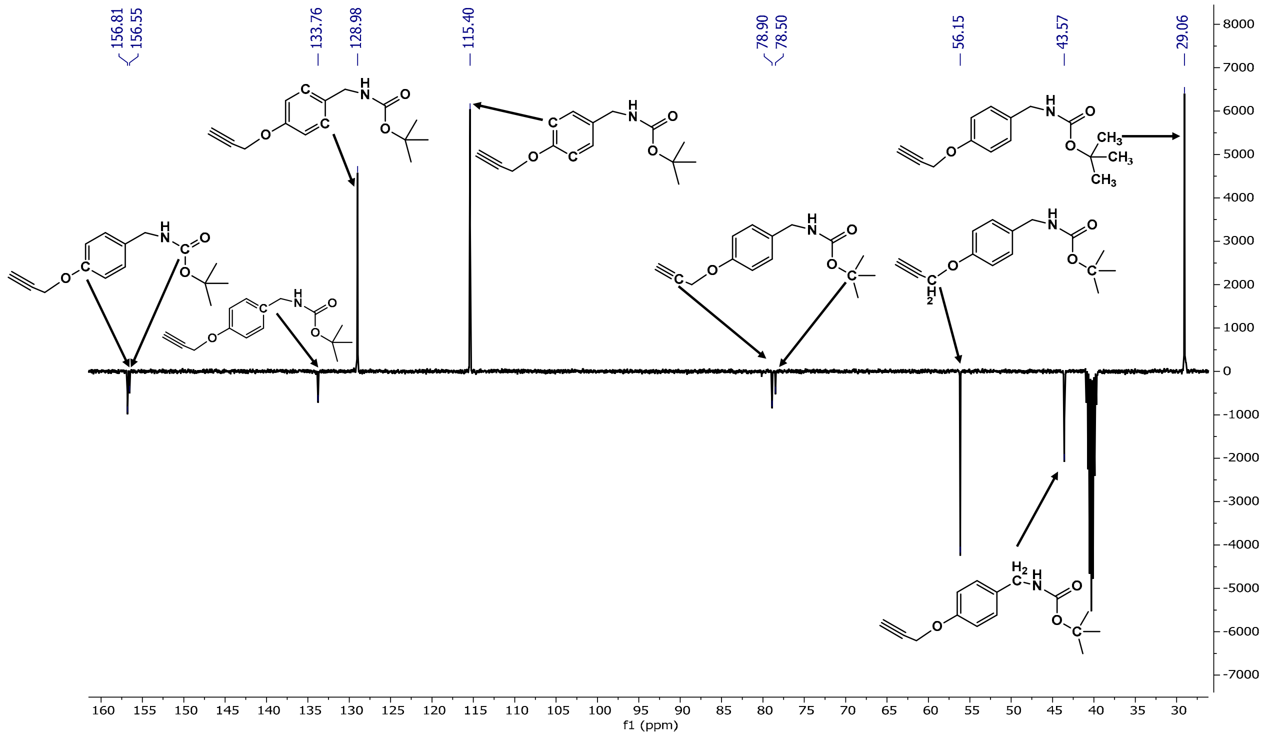

Figure S18. ^{13}C-NMR spectrum of *tert*-butyl(4-(prop-2-yn-1-yloxy)benzyl)carbamate in DMSO-d_6.

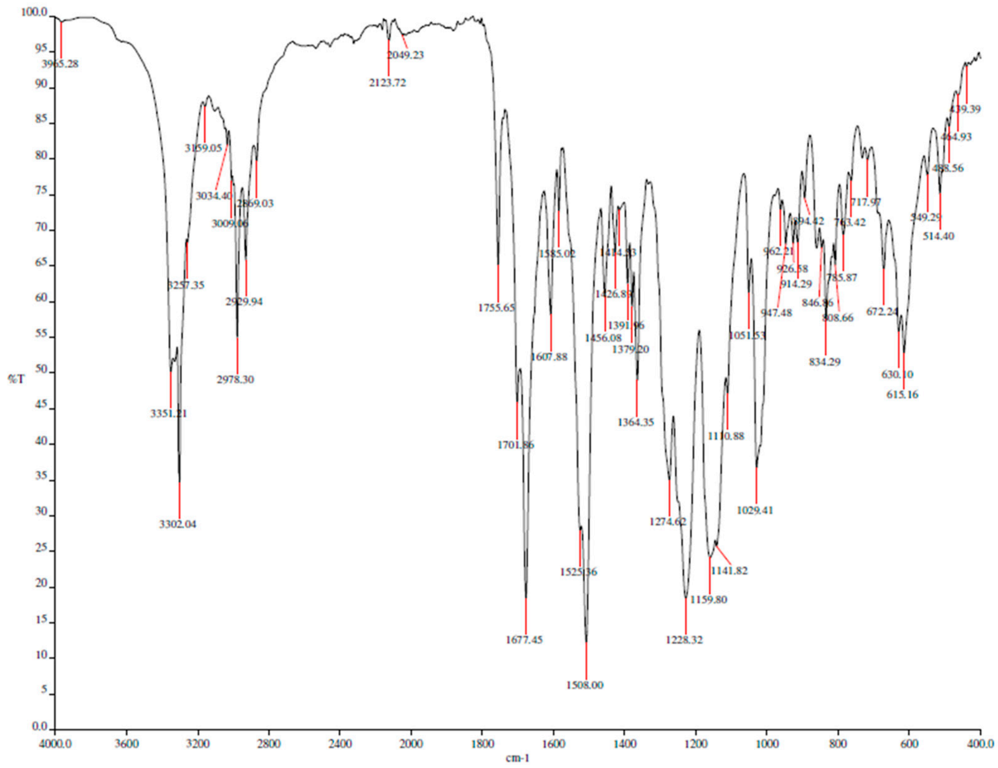

Figure S19. IR spectrum of *tert*-butyl(4-(prop-2-yn-1-yloxy)benzyl)carbamate.

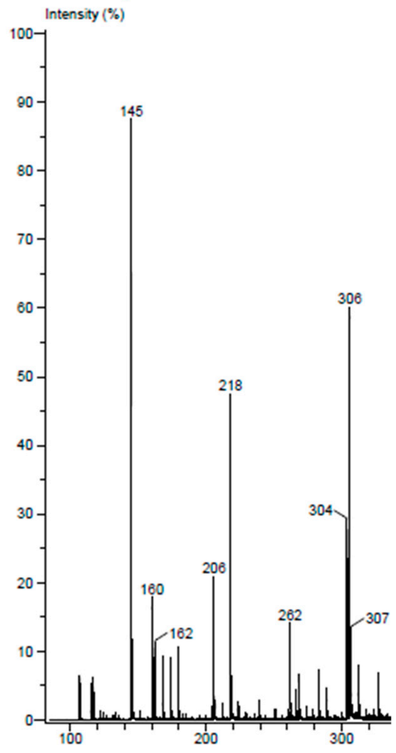

Figure S20. DART of *tert*-butyl(4-(prop-2-yn-1-yloxy)benzyl)carbamate.

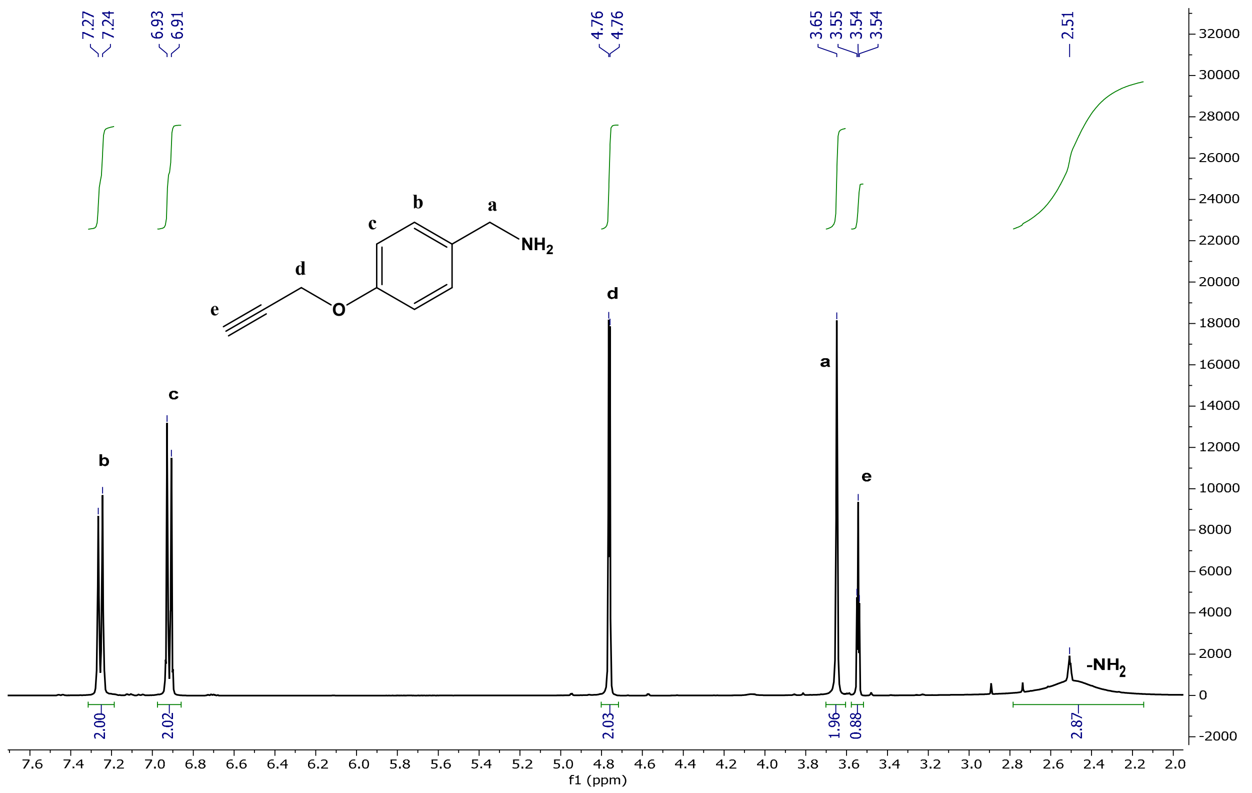

Figure S21. ^{1}H-NMR spectrum of [4-(prop-2-yn-1-yloxy)phenyl]methanamine in DMSO-d_6.

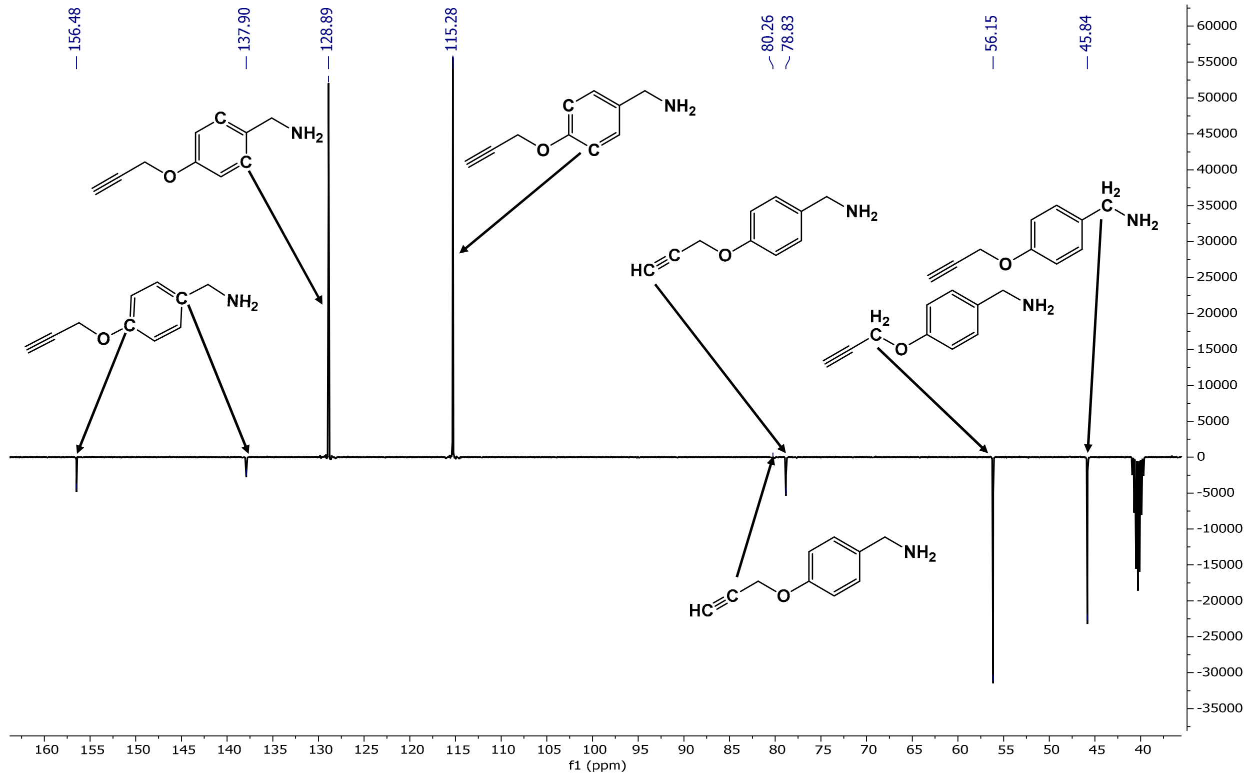

Figure S22. ^{13}C-NMR spectrum of [4-(prop-2-yn-1-yloxy)phenyl]methanamine in DMSO-d_6.

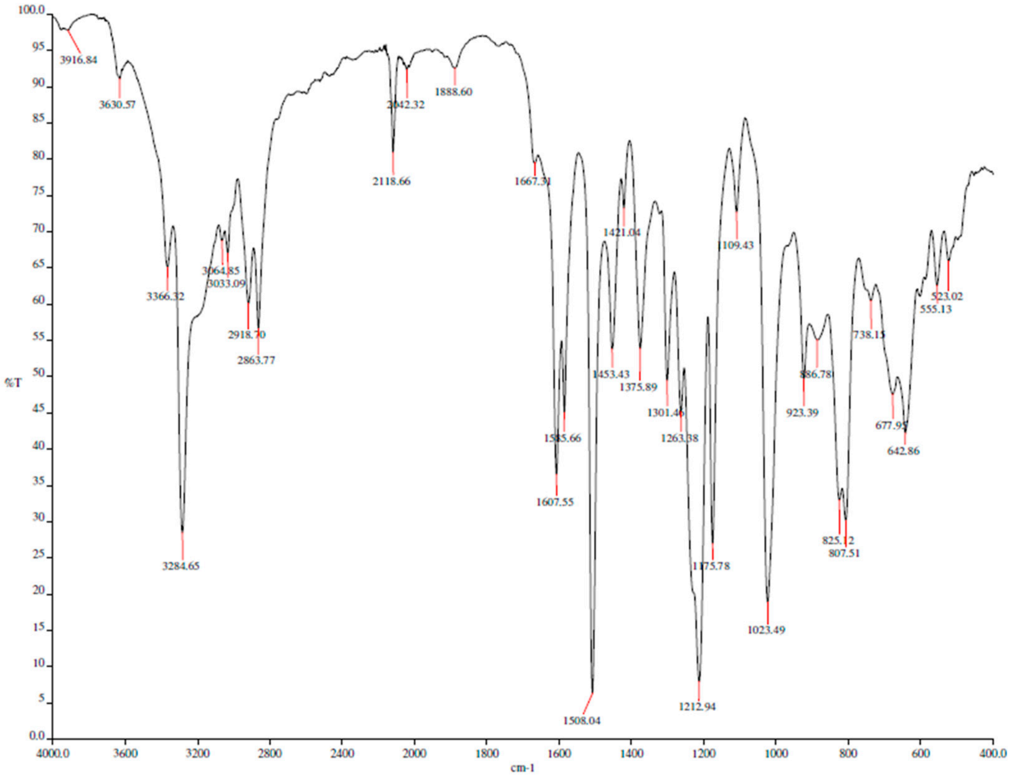

Figure S23. IR spectrum of [4-(prop-2-yn-1-yloxy)phenyl]methanamine.

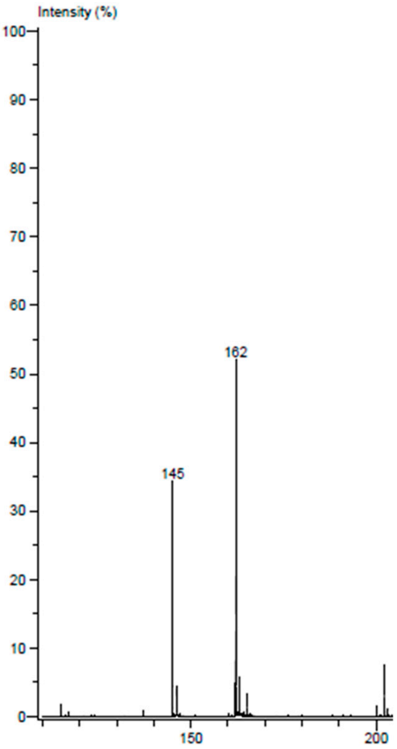

Figure S24. DART of [4-(prop-2-yn-1-yloxy)phenyl]methanamine.

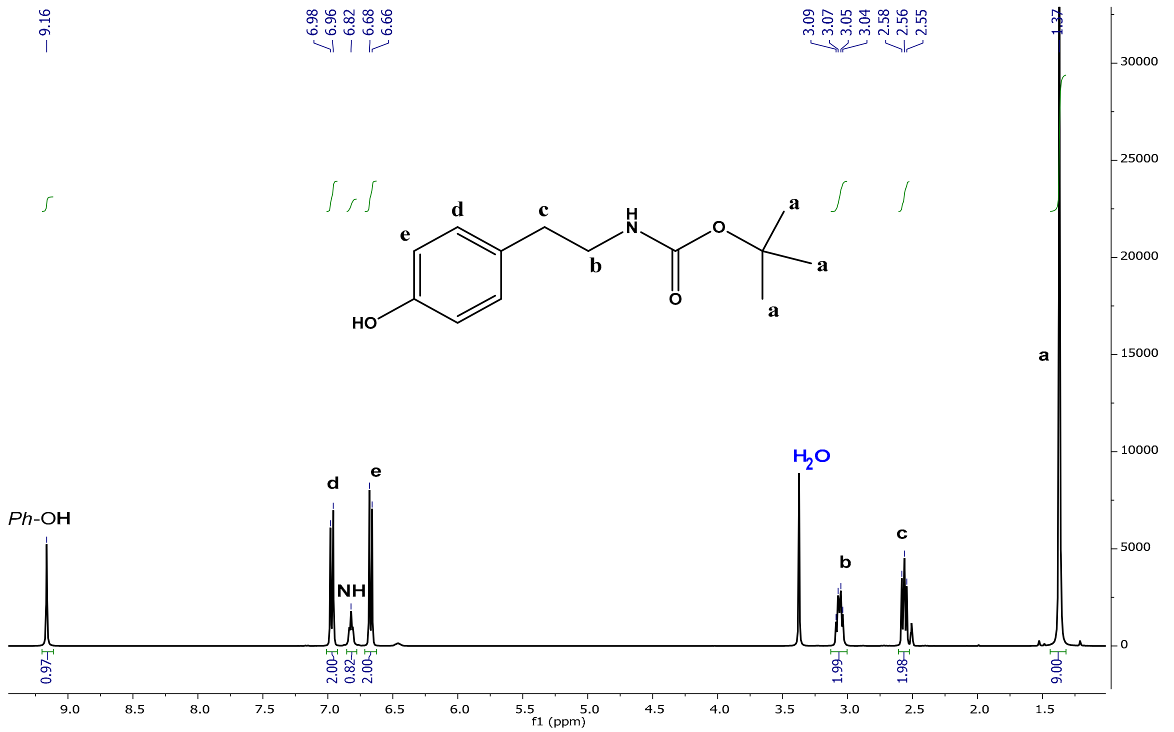

Figure S25. ^{1}H-NMR spectrum of *tert*-butyl (4-hydroxyphenethyl)carbamate in DMSO-d_6.

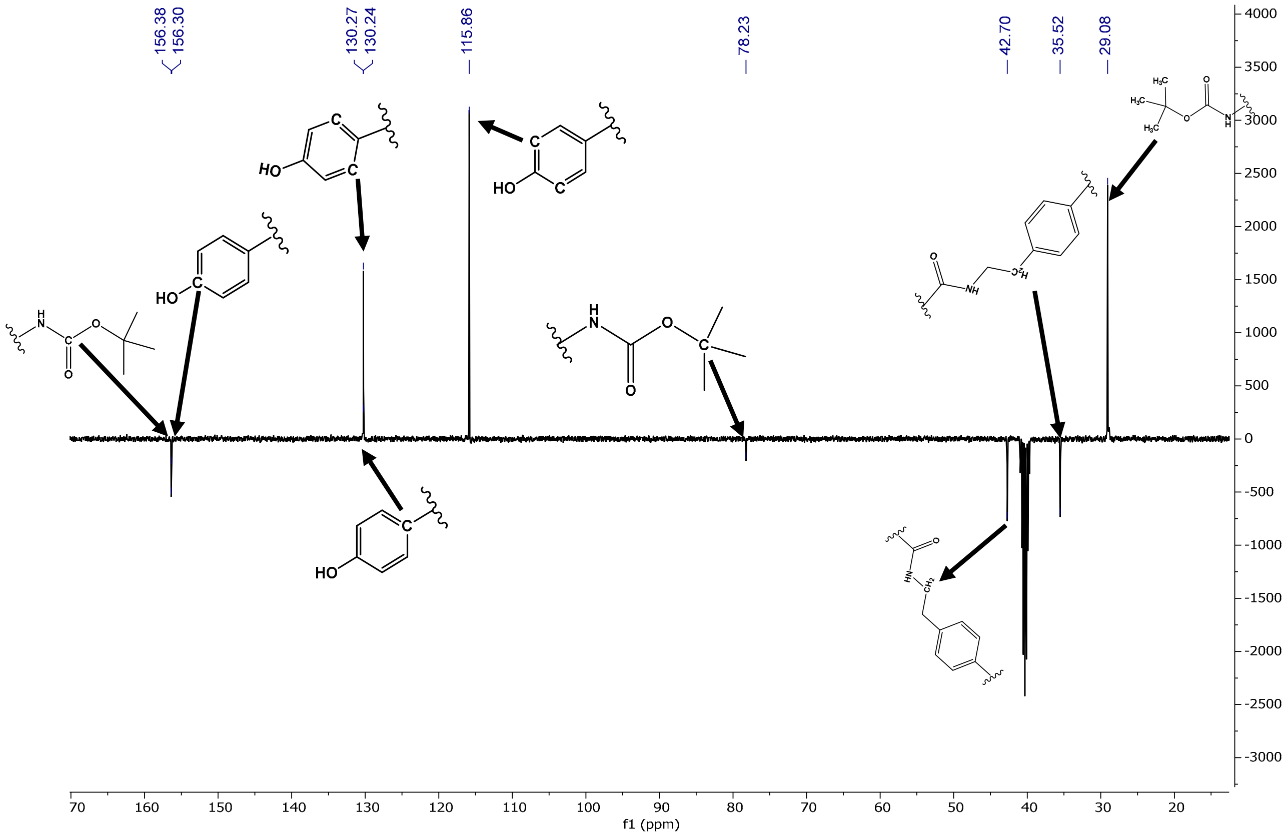

Figure S26. ^{13}C-NMR spectrum of *tert*-butyl (4-hydroxyphenethyl)carbamate in DMSO-d_6.

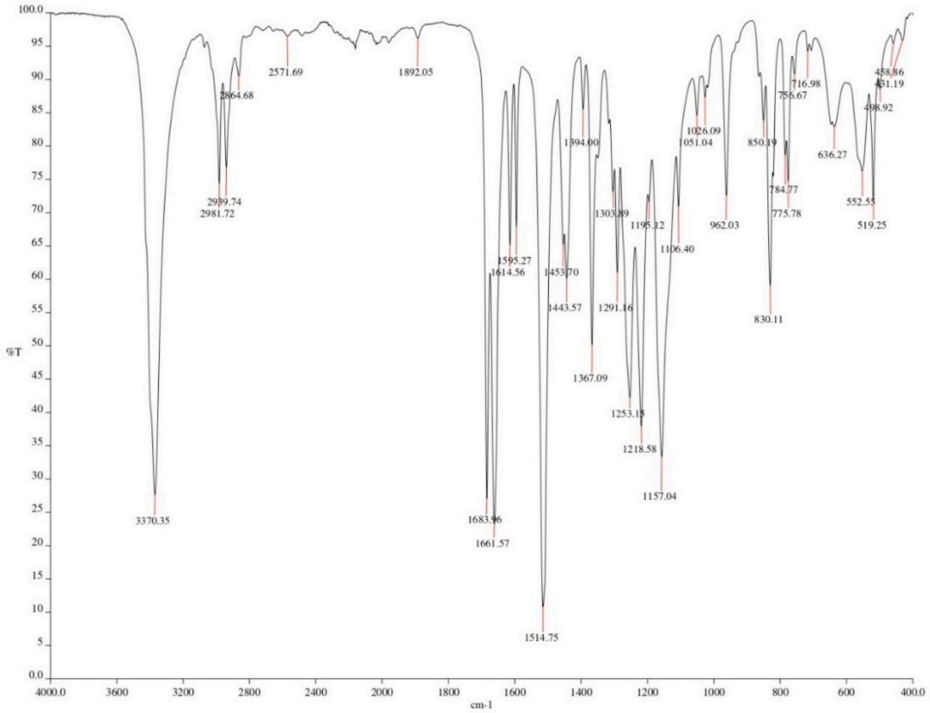

Figure S27. IR spectrum of *tert*-butyl (4-hydroxyphenethyl)carbamate.

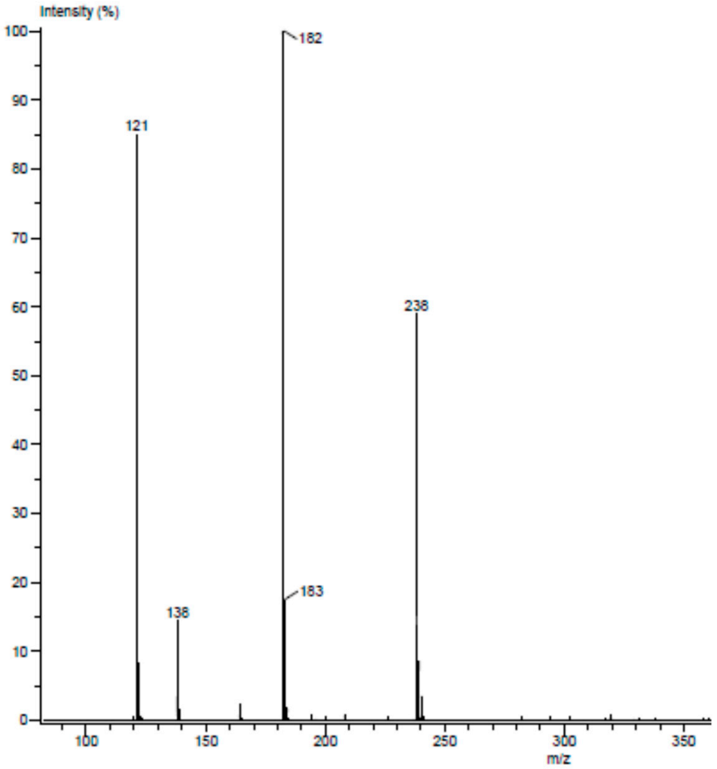

Figure S28. DART *tert*-butyl (4-hydroxyphenethyl)carbamate.

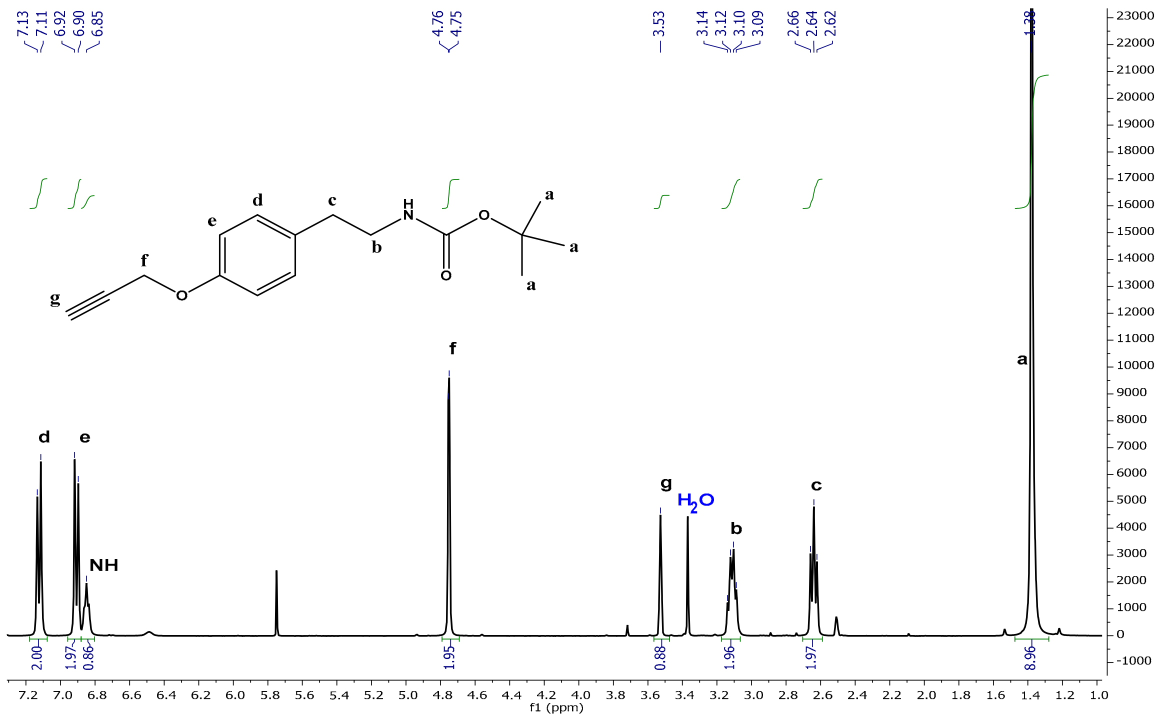

Figure S29. ^{1}H-NMR spectrum of *tert*-butyl (4-(prop-2-yn-1-yloxy)phenetyl)carbamate in DMSO-d_6.

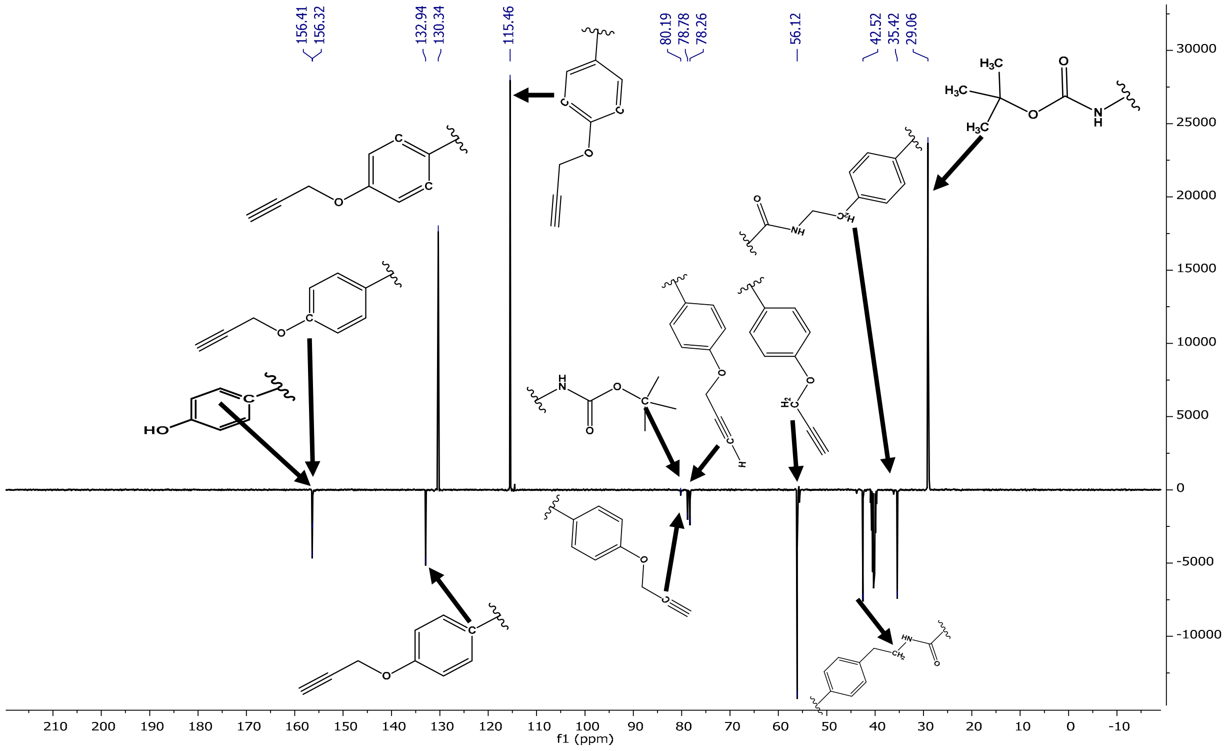

Figure S30. ^{13}C-NMR spectrum of *tert*-butyl (4-(prop-2-yn-1-yloxy)phenetyl)carbamate in DMSO-d_6.

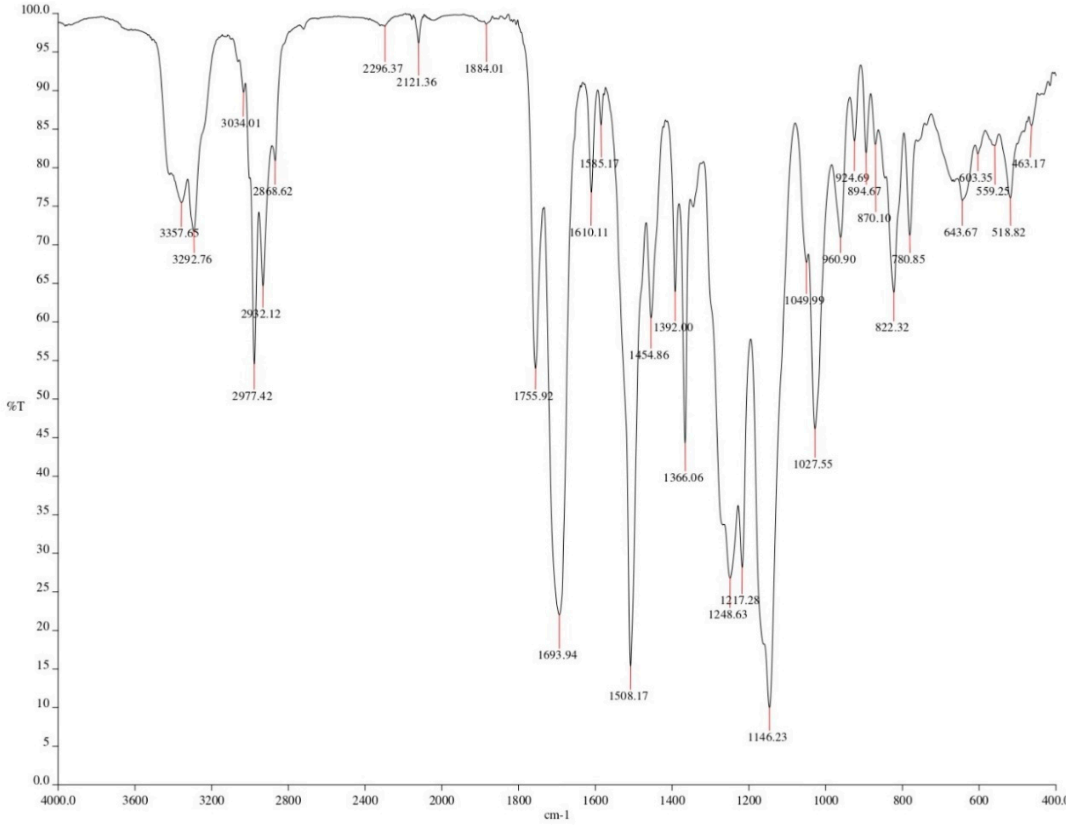

Figure S31. IR spectrum of *tert*-butyl (4-(prop-2-yn-1-yloxy)phenetyl)carbamate.

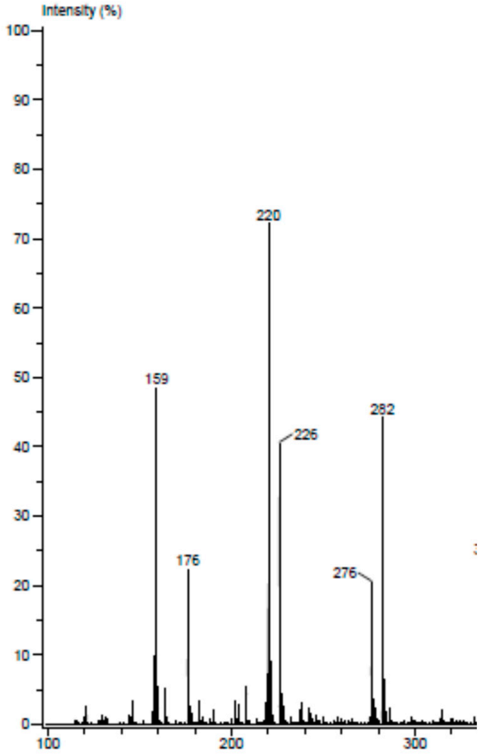

Figure S32. DART spectrum of *tert*-butyl (4-(prop-2-yn-1-yloxy)phenetyl)carbamate.

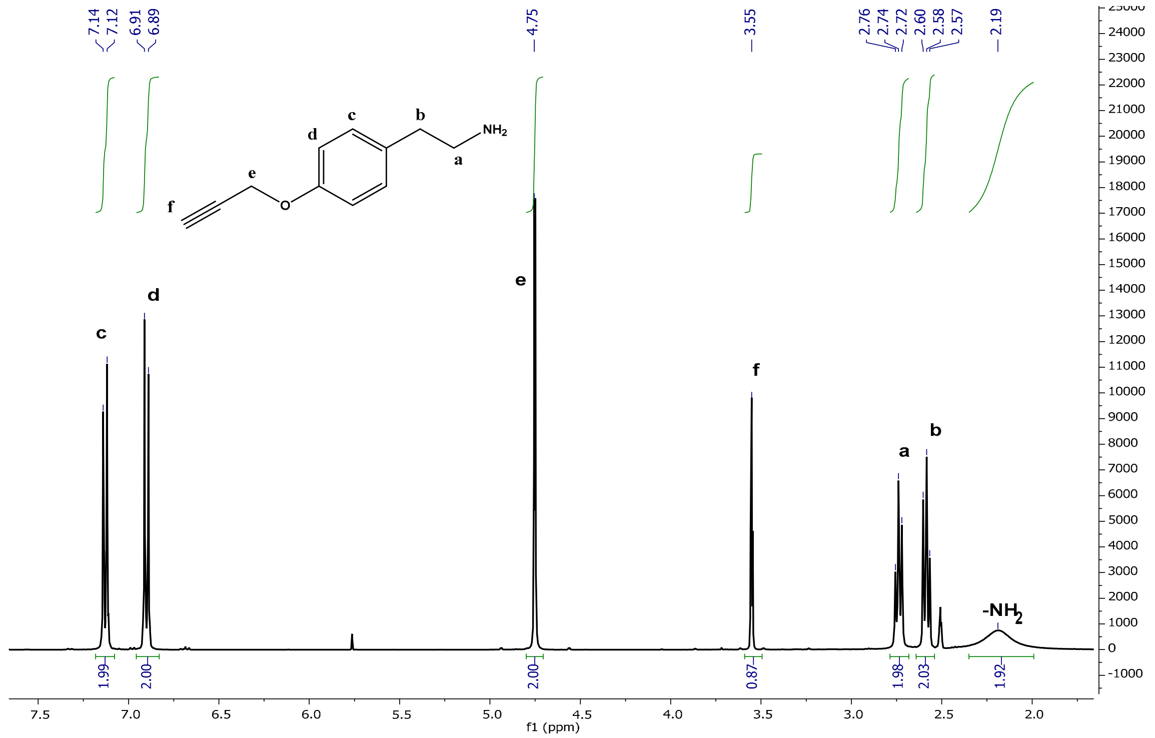

Figure S33. ^{1}H-NMR spectrum of 2-(4-(prop-2-yn-1-yloxy)phenyl)ethan-1-amine in DMSO-d_6.

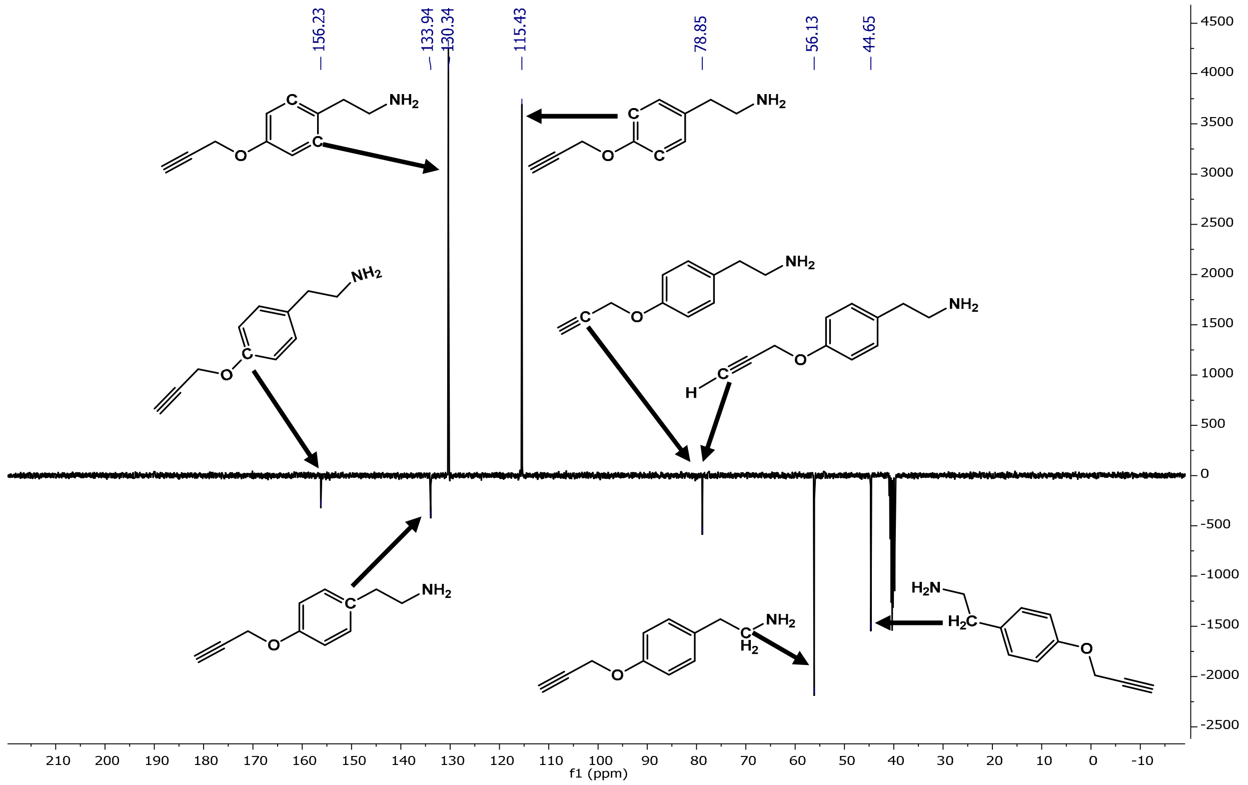

Figure S34. ^{13}C-NMR spectrum of 2-(4-(prop-2-yn-1-yloxy)phenyl)ethan-1-amine in DMSO-d_6.

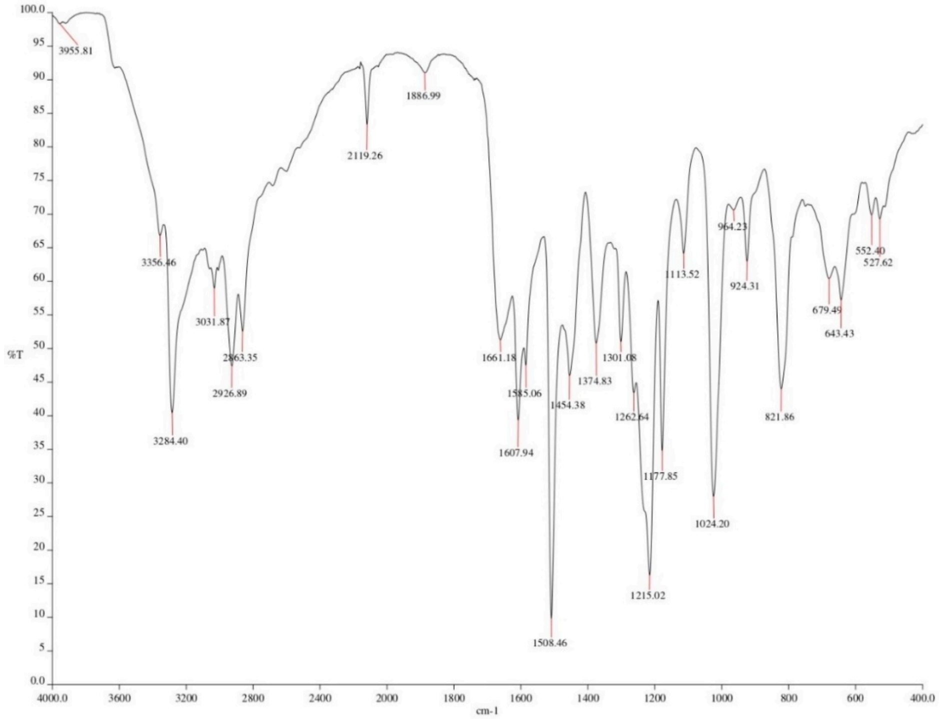

Figure S35. IR spectrum of 2-(4-(prop-2-yn-1-yloxy)phenyl)ethan-1-amine.

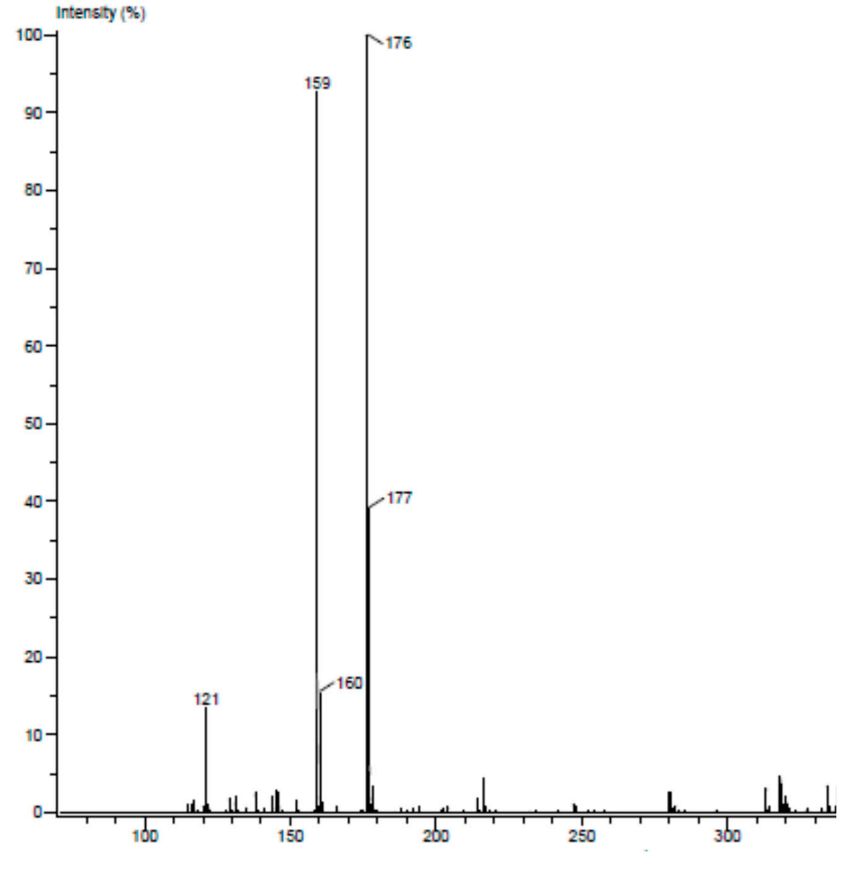

Figure S36. DART spectrum of 2-(4-(prop-2-yn-1-yloxy)phenyl)ethan-1-amine.

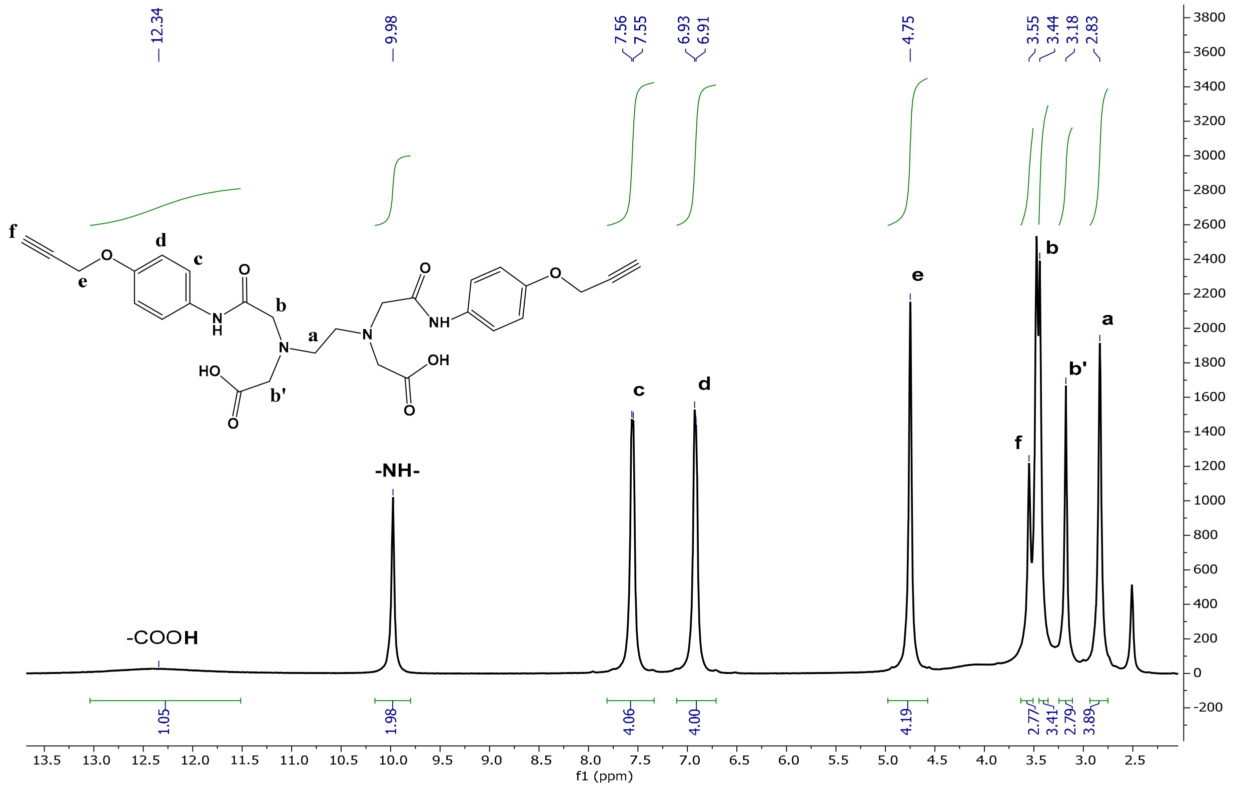

Figure S37. ¹H-NMR spectrum of disubstituted EDTA alkyne **13** in DMSO-d_6.

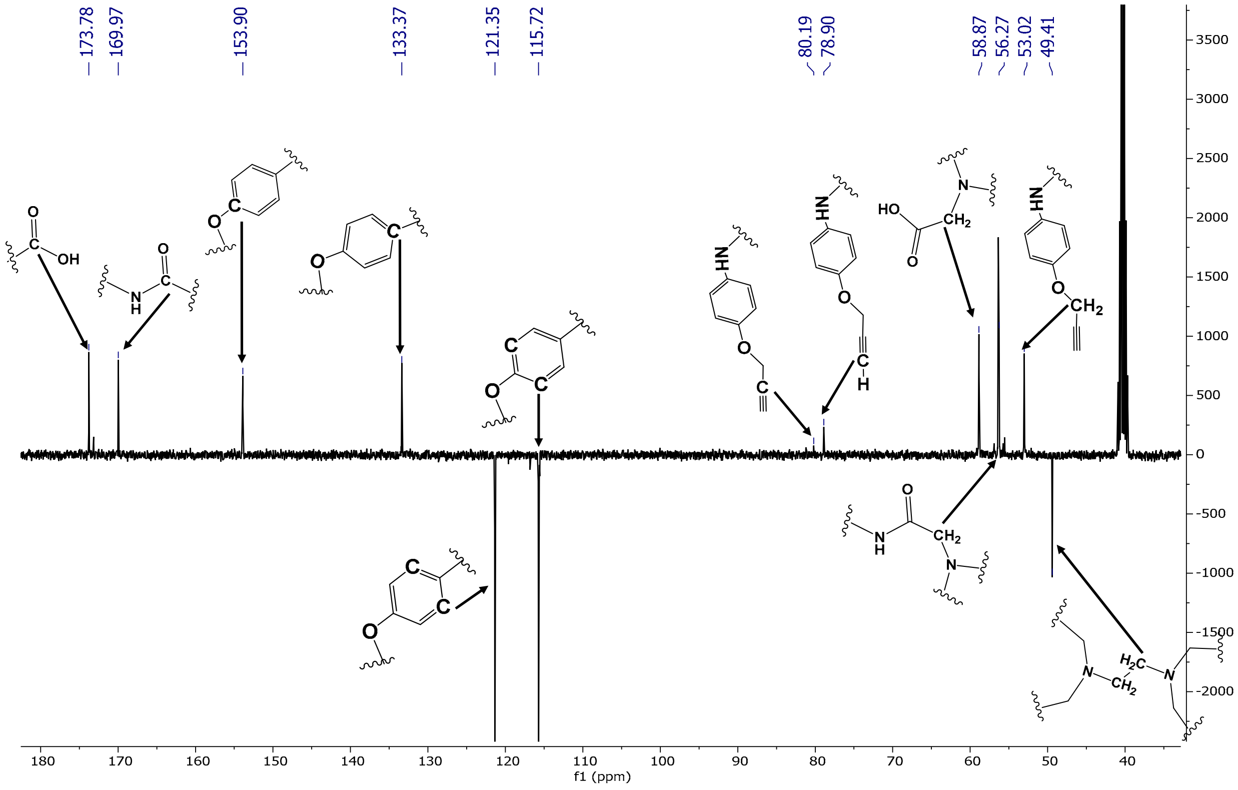

Figure S38. ¹³C-NMR spectrum of disubstituted EDTA alkyne **13** in DMSO-d_6.

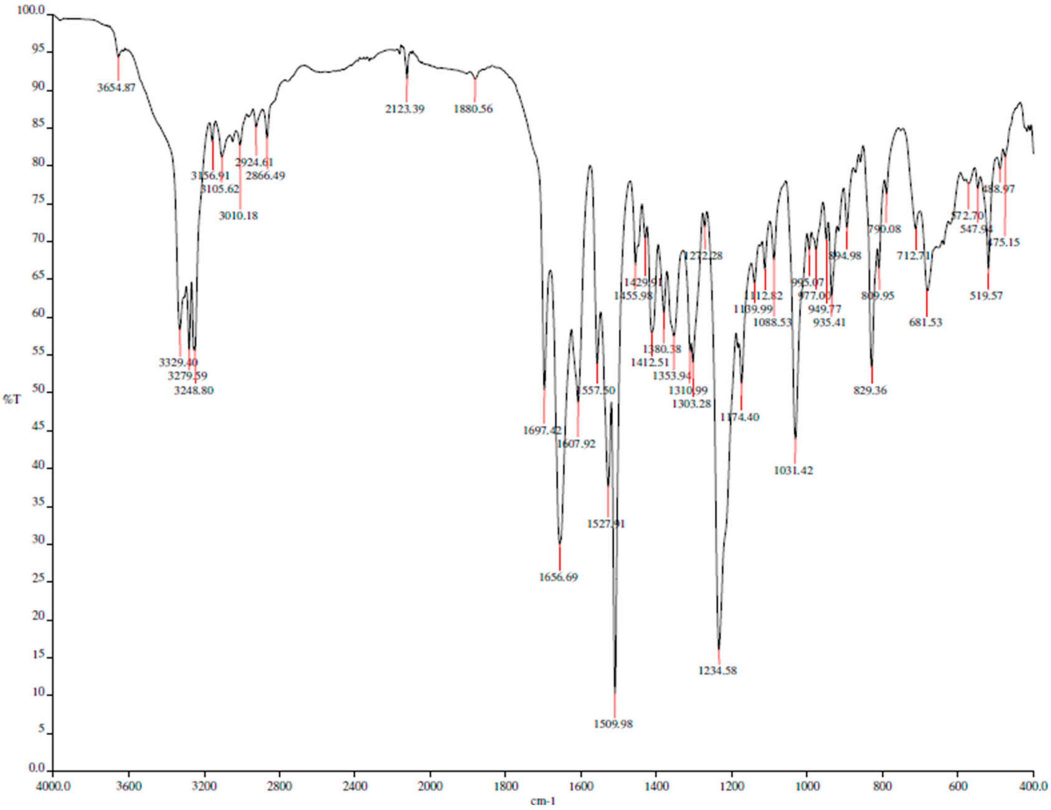

Figure S39. IR spectrum of disubstituted EDTA alkyne **13**.

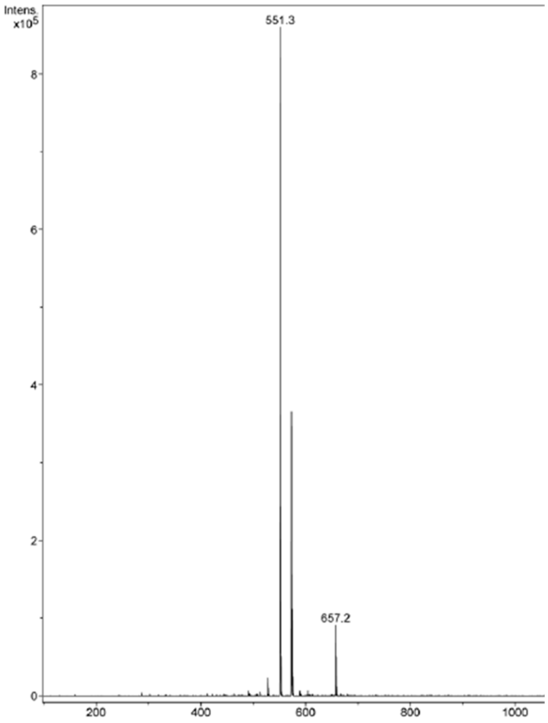

Figure S40. DART spectrum of disubstituted EDTA alkyne **13**.

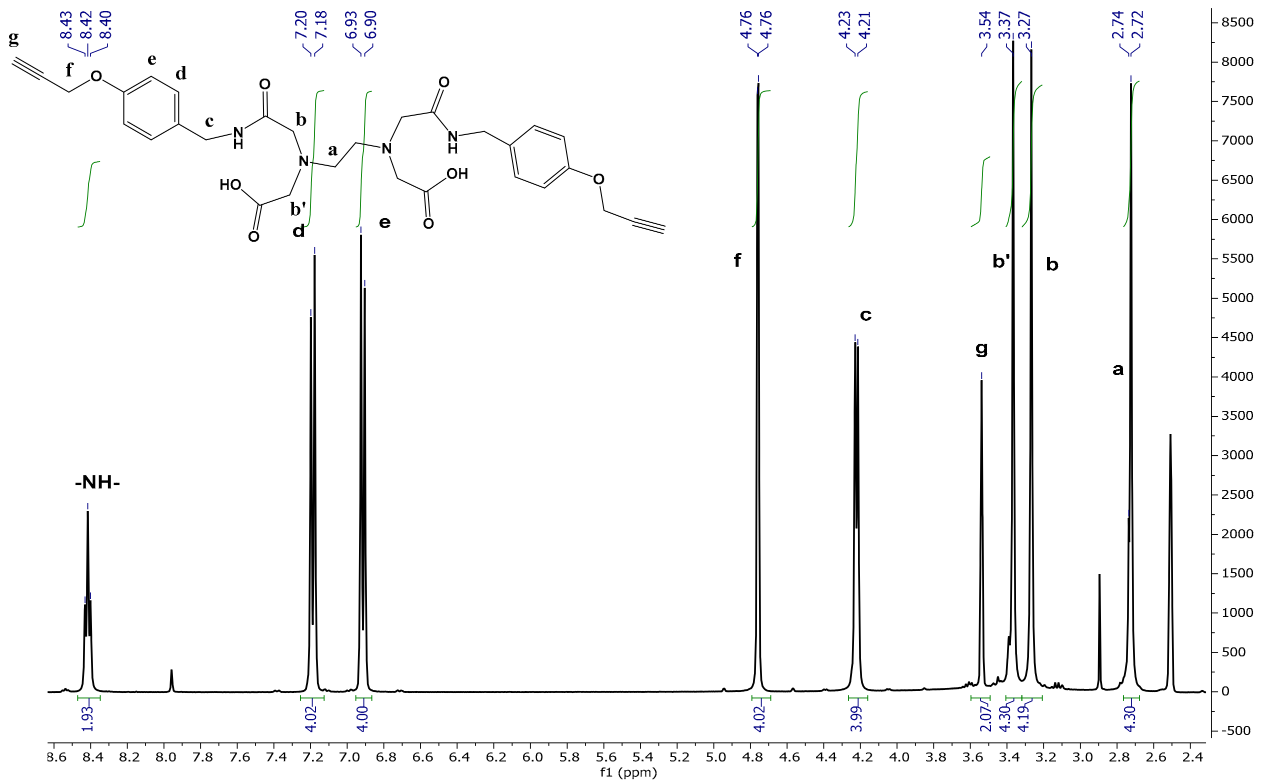

Figure S41. ^{1}H-NMR spectrum of disubstituted EDTA alkyne **14** in DMSO-d_6.

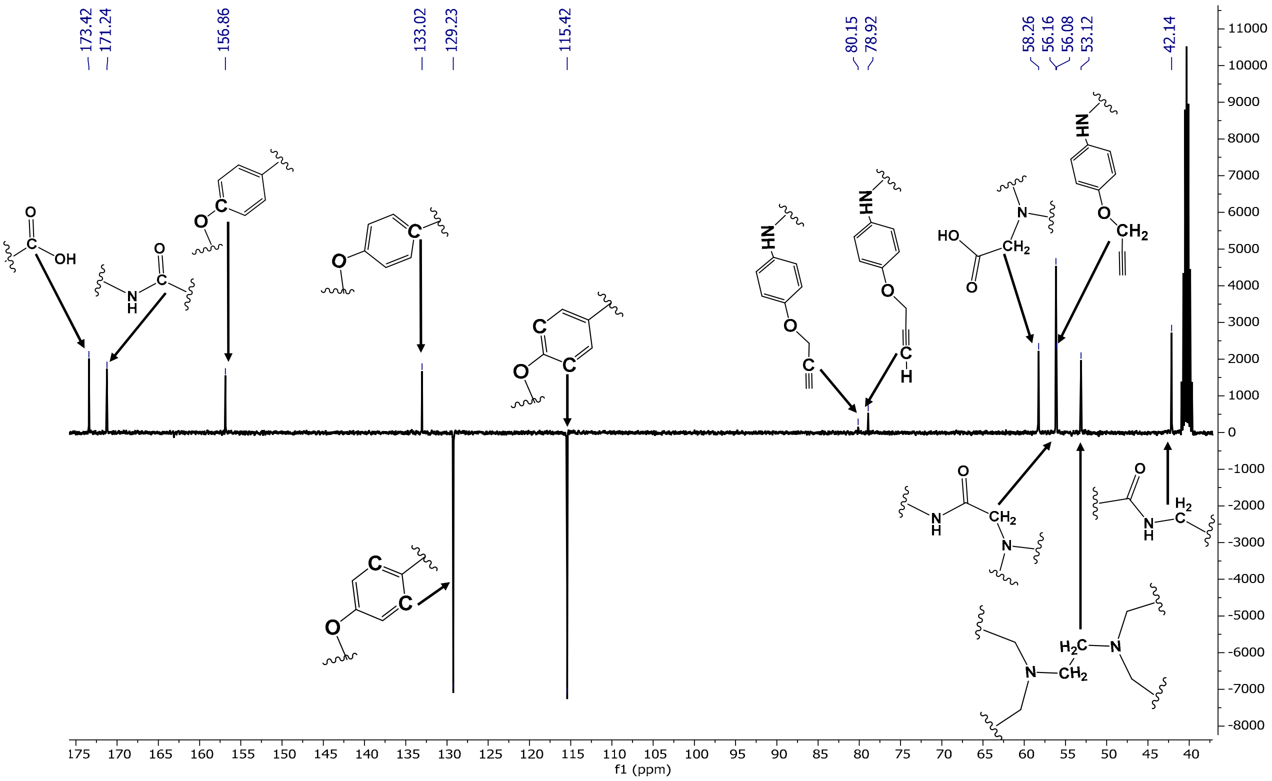

Figure S42. ^{13}C-NMR spectrum of disubstituted EDTA alkyne **14** in DMSO-d_6.

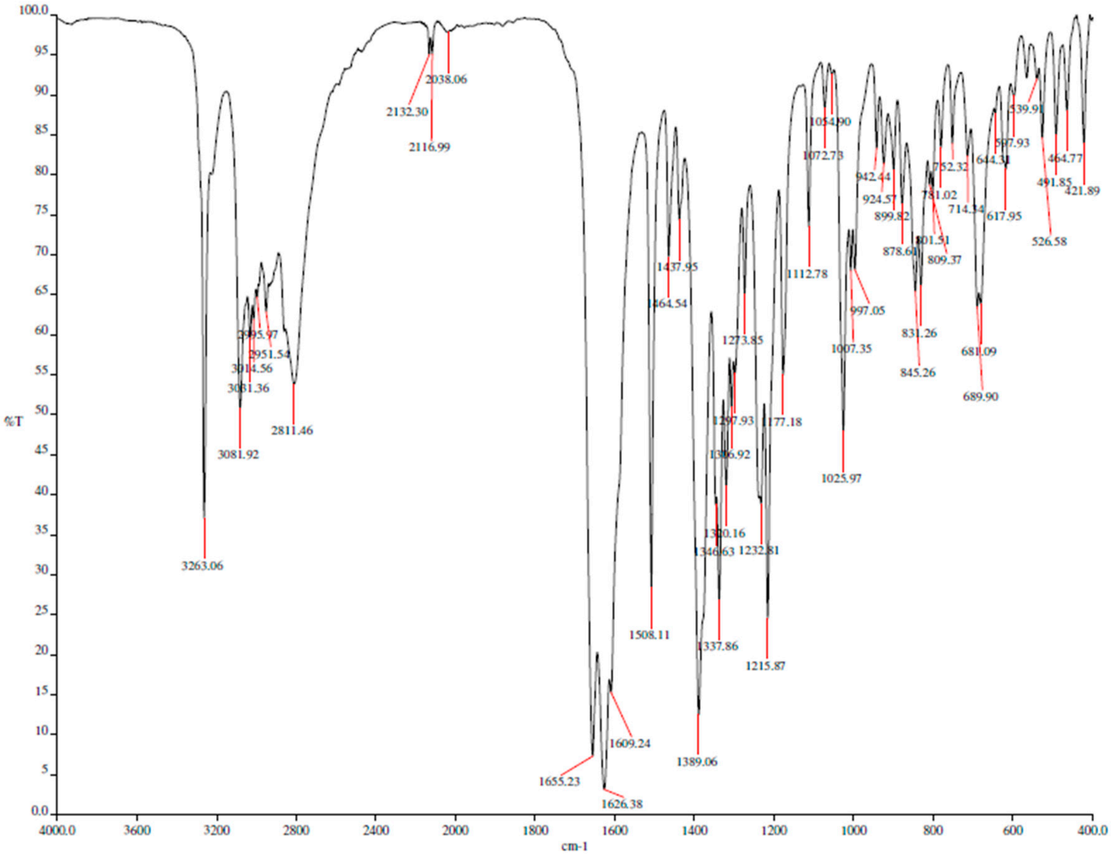

Figure S43. IR spectrum of disubstituted EDTA alkyne **14**.

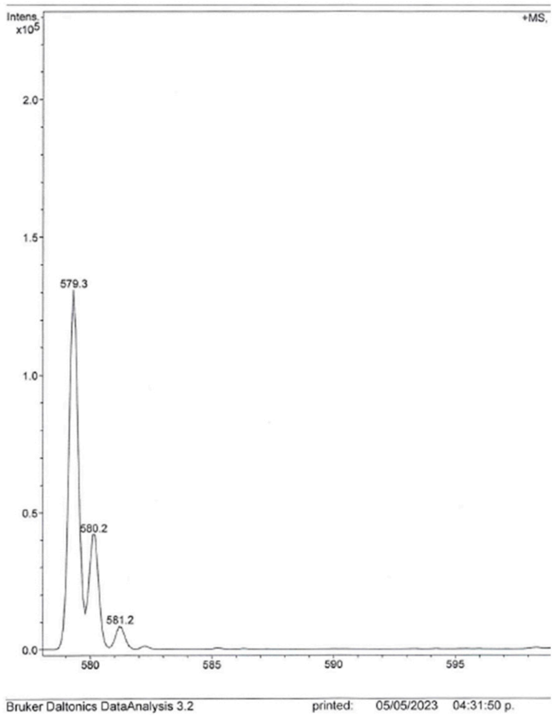

Figure S44. DART spectrum of disubstituted EDTA alkyne **14**.

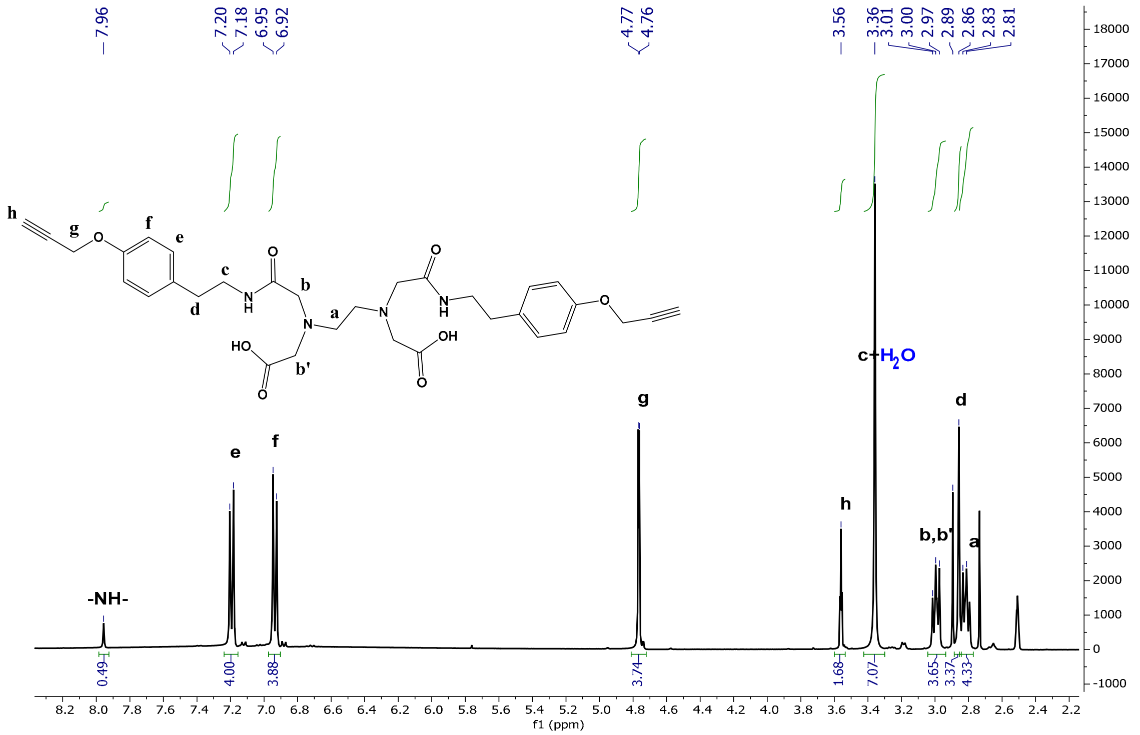

Figure S45. ^{1}H-NMR spectrum of disubstituted EDTA alkyne **15** in DMSO-d_6.

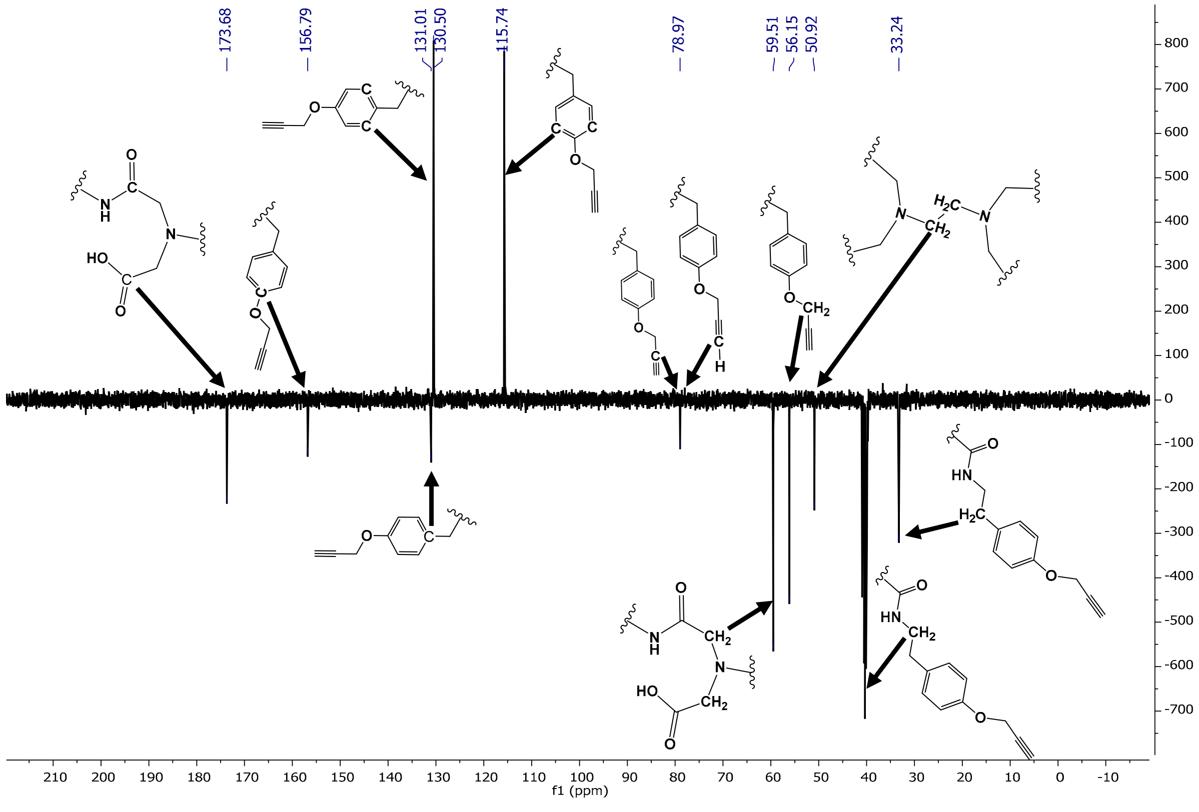

Figure S46. ^{13}C-NMR spectrum of disubstituted EDTA alkyne **15** in DMSO-d_6.

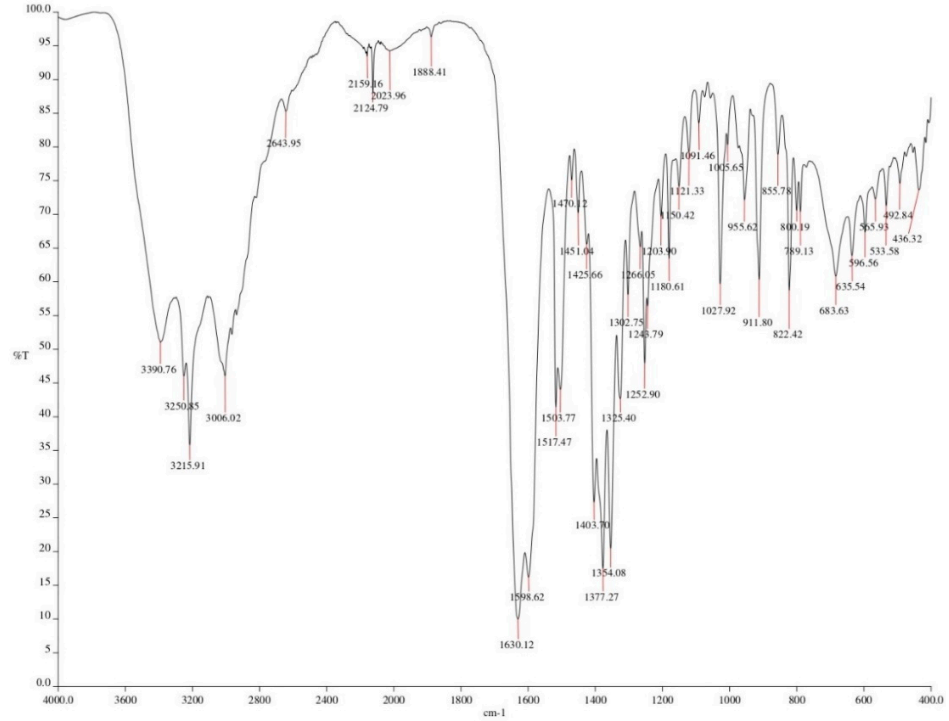

Figure S47. IR spectrum of disubstituted EDTA alkyne **15**.

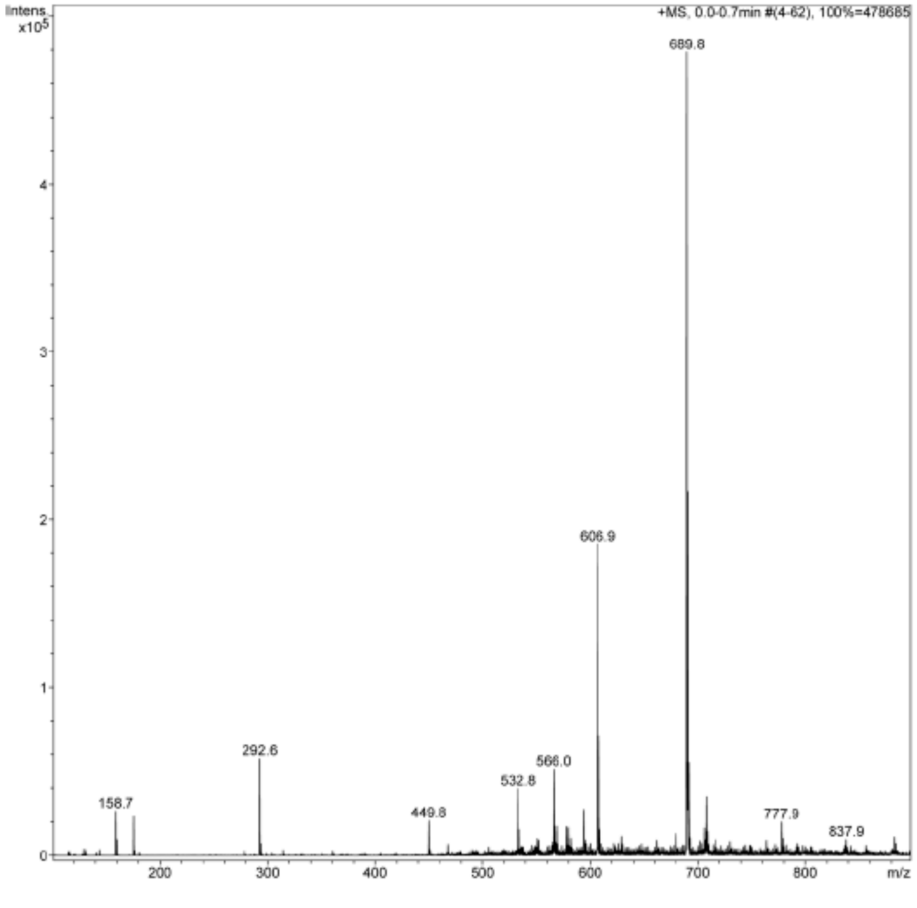

Figure S48. ESI spectrum of disubstituted EDTA alkyne **15**.

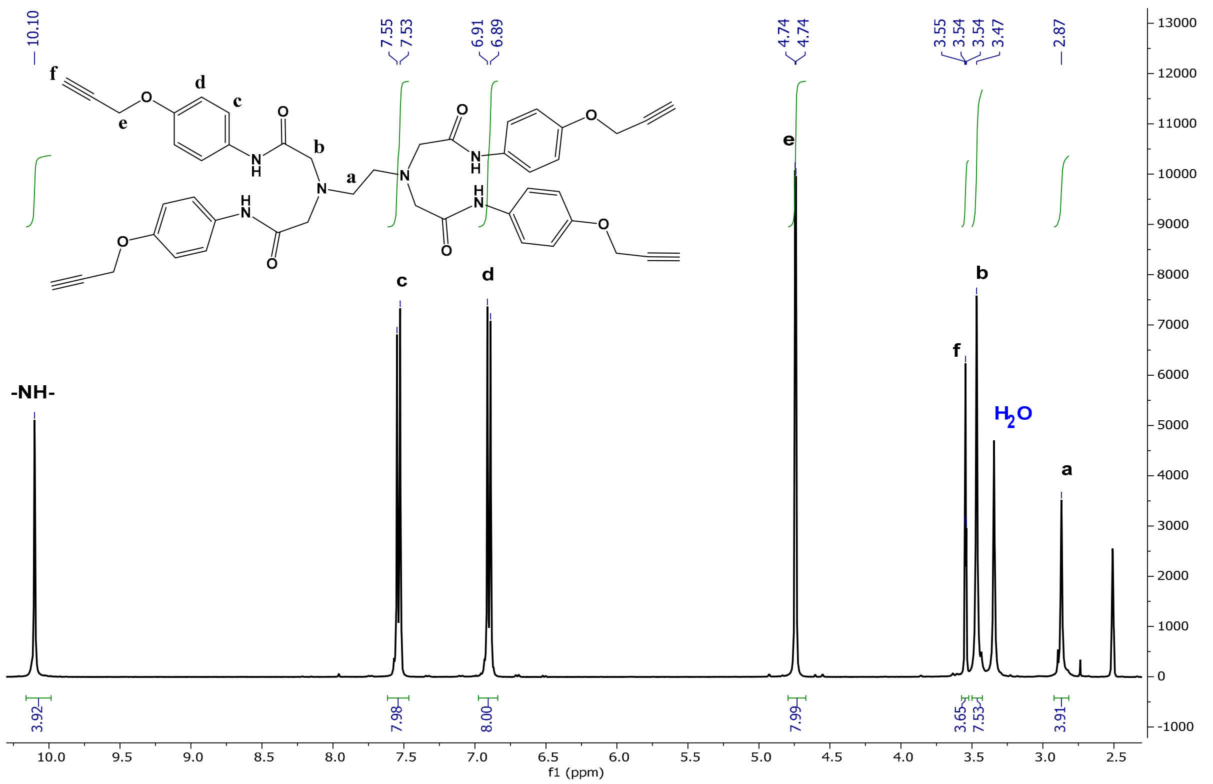

Figure S49. ^{1}H-NMR spectrum of EDTA G0-alkyne **16** in DMSO-d_6.

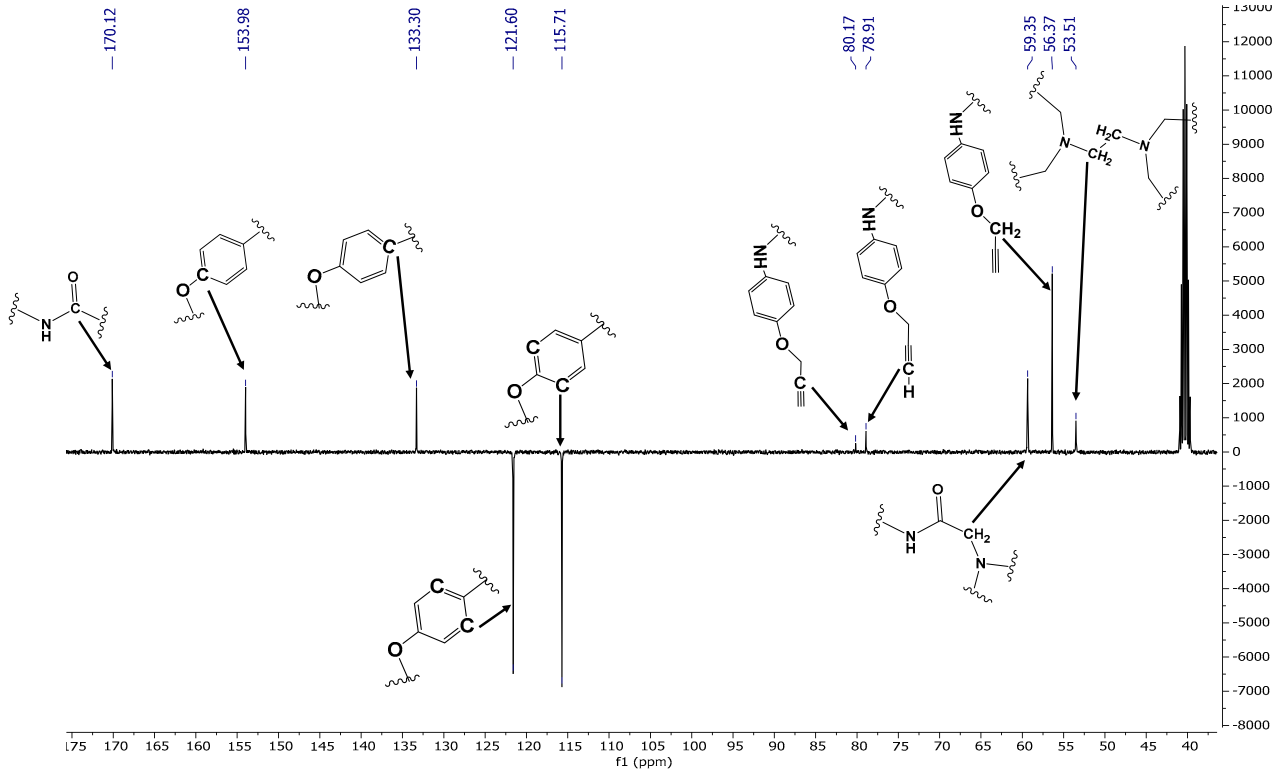

Figure S50. ^{13}C-NMR spectrum of EDTA G0-alkyne **16** in DMSO-d_6.

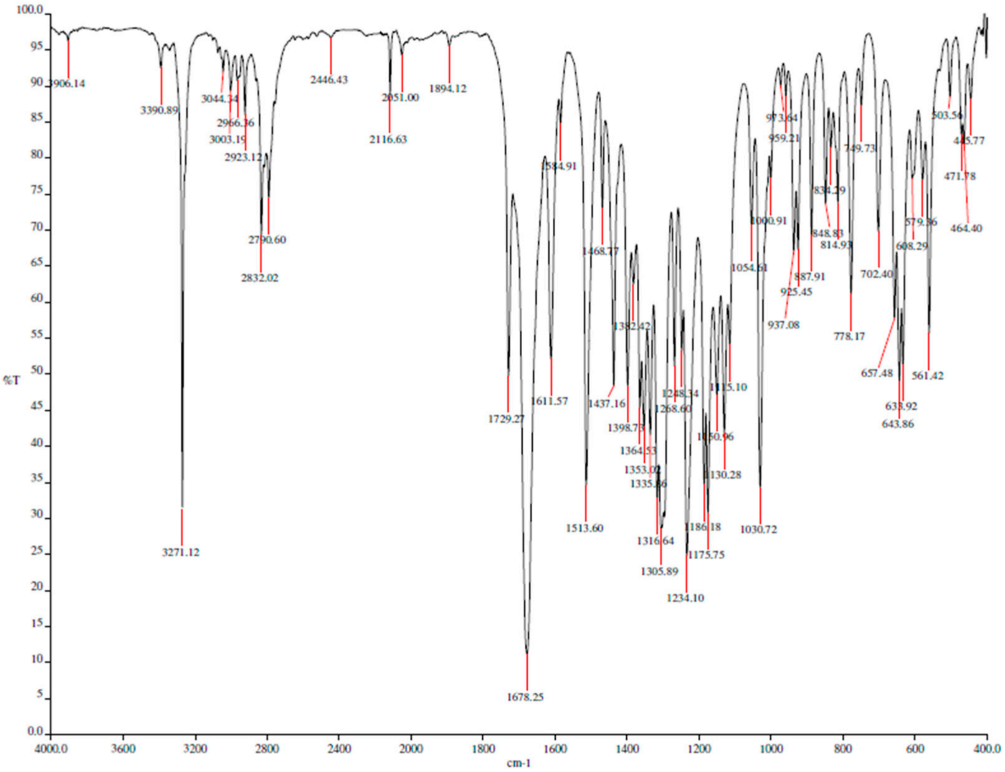

Figure S51. IR spectrum of EDTA G0-alkyne **16**.

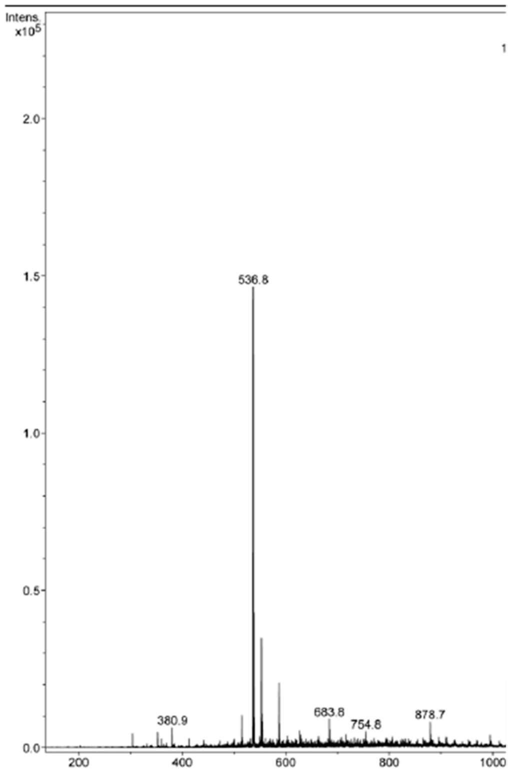

Figure S52. ESI spectrum of disubstituted EDTA alkyne **16**.

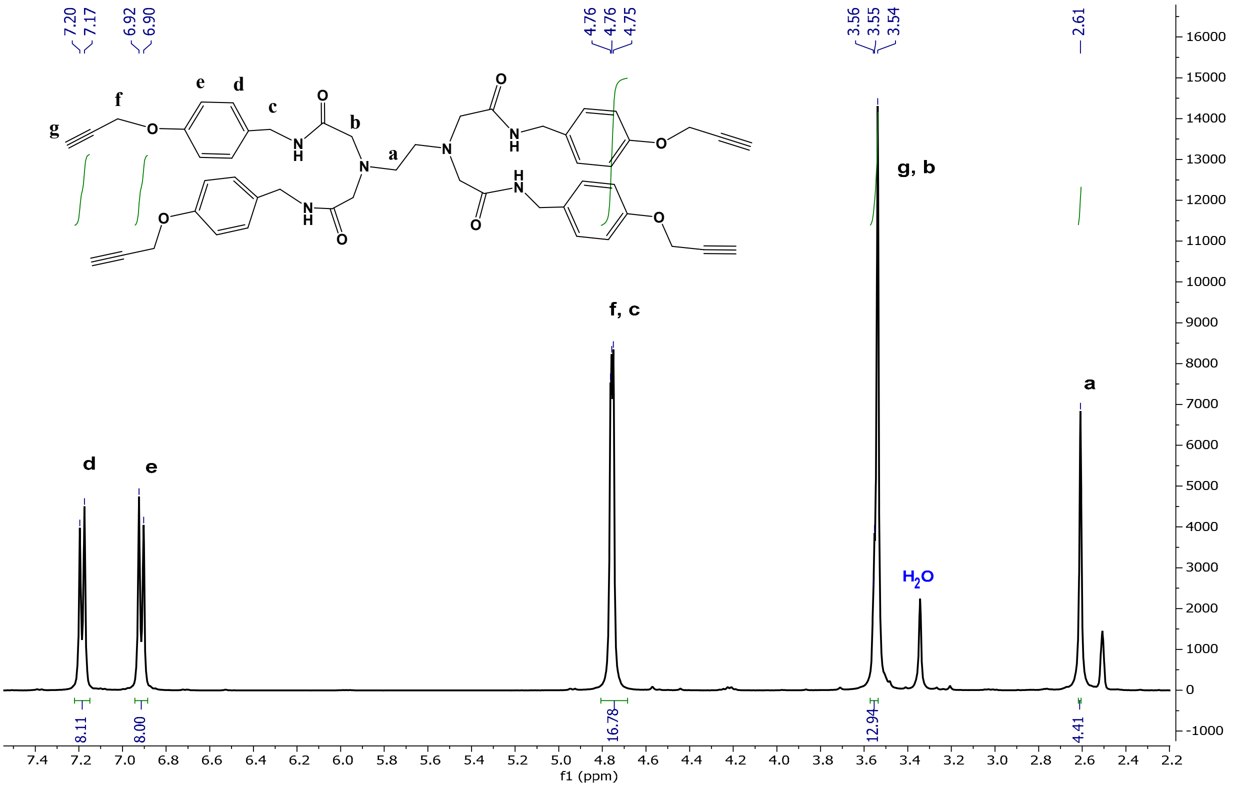

Figure S53. ^{1}H-NMR spectrum of EDTA G0-alkyne **17** in DMSO-d_6.

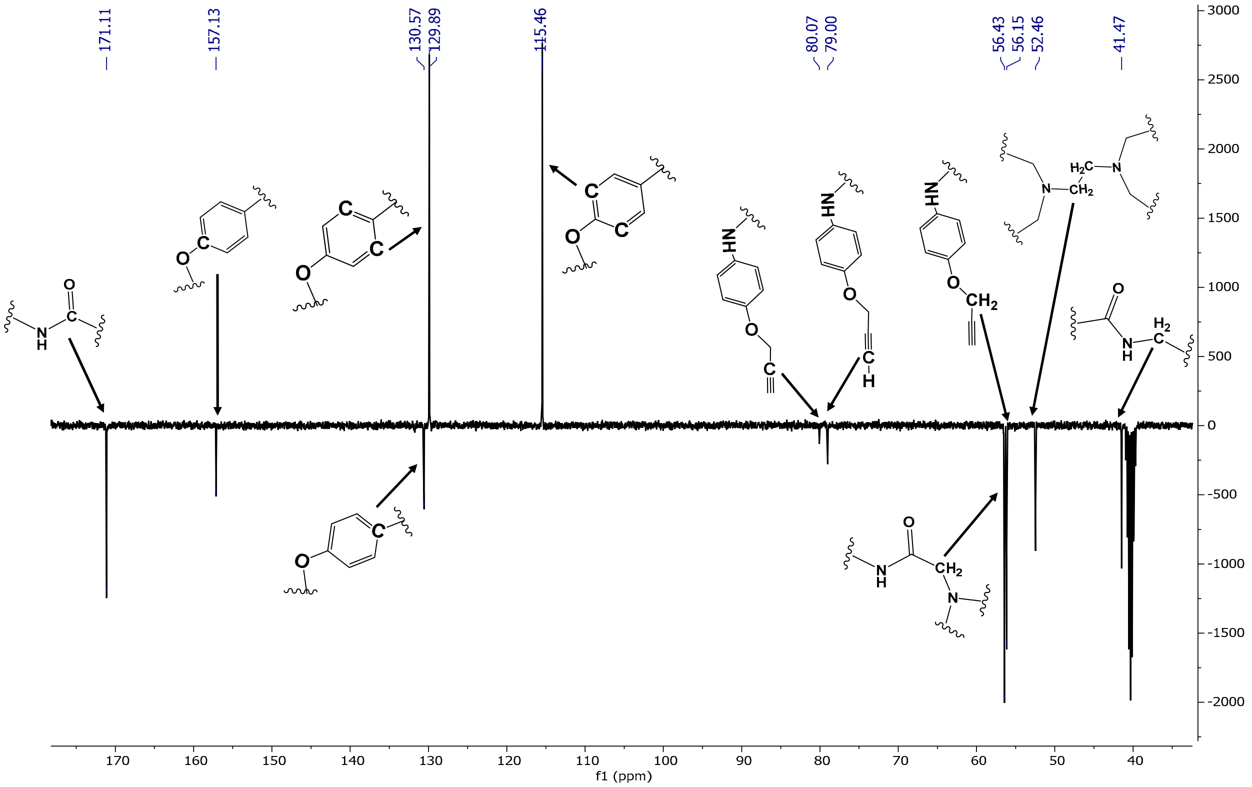

Figure S54. ^{13}C-NMR spectrum of EDTA G0-alkyne **17** in DMSO-d_6.

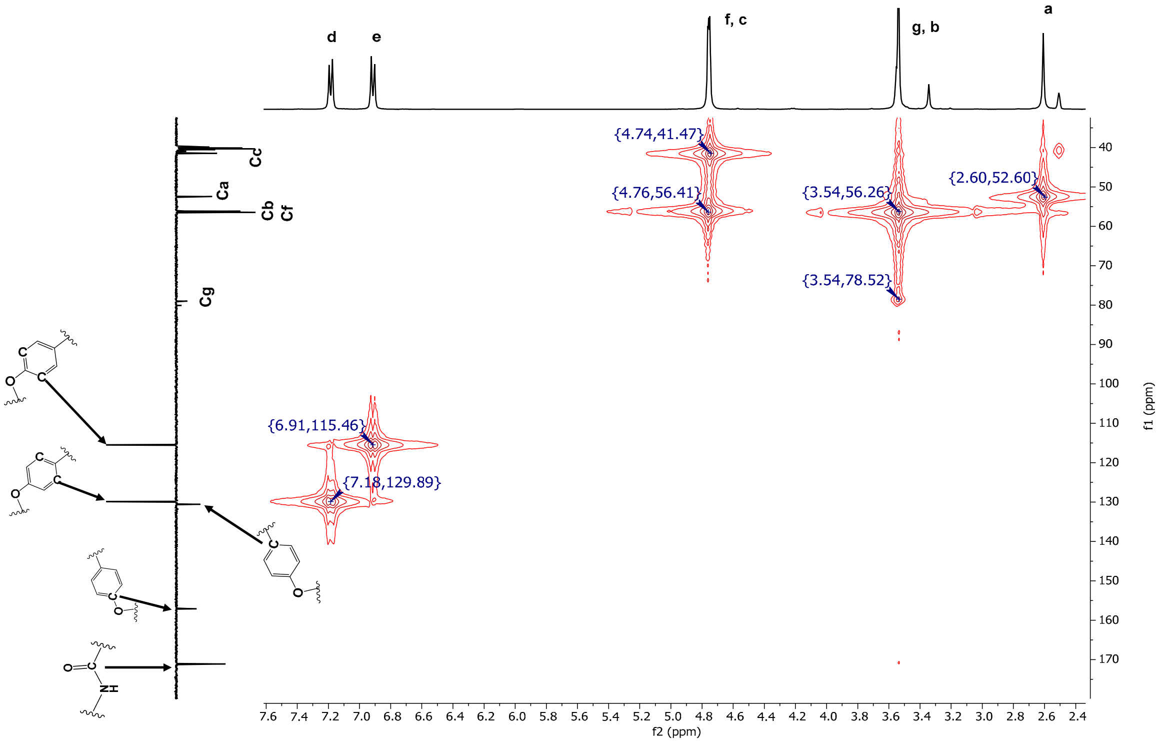

Figure S55. 2D NMR HMQC spectrum of EDTA G0-alkyne **17** in DMSO-d_6.

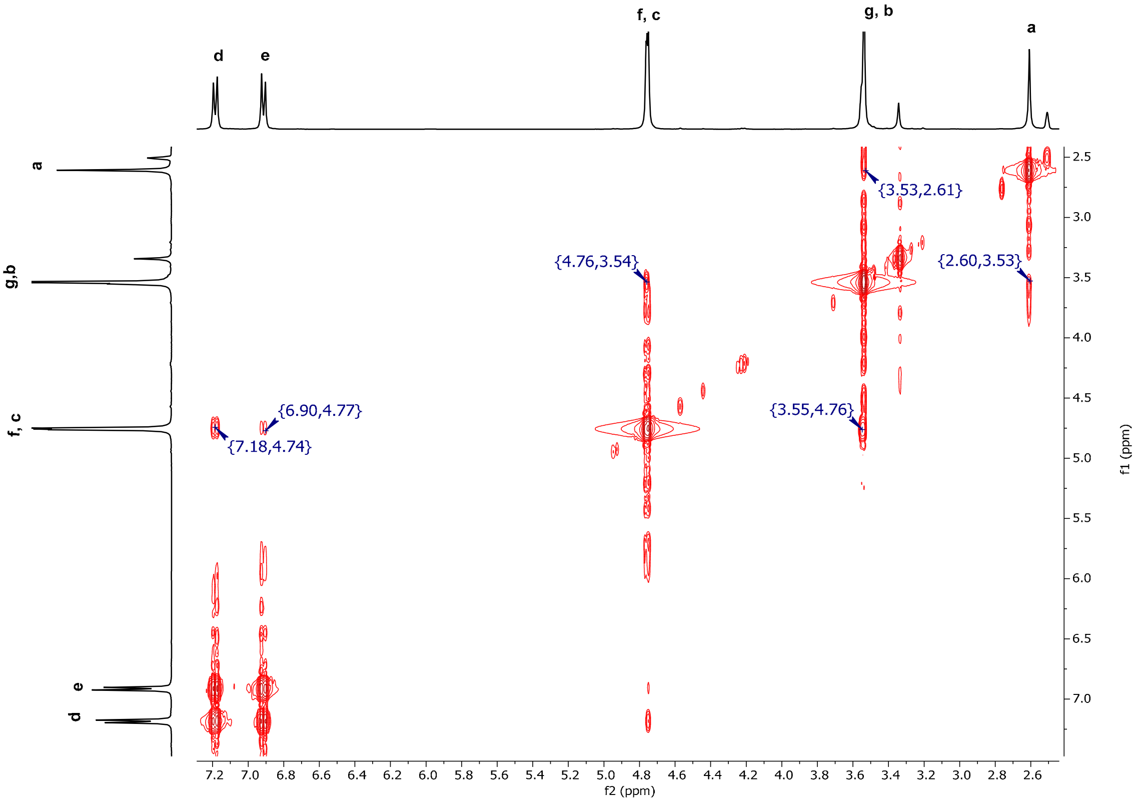

Figure S56. 2D NMR COSY spectrum of EDTA G0-alkyne **17** in DMSO-d_6.

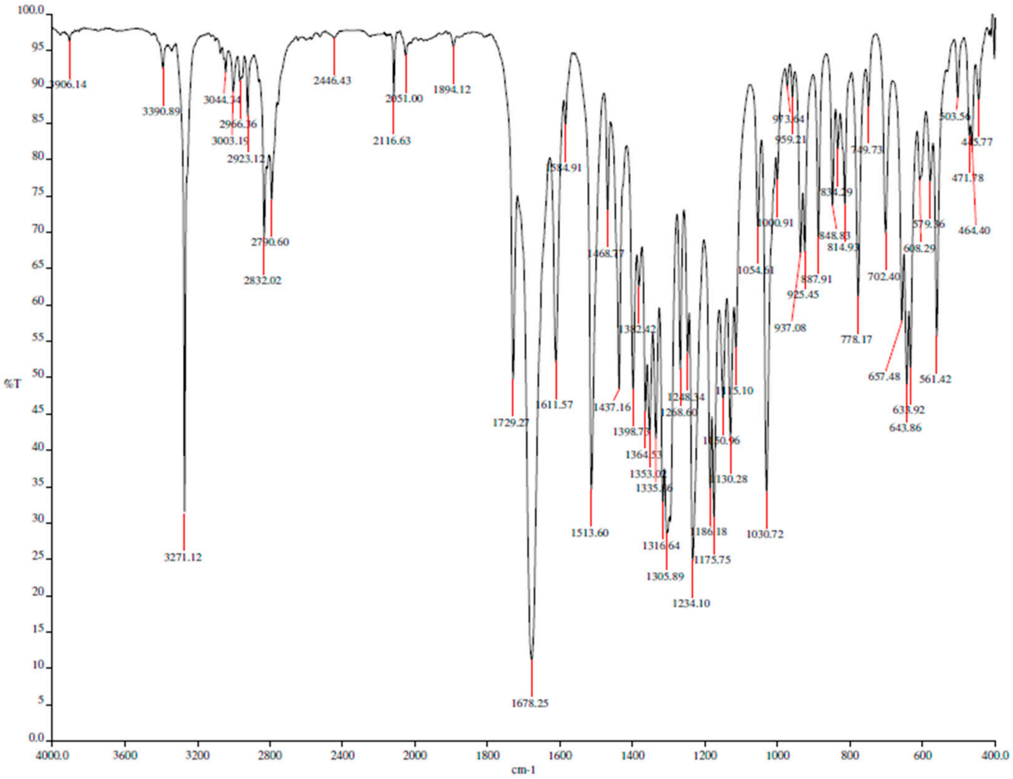

Figure S57. IR spectrum of EDTA G0-alkyne **17**.

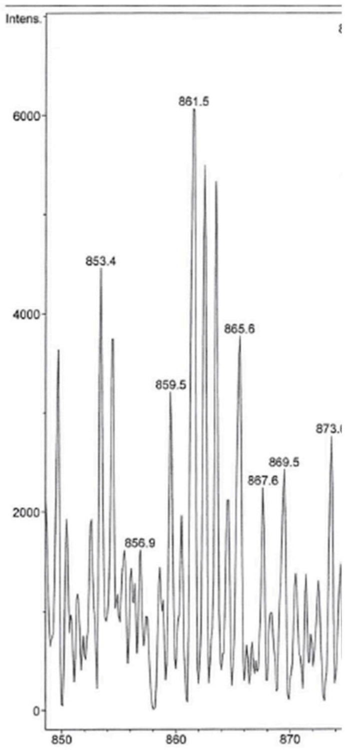

Figure S58. ESI spectrum of disubstituted EDTA alkyne **17.**

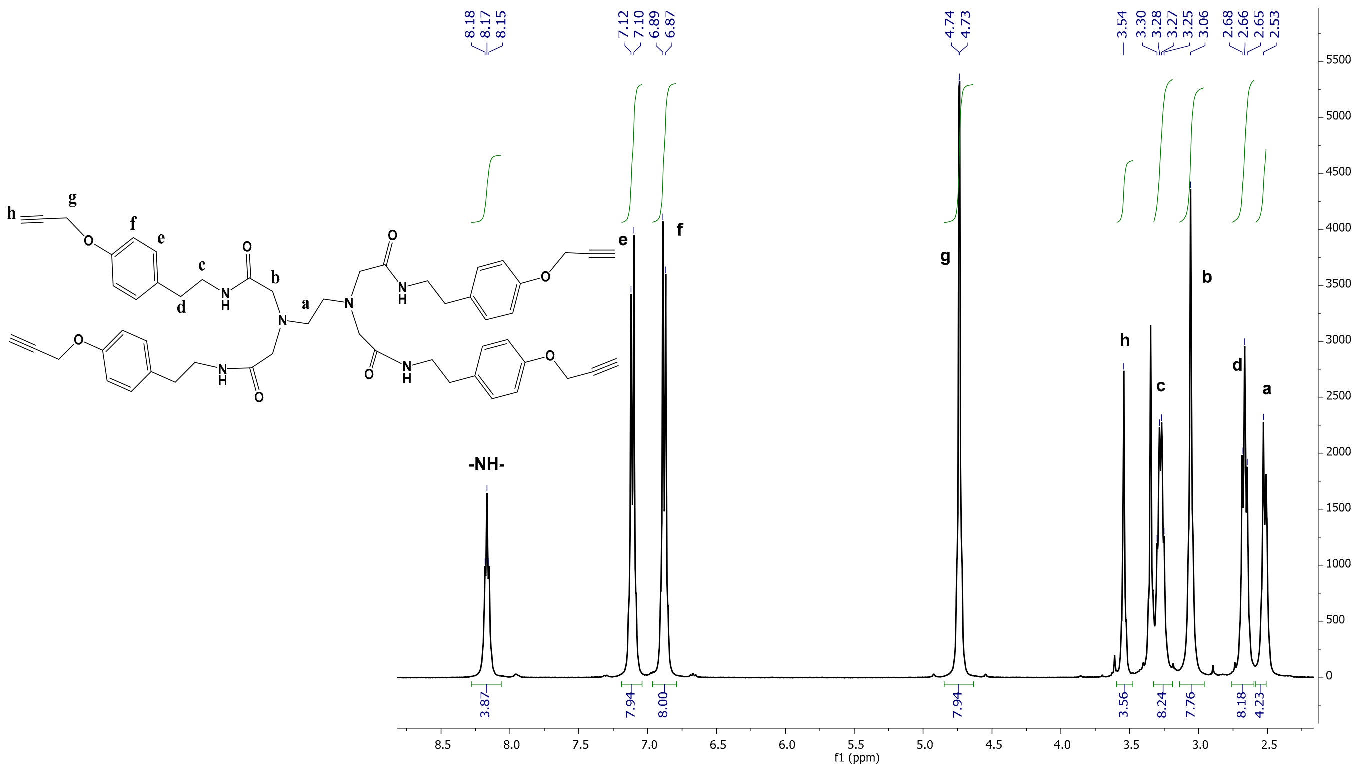

Figure S59. ^{1}H-NMR spectrum of tetrasubstituted EDTA G0-alkyne **18** in DMSO-*d*$_6$.

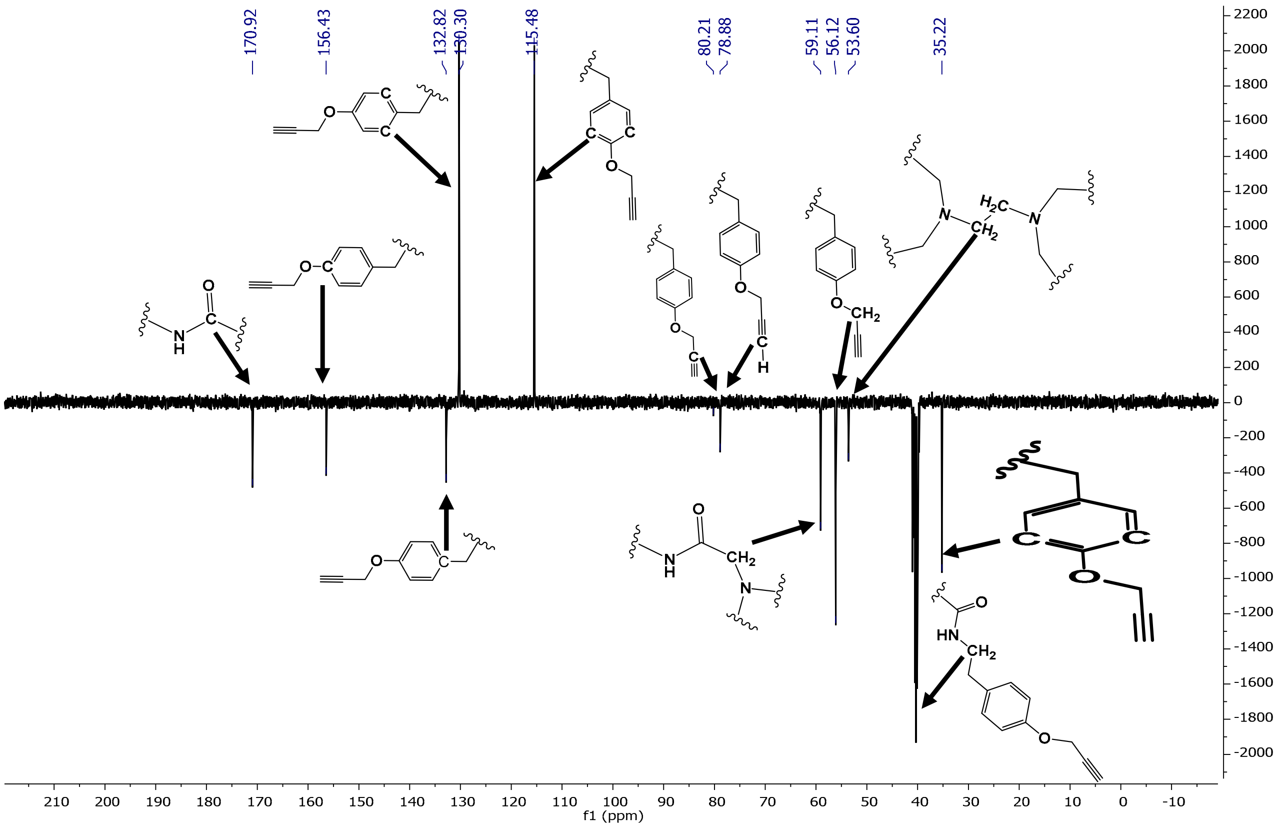

Figure S60. ^{13}C-NMR spectrum of tetrasubstituted EDTA G0-alkyne **18** in DMSO-*d*$_6$.

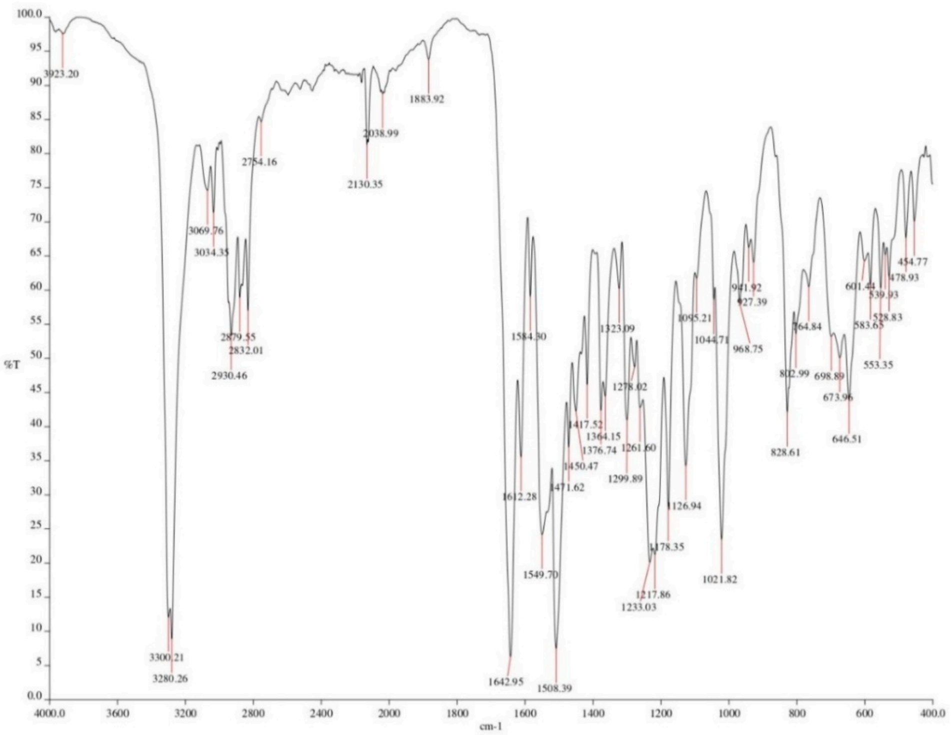

Figure S61. IR spectrum of tetrasubstituted EDTA G0-alkyne **18**.

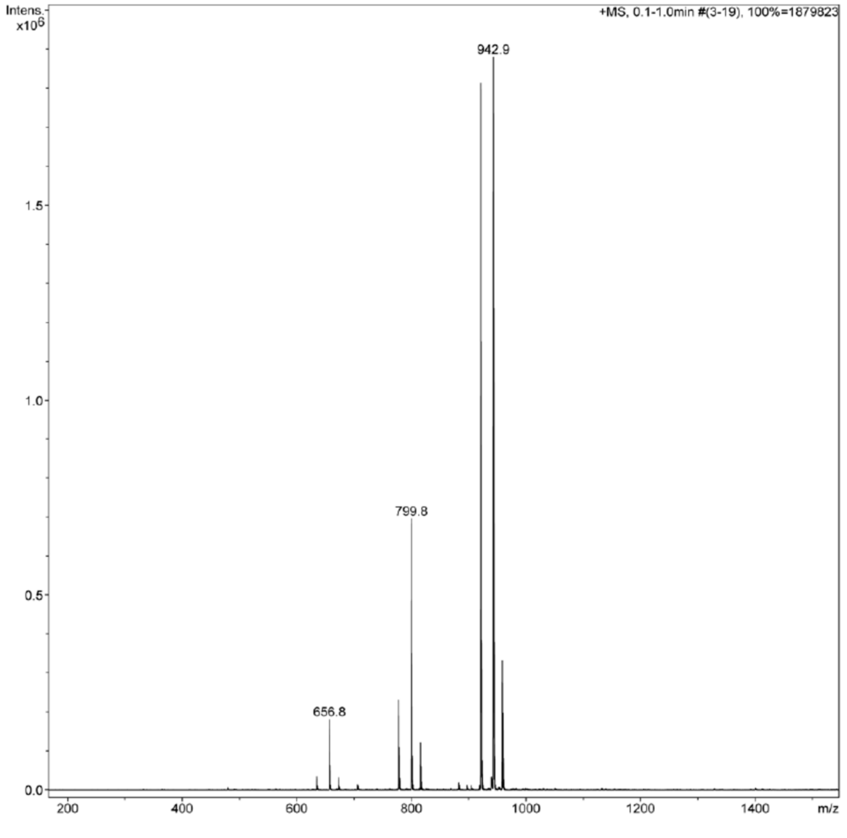

Figure S62. ESI spectrum of tetrasubstituted EDTA G0-alkyne **18**.

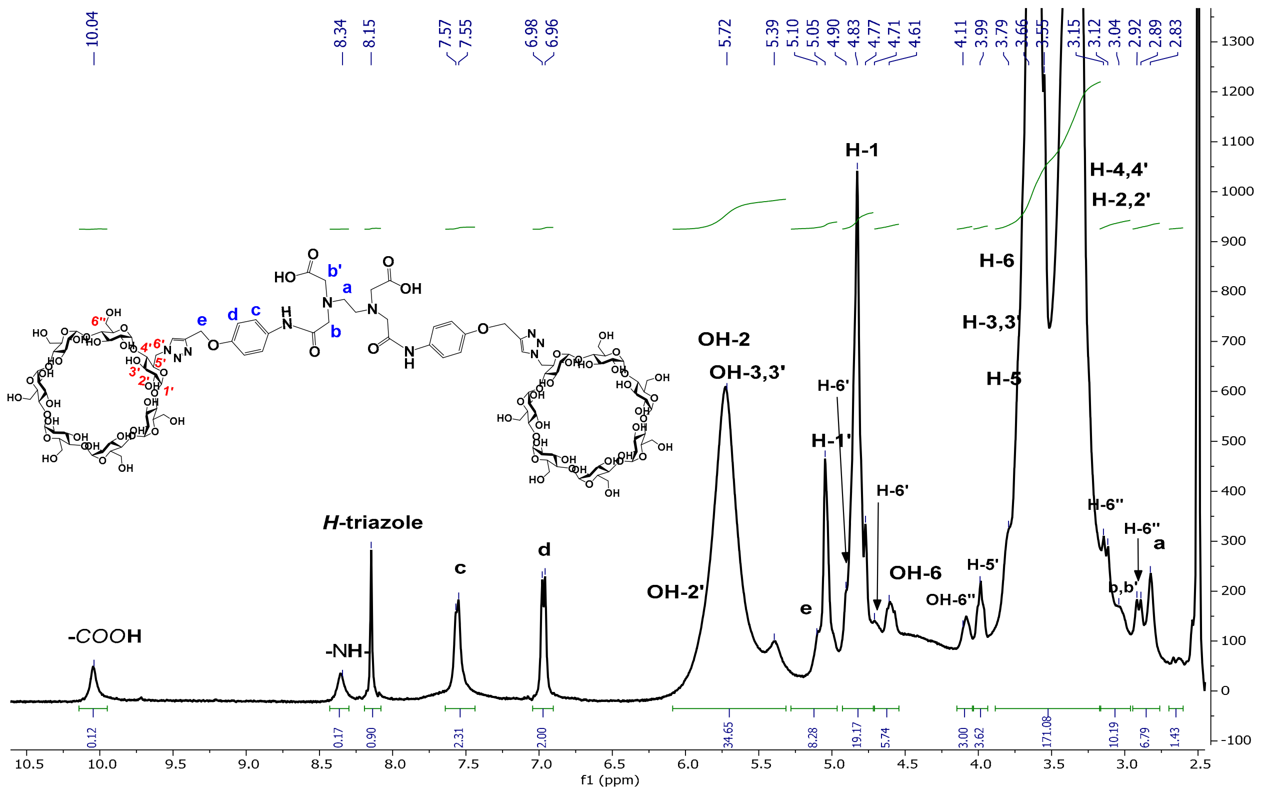

Figure S63. ^{1}H-NMR spectrum of dendritic EDTA2PhCD (**A**) in DMSO-d_6.

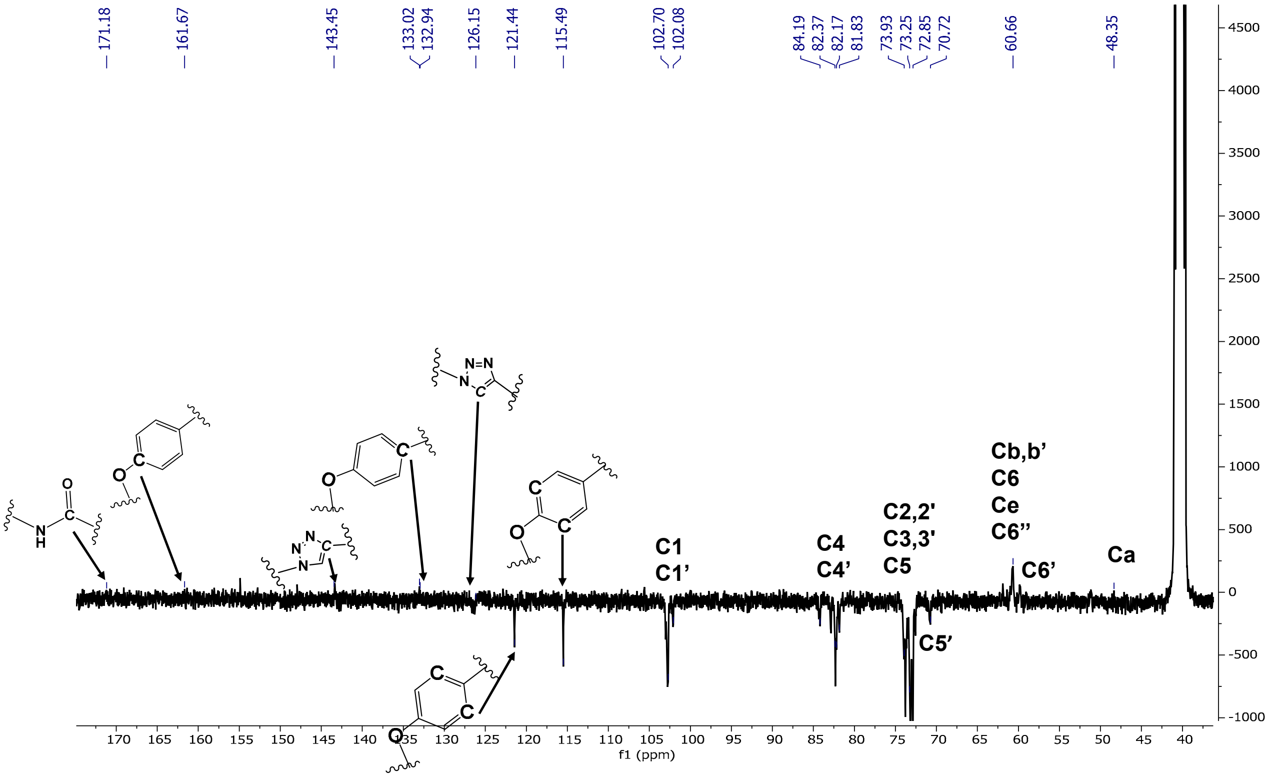

Figure S64. ^{13}C-NMR spectrum of dendritic EDTA2PhCD (**A**) in DMSO-d_6.

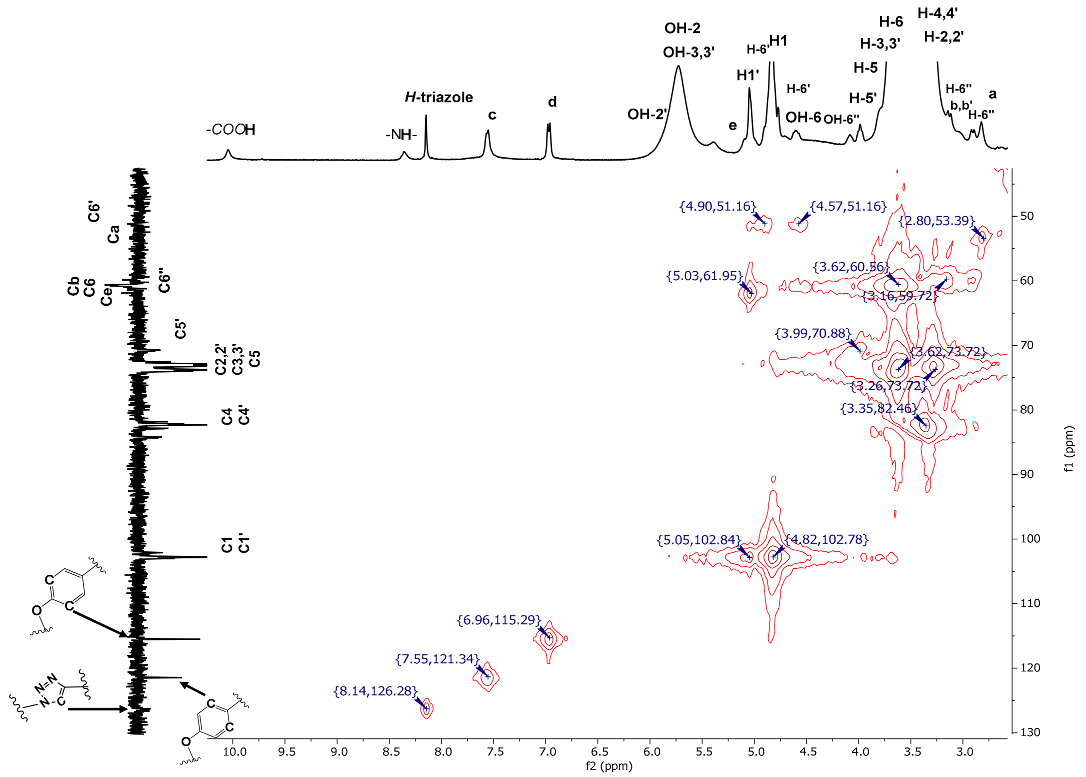

Figure S65. 2D NMR HMQC spectrum of dendritic EDTA2PhCD (**A**) in DMSO-d_6.

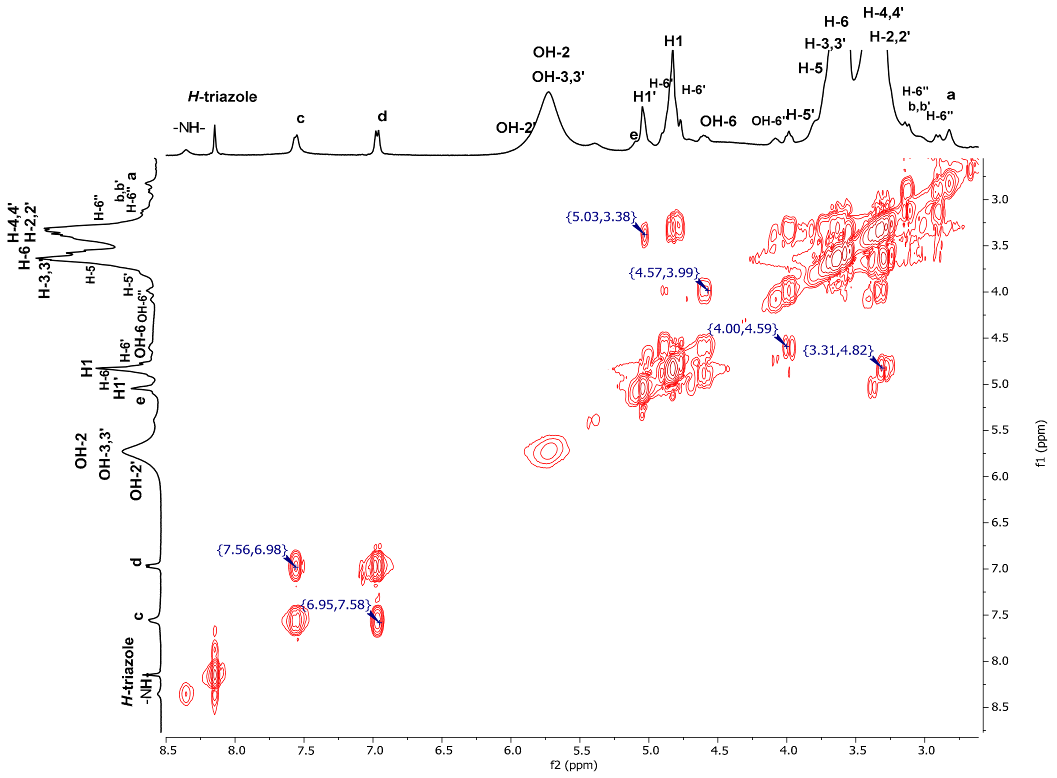

Figure S66. 2D NMR COSY spectrum of dendritic EDTA2PhCD (**A**) in DMSO-d_6.

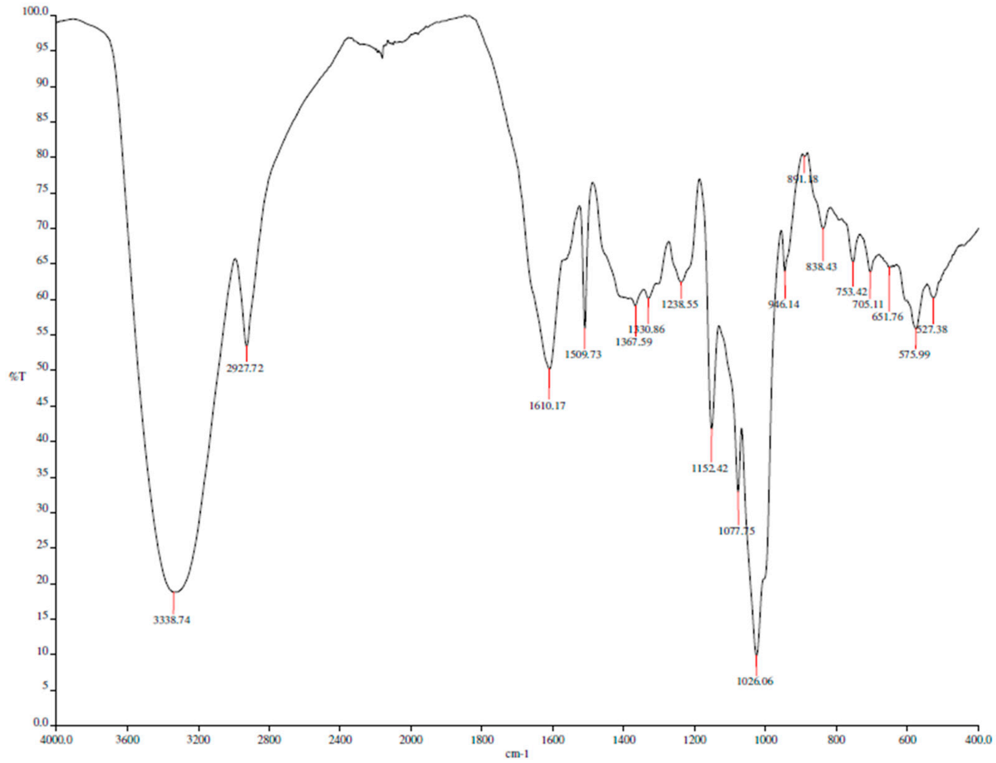

Figure S67. IR spectrum of dendritic EDTA2PhCD (**A**).

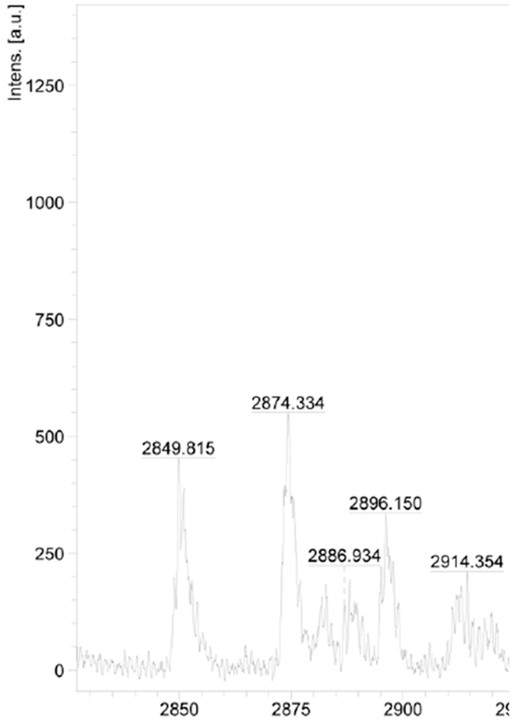

Figure S68. MALDI-TOF spectrum of dendritic EDTA2PhCD (**A**).

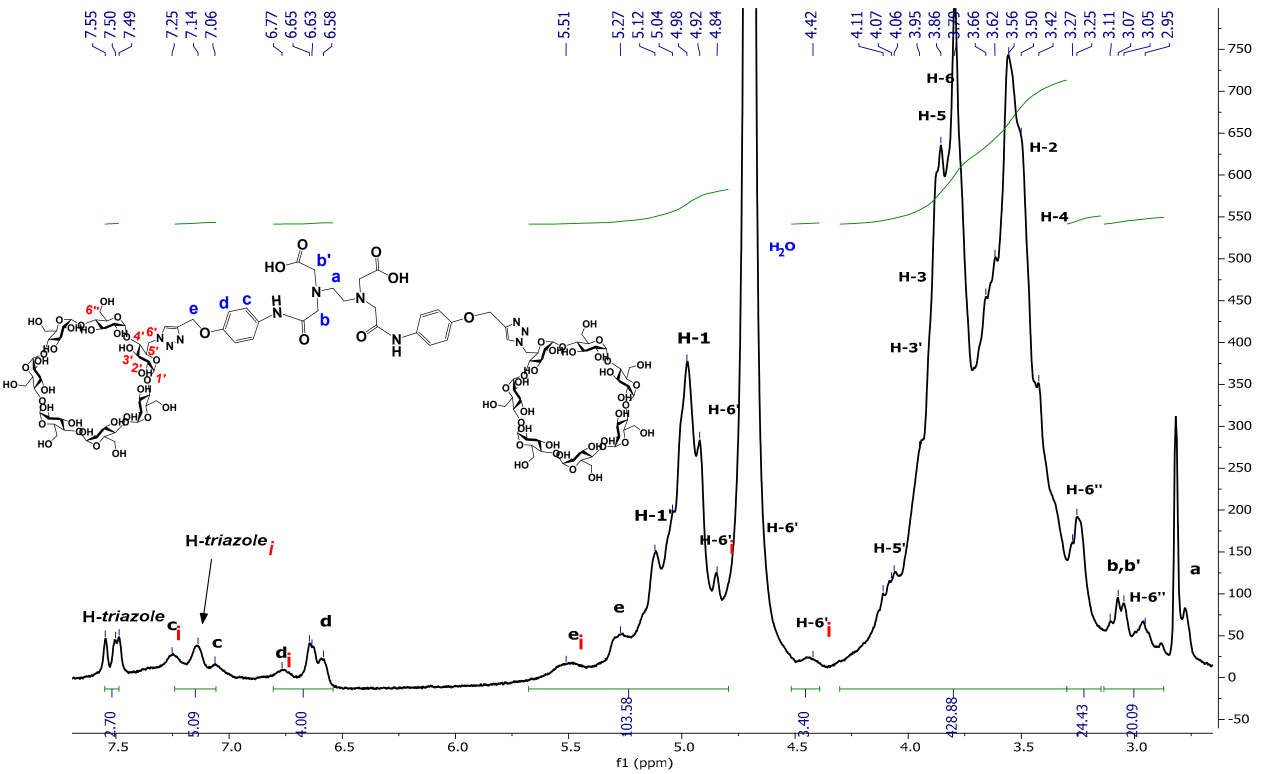

Figure S69. ^{1}H-NMR spectrum of dendritic EDTA2PhCD (**A**) in D_2O.

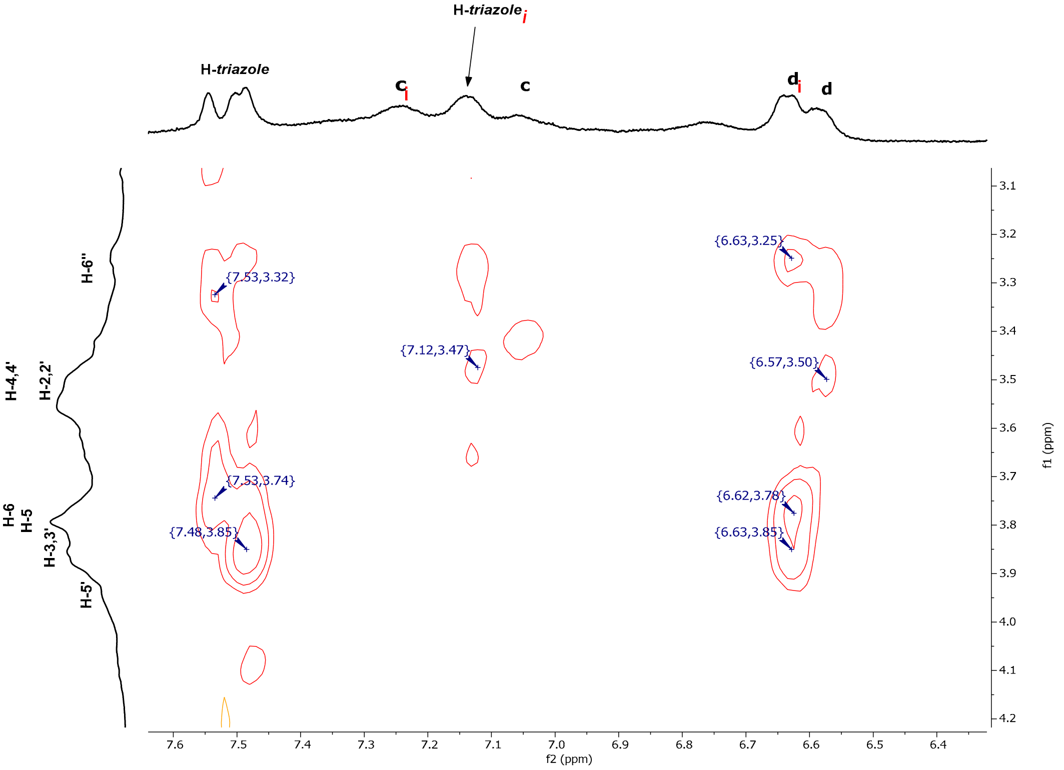

Figure S70. 2D NMR NOESY spectrum of EDTA2PhCD (**A**) in D_2O.

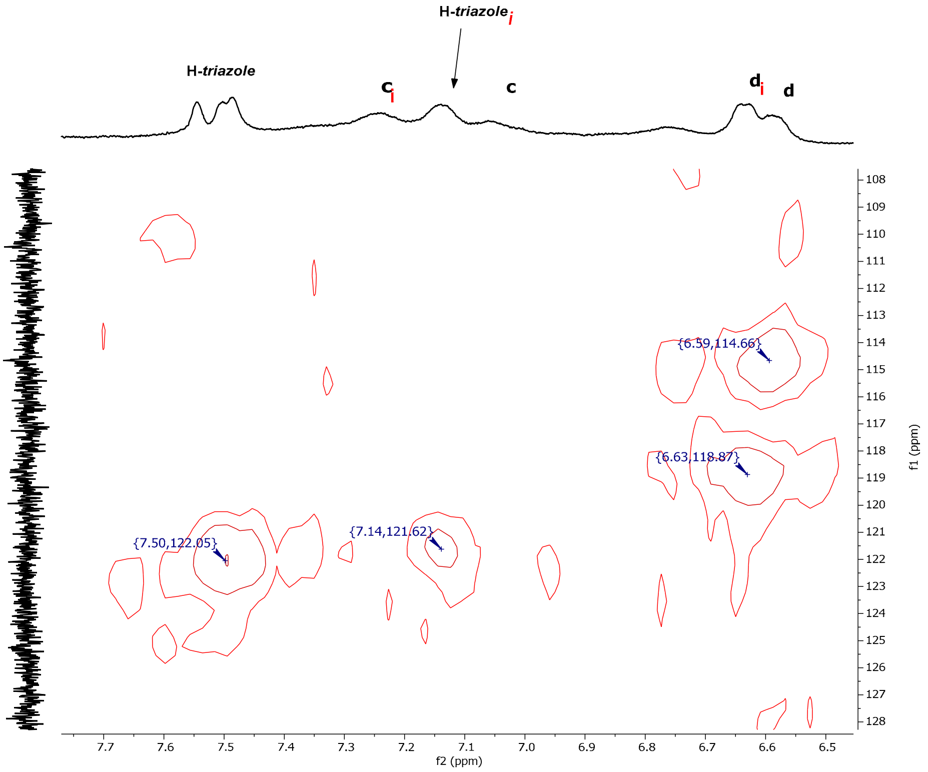

Figure S71. 2D NMR HMQC spectrum of EDTA2PhCD (**A**) in D_2O.

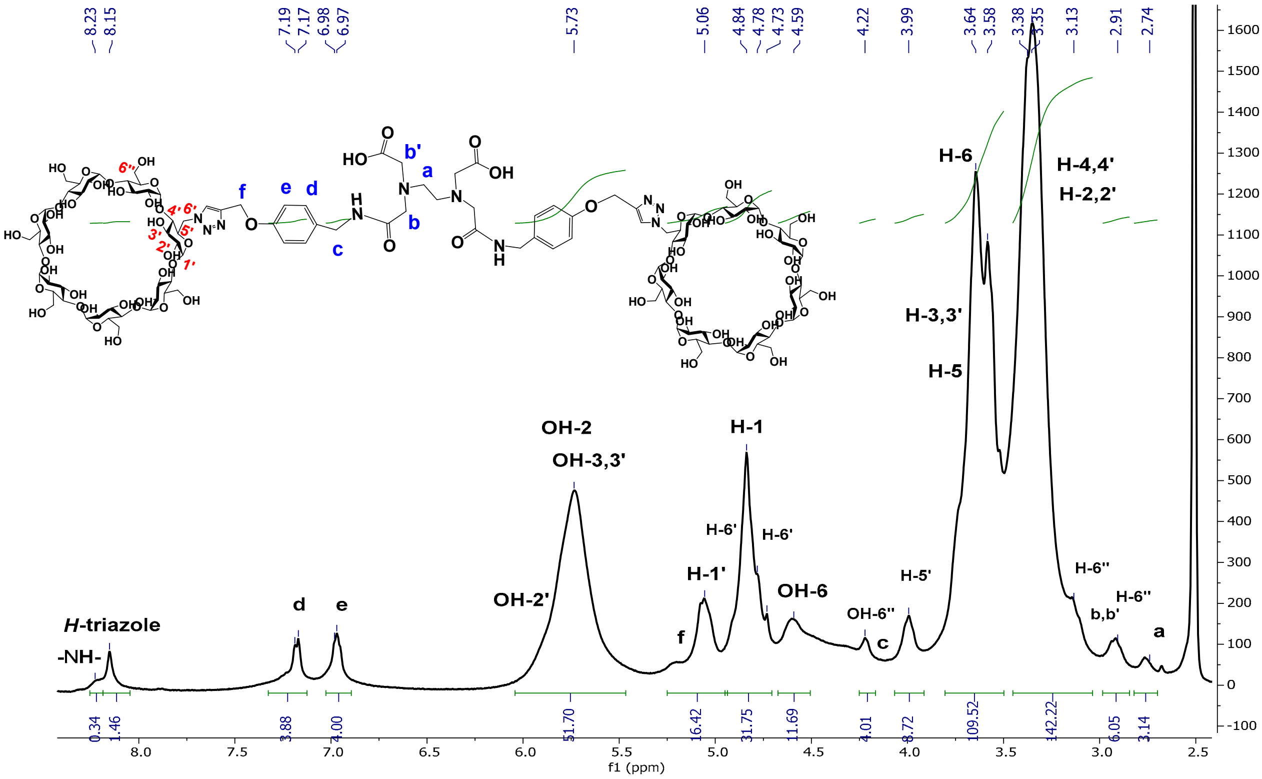

Figure S72. ^{1}H-NMR spectrum of dendritic EDTA2BenCD (**B**) in DMSO-d_6.

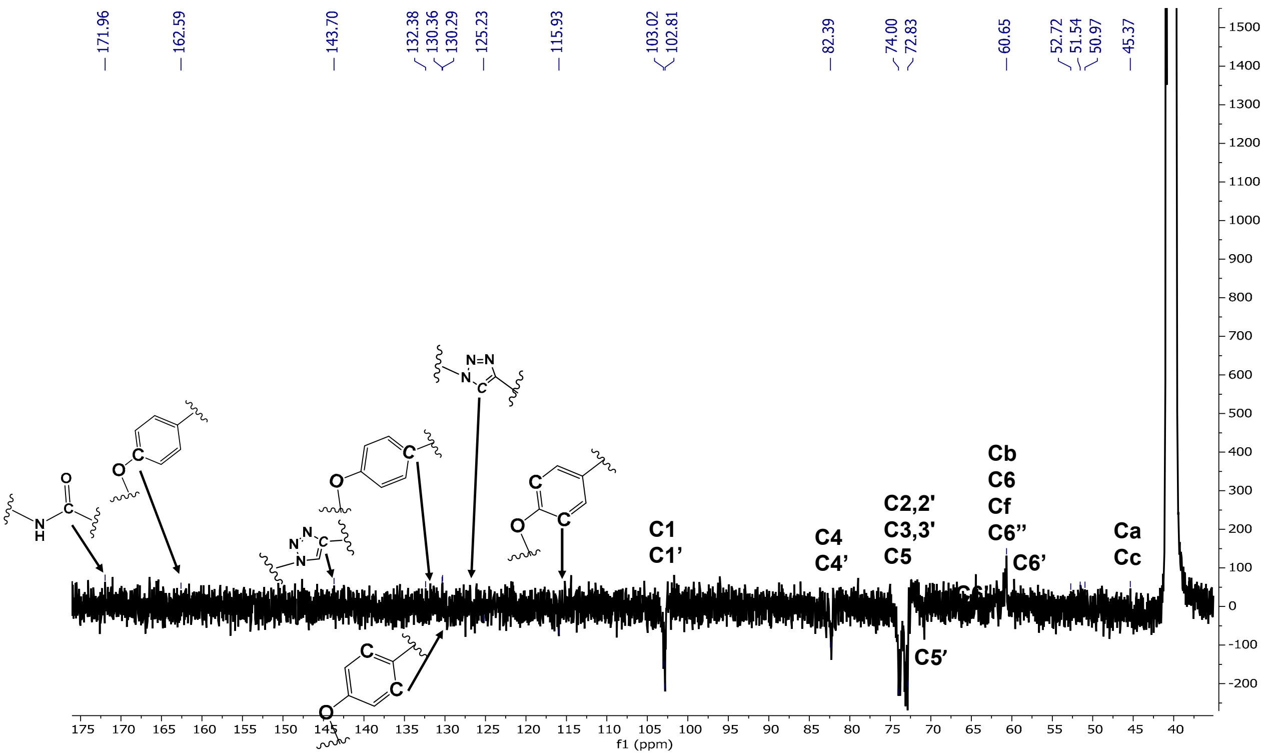

Figure S73. ^{13}C-NMR spectrum of dendritic EDTA2BenCD (**B**) in DMSO-d_6.

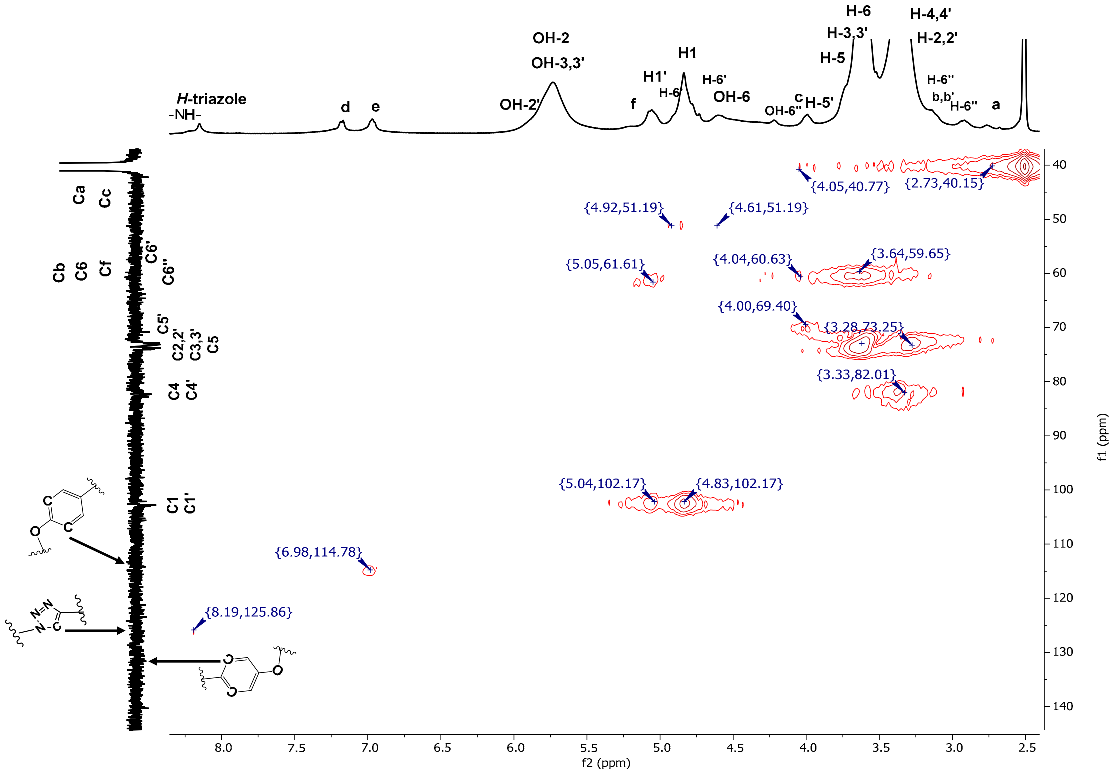

Figure S74. 2D NMR HMQC spectrum of EDTA2BenCD (**B**) in DMSO-d_6.

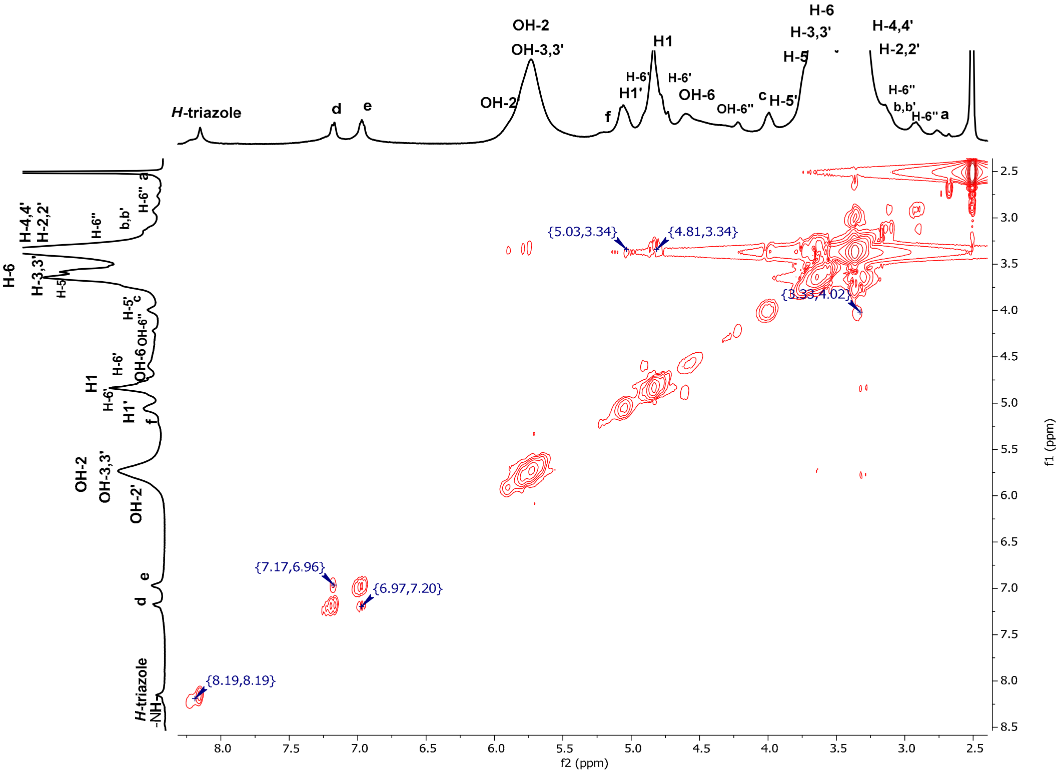

Figure S75. 2D NMR COSY spectrum of EDTA2BenCD (**B**) in DMSO-d_6.

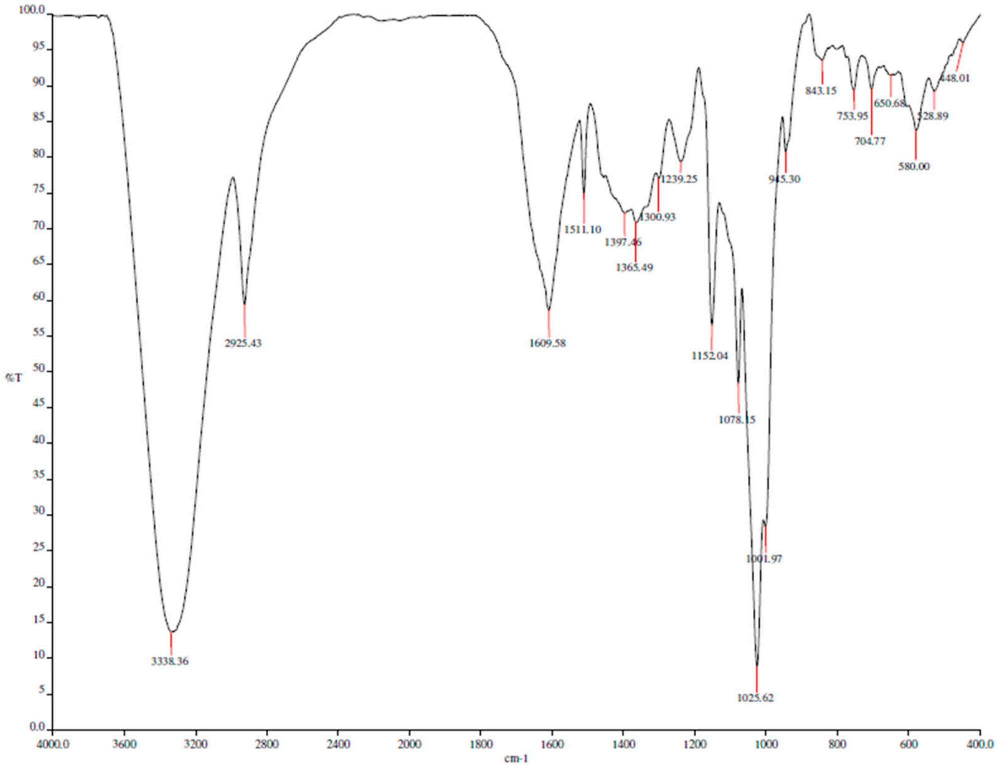

Figure S76. IR spectrum of dendritic EDTA2BenCD (**B**).

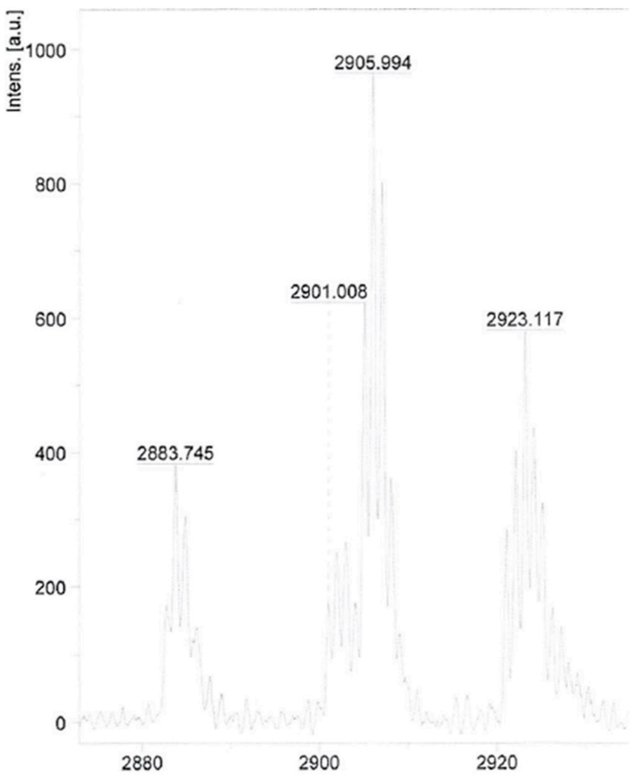

Figure S77. MALDI-TOF spectrum of dendritic EDTA2BenCD (**B**).

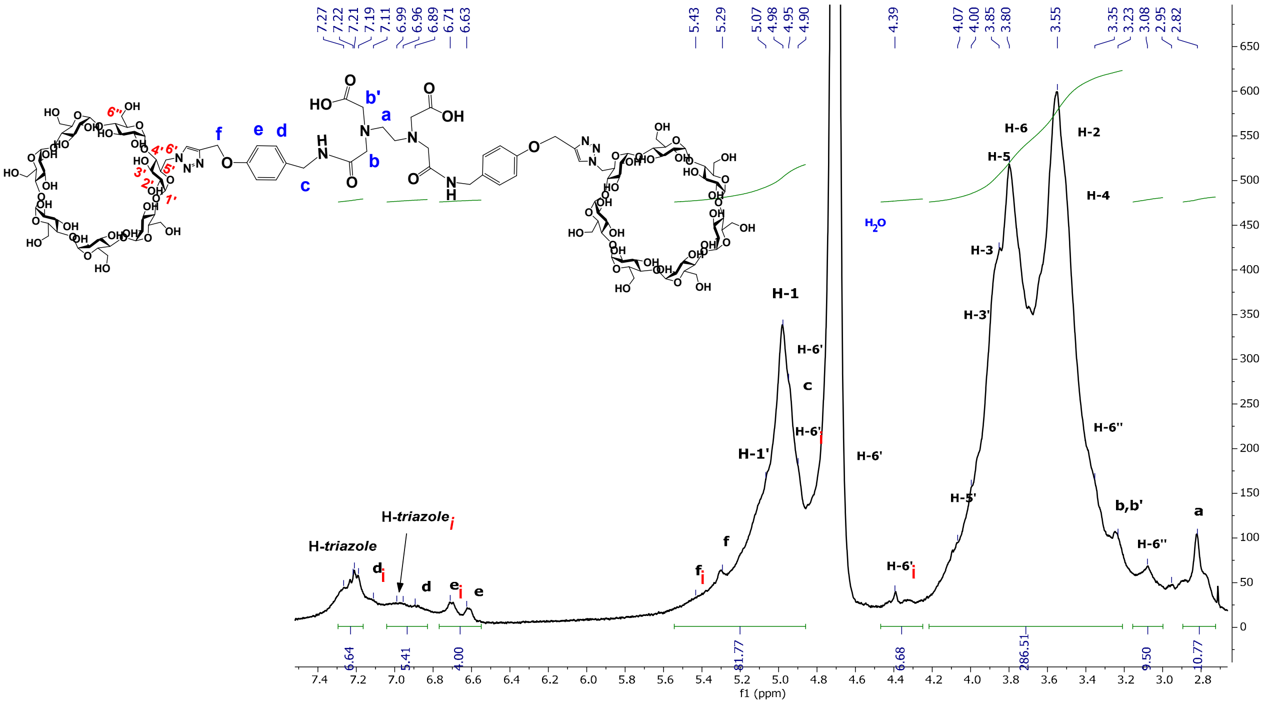

Figure S78. ^{1}H-NMR spectrum of dendritic EDTA2BenCD (**B**) in D_2O.

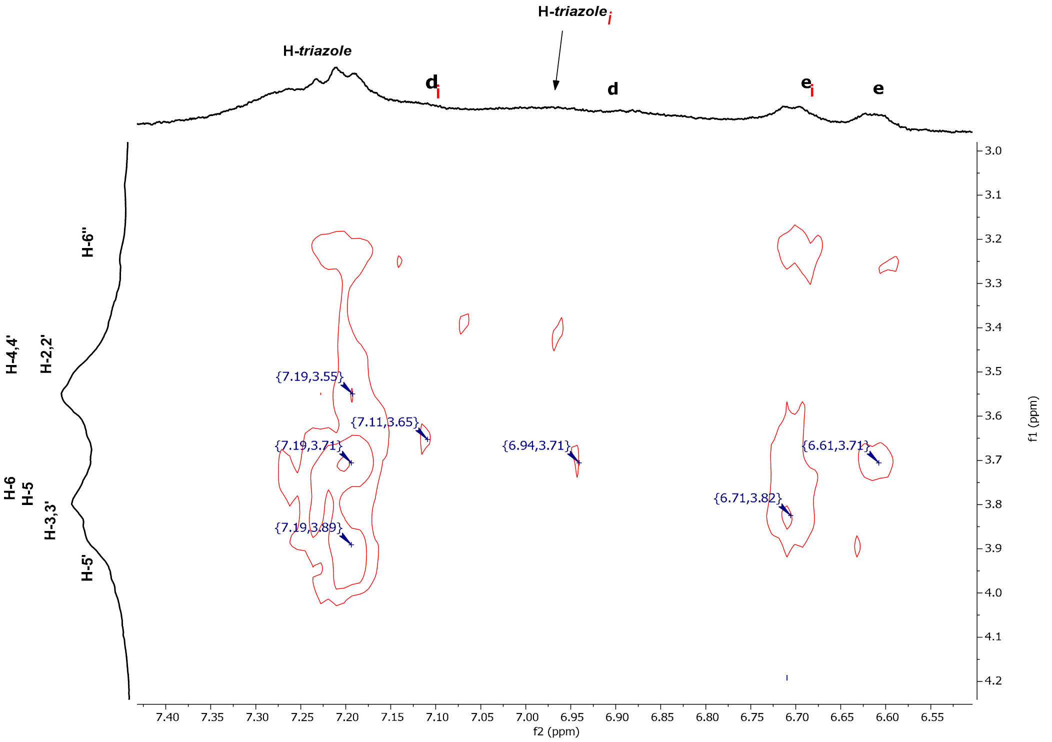

Figure S79. 2D NMR NOESY spectrum of EDTA2BenCD (**B**) in D_2O.

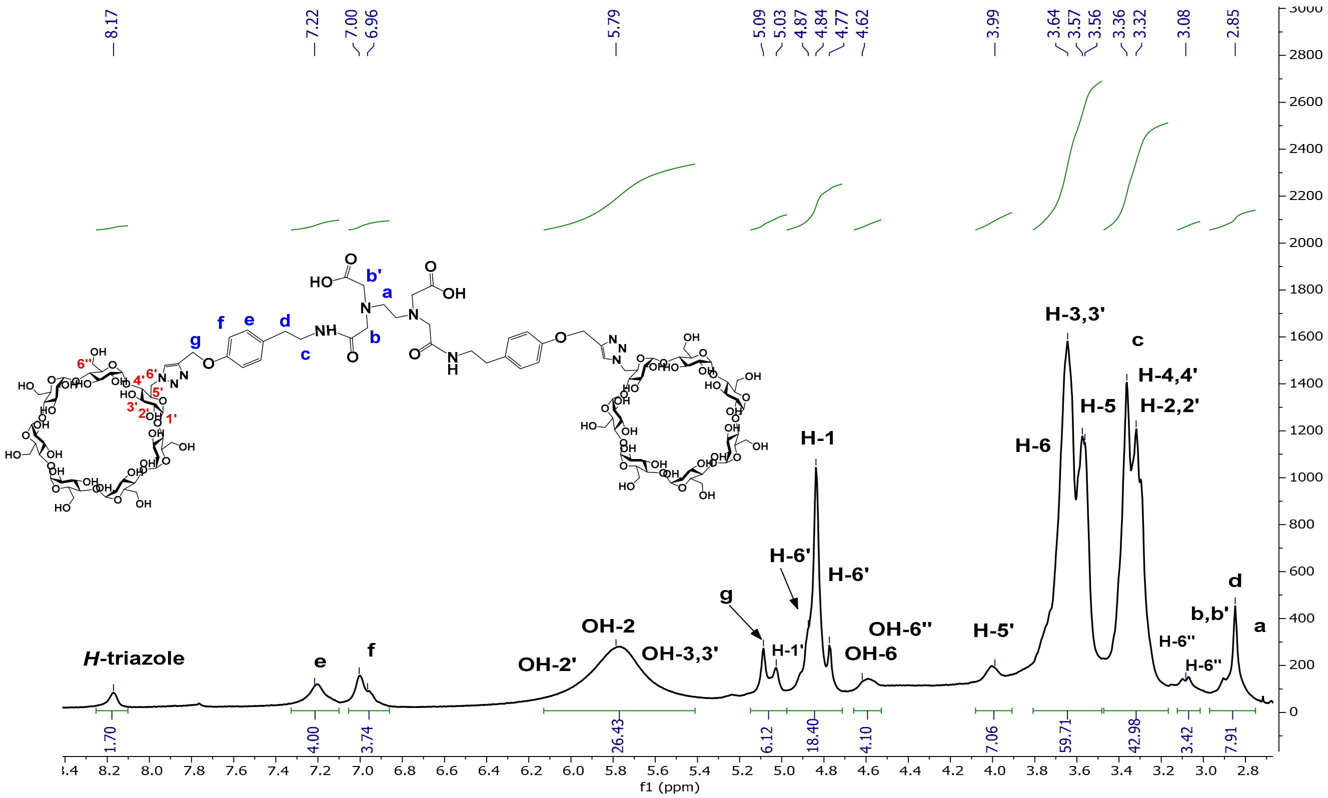

Figure S80. ^{1}H-NMR spectrum of dendritic EDTA2TyrCD (**C**) in DMSO-d_6.

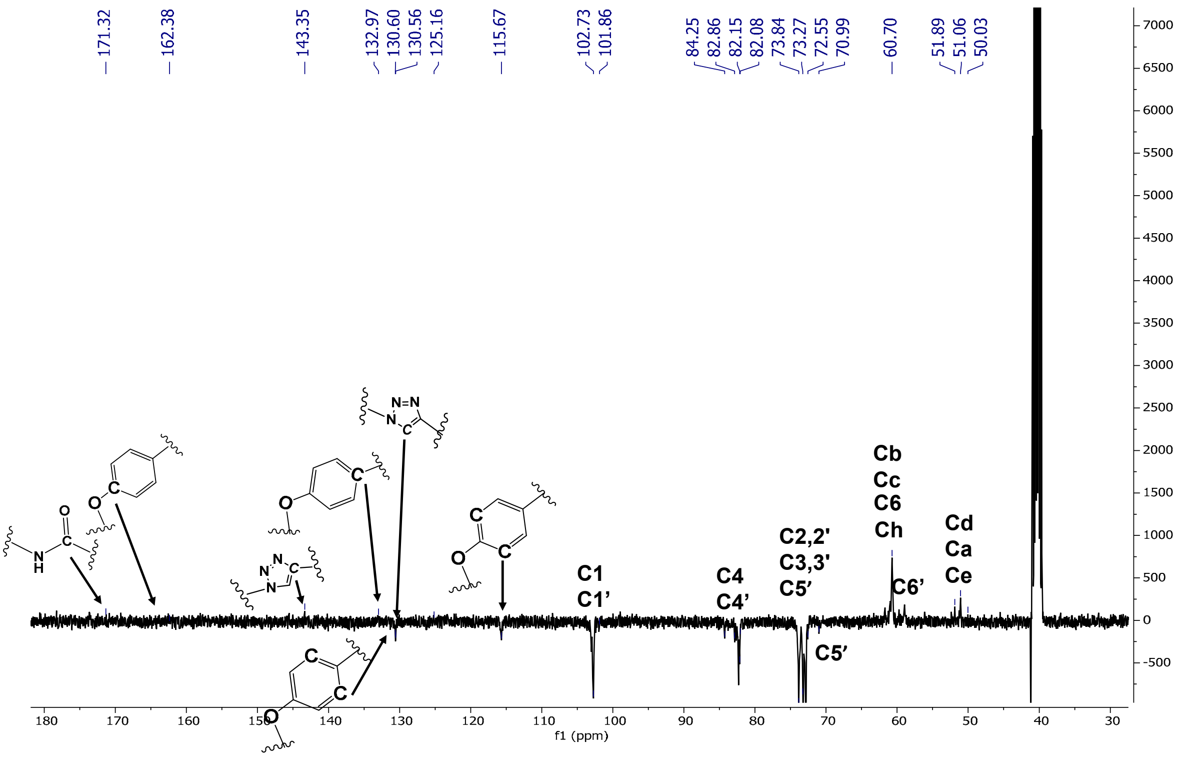

Figure S81. ^{13}C-NMR spectrum of dendritic EDTA2TyrCD (**C**) in DMSO-*d₆*.

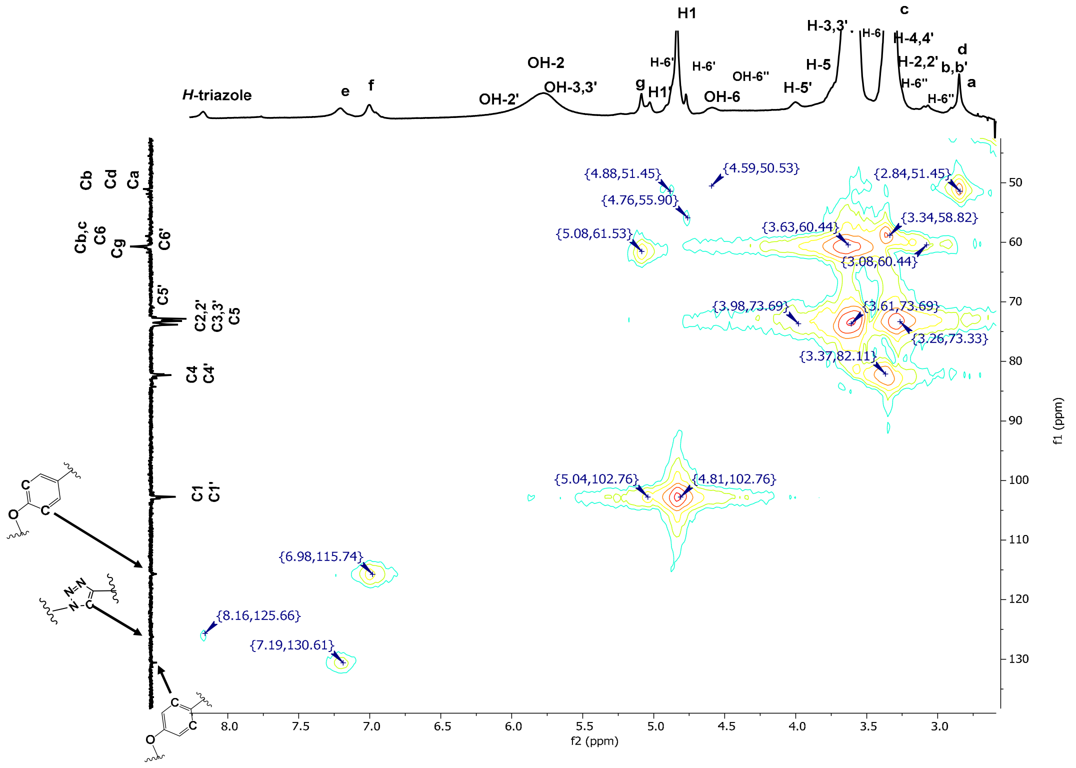

Figure S82. 2D NMR HMQC spectrum of EDTA2TyrCD (**C**) in DMSO-d_6.

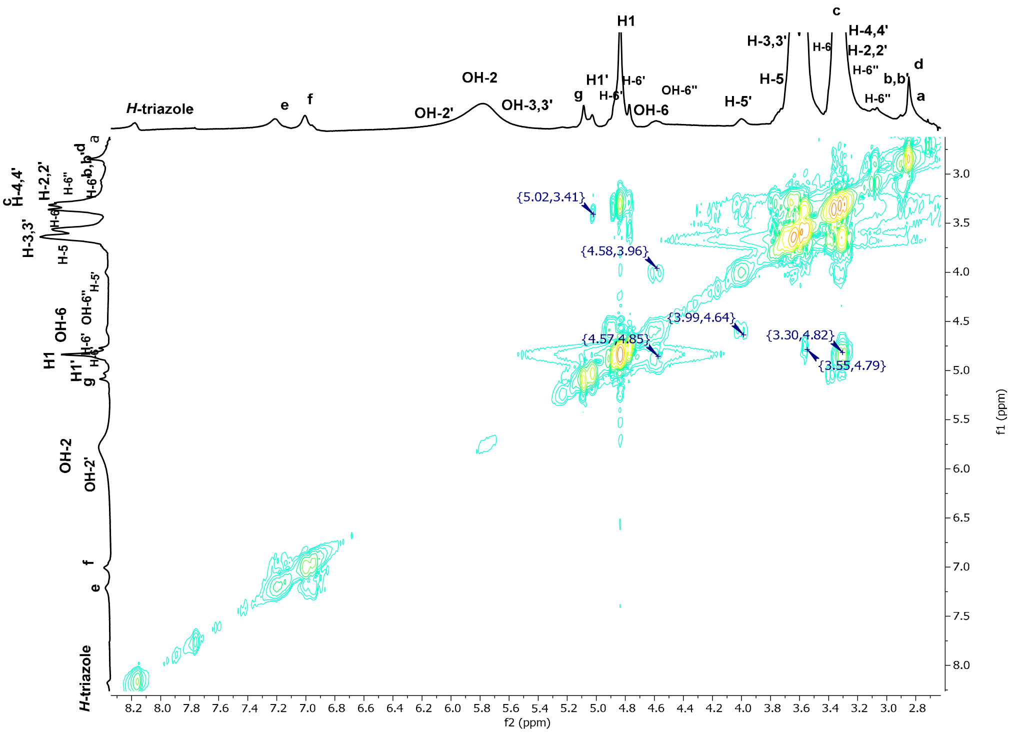

Figure S83. 2D NMR COSY spectrum of dendritic EDTA2TyrCD (**C**) in DMSO-d_6.

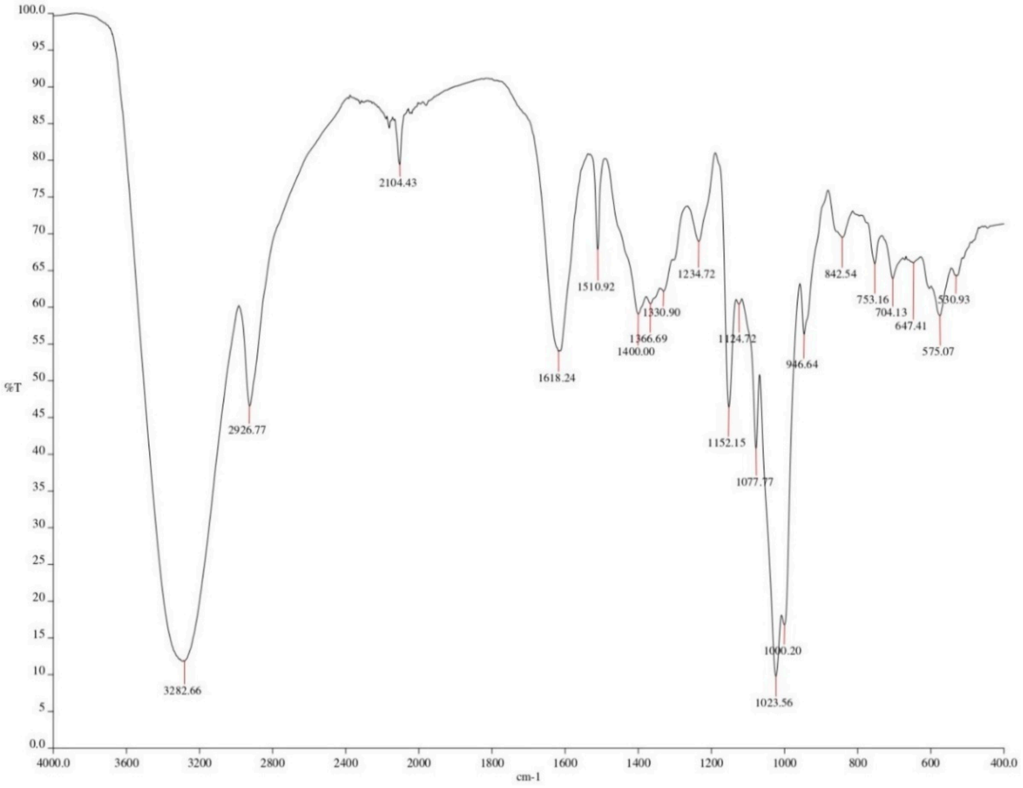

Figure S84. IR spectrum of dendritic EDTA2TyrCD (**C**).

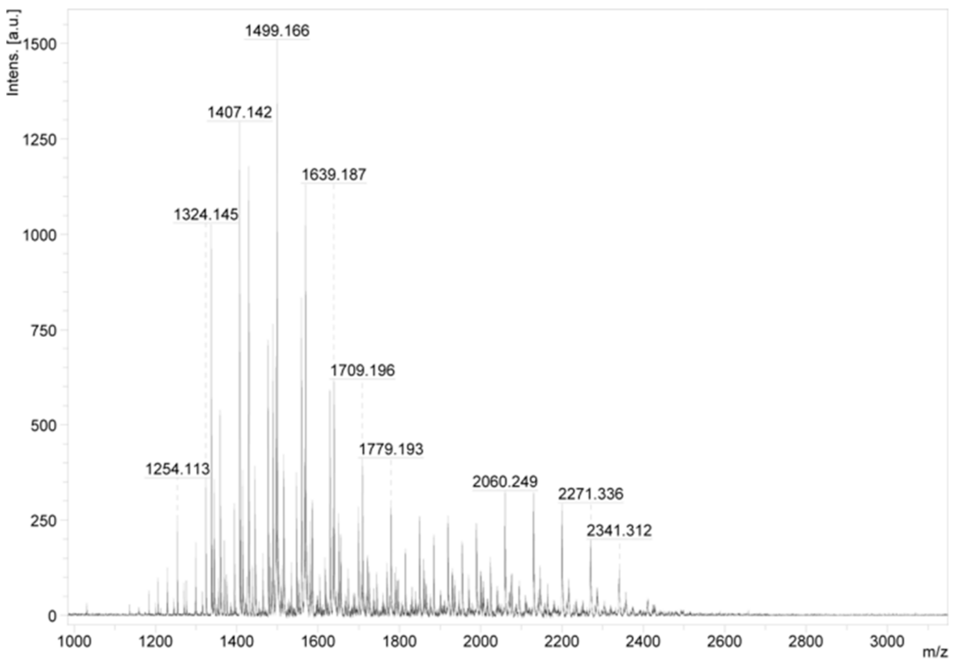

Figure S85. ESI-TOF spectrum of dendritic EDTA2TyrCD (**C**).

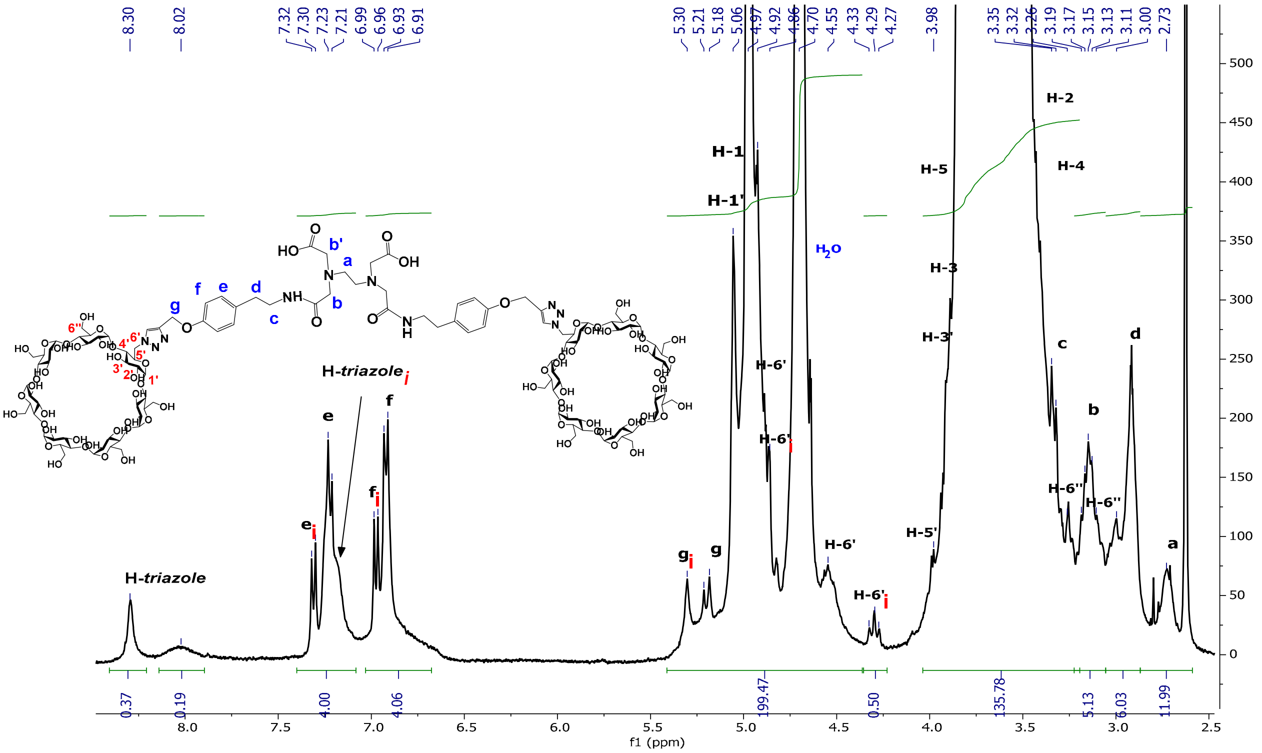

Figure S86. ¹H-NMR spectrum of dendritic EDTA2TyrCD (**C**) in D_2O.

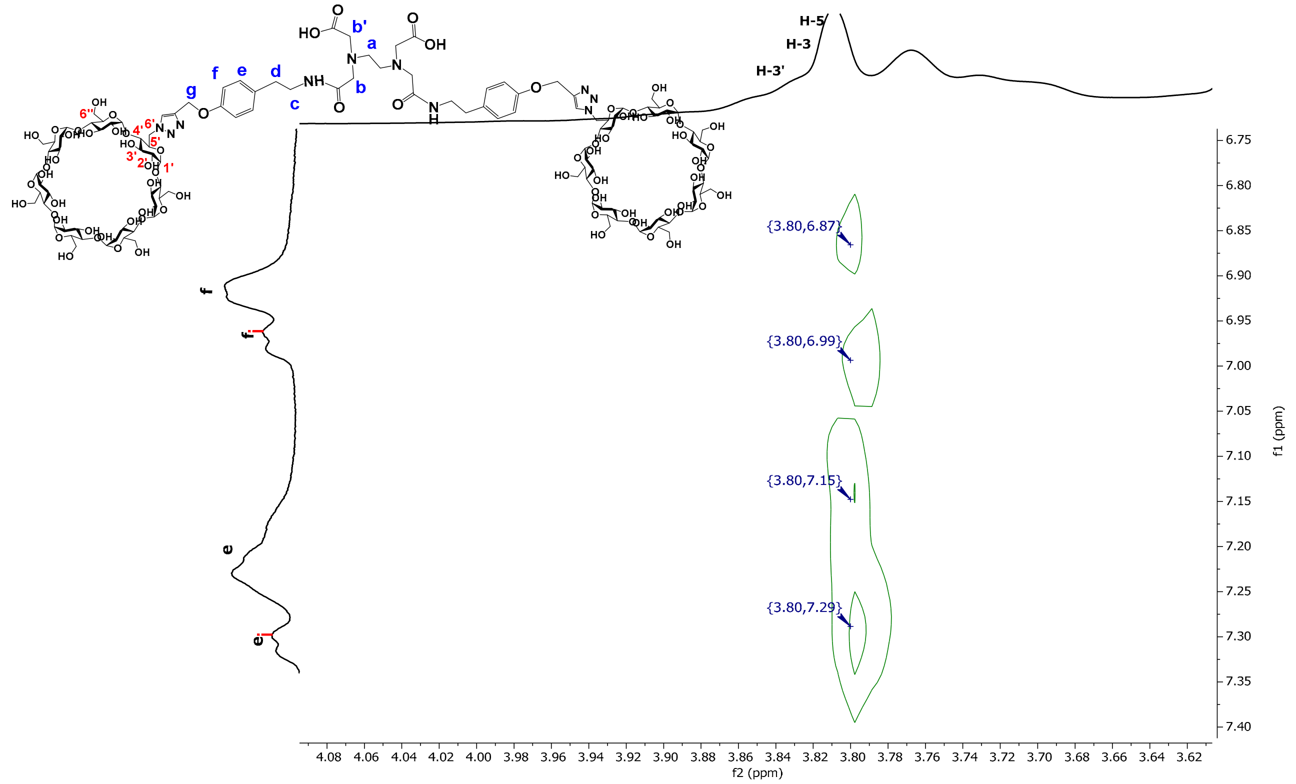

Figure S87. 2D NMR NOESY spectrum of dendritic EDTA2TyrCD (**C**) in D_2O.

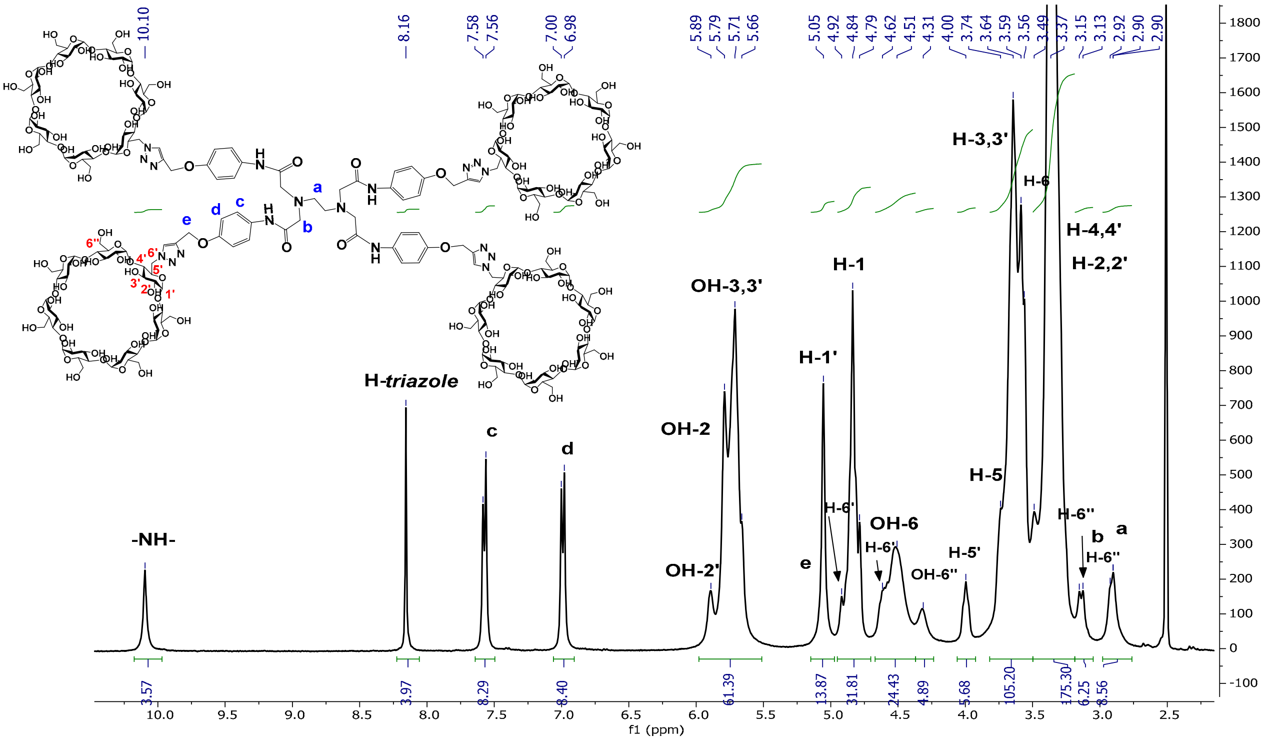

Figure S88. ^{1}H-NMR spectrum of EDTA4PhCD dendrimer (**D**) in DMSO-d_6.

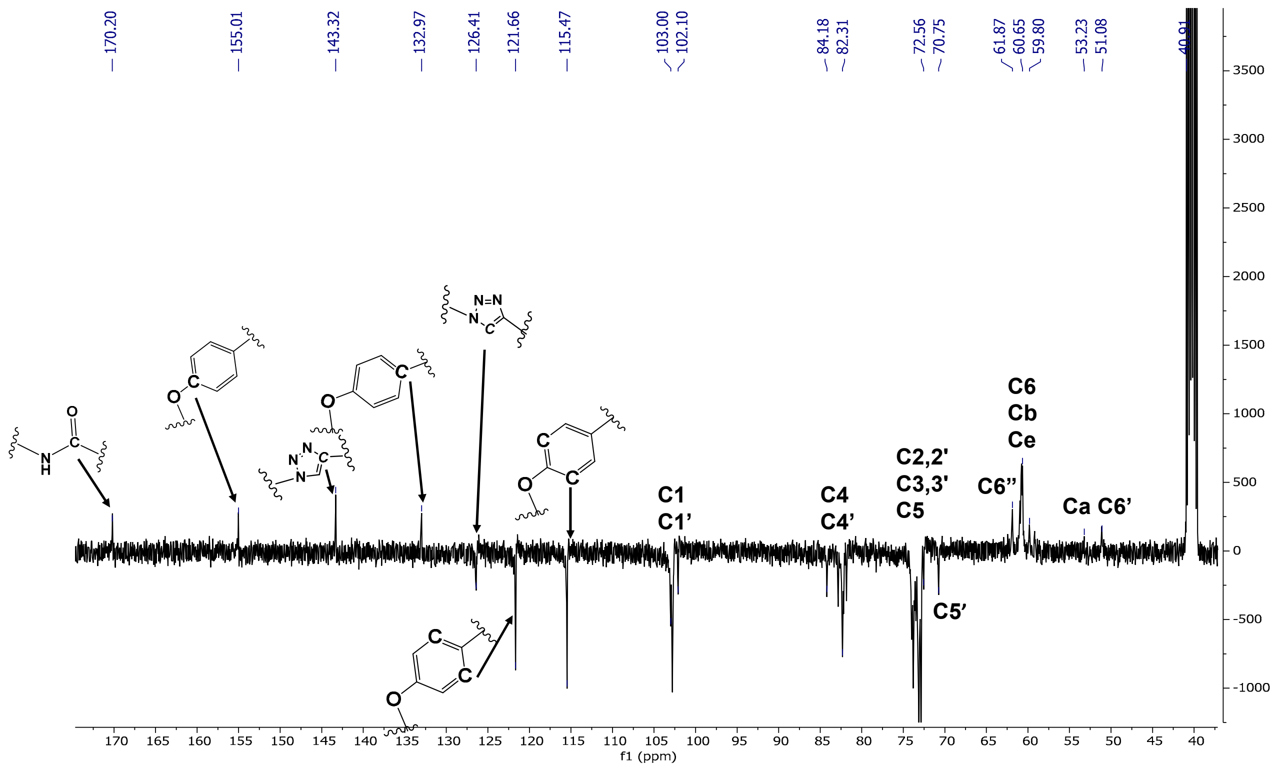

Figure S89. ^{13}C-NMR spectrum of EDTA4PhCD dendrimer (**D**) in DMSO-d_6.

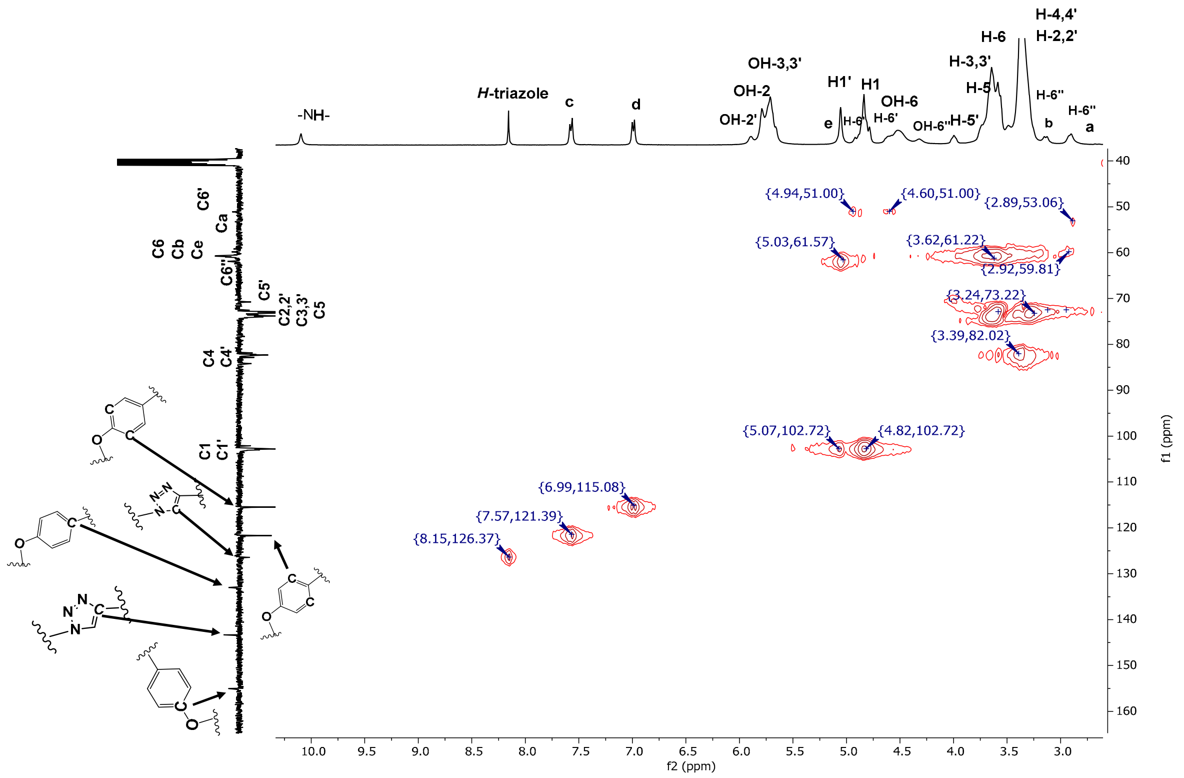

Figure S90. 2D NMR HMQC spectrum of EDTA4PhCD dendrimer (**D**) in DMSO-d_6.

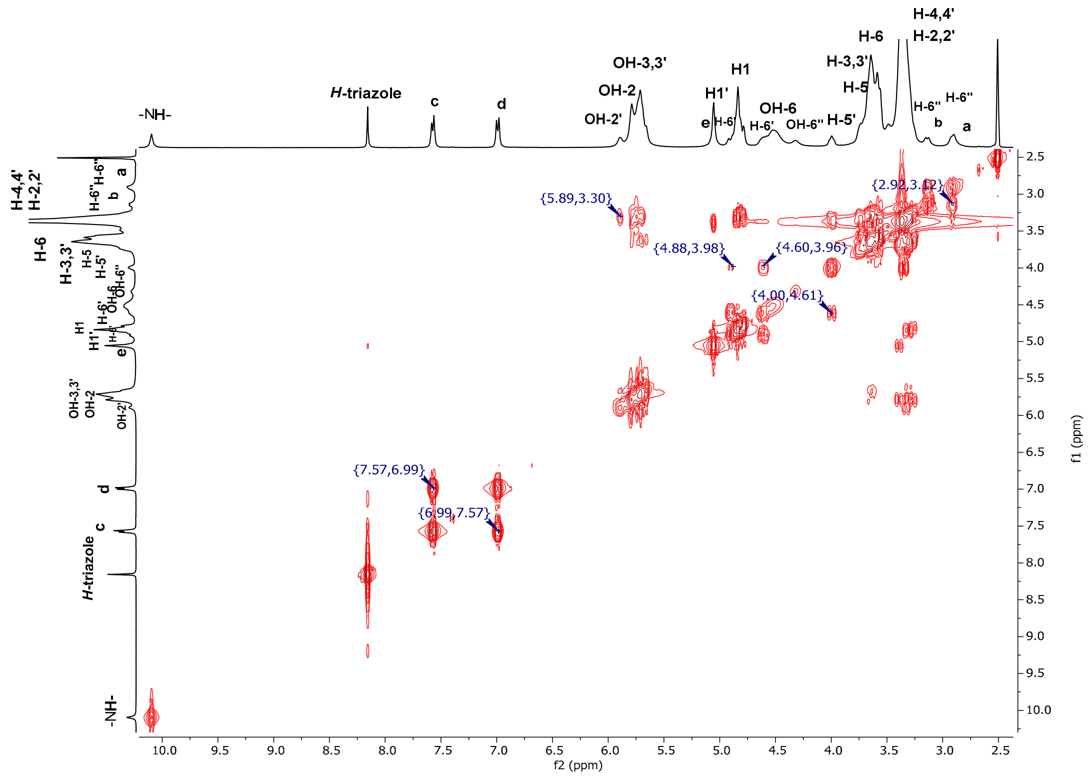

Figure S91. 2D NMR COSY spectrum of EDTA4PhCD dendrimer (**D**) in DMSO-d_6.

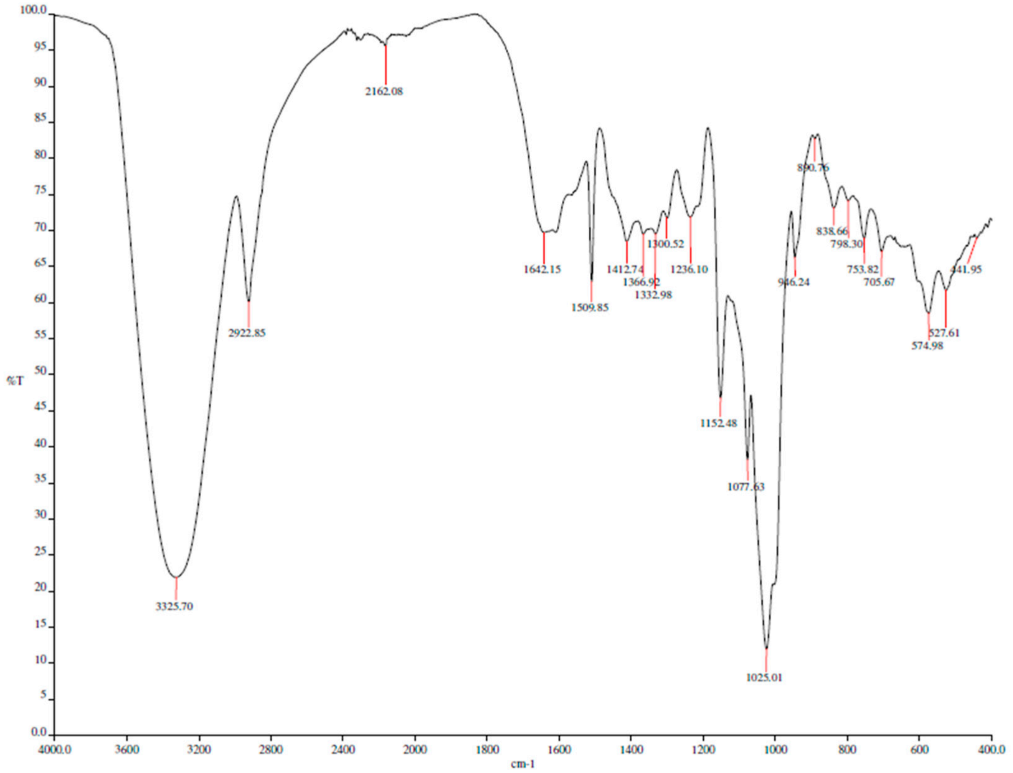

Figure S92. IR spectrum of EDTA4PhCD dendrimer (**D**).

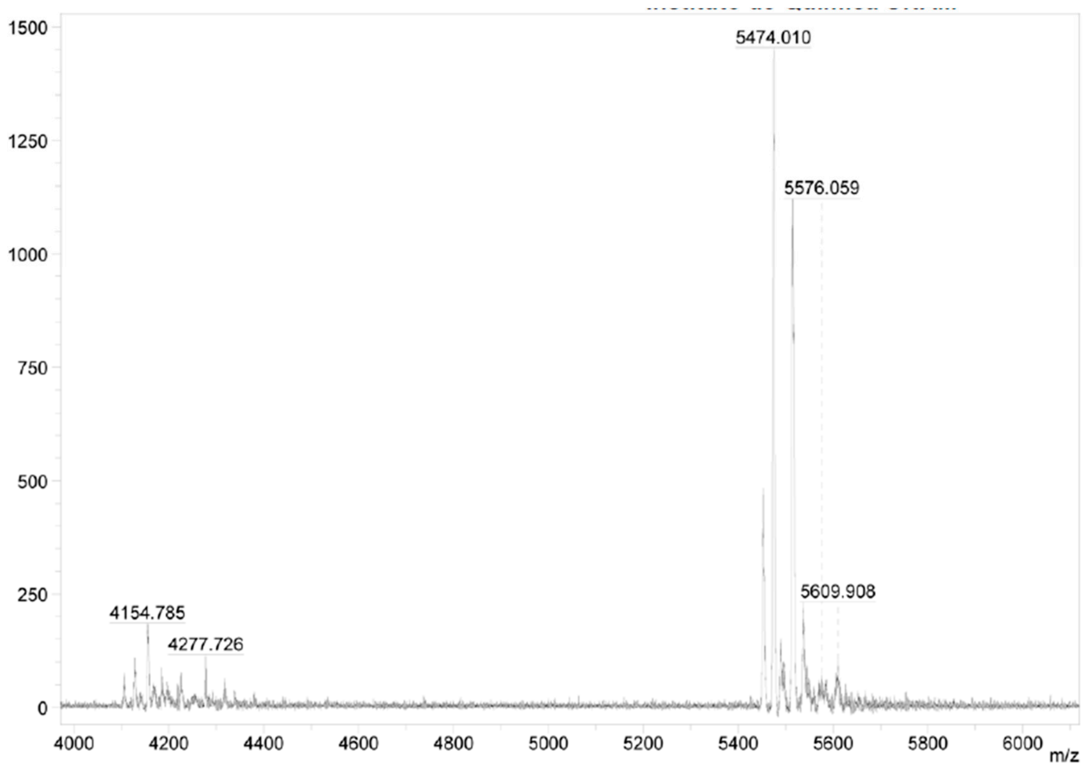

Figure S93. MALDI-TOF spectrum of EDTA4PhCD dendrimer (**D**).

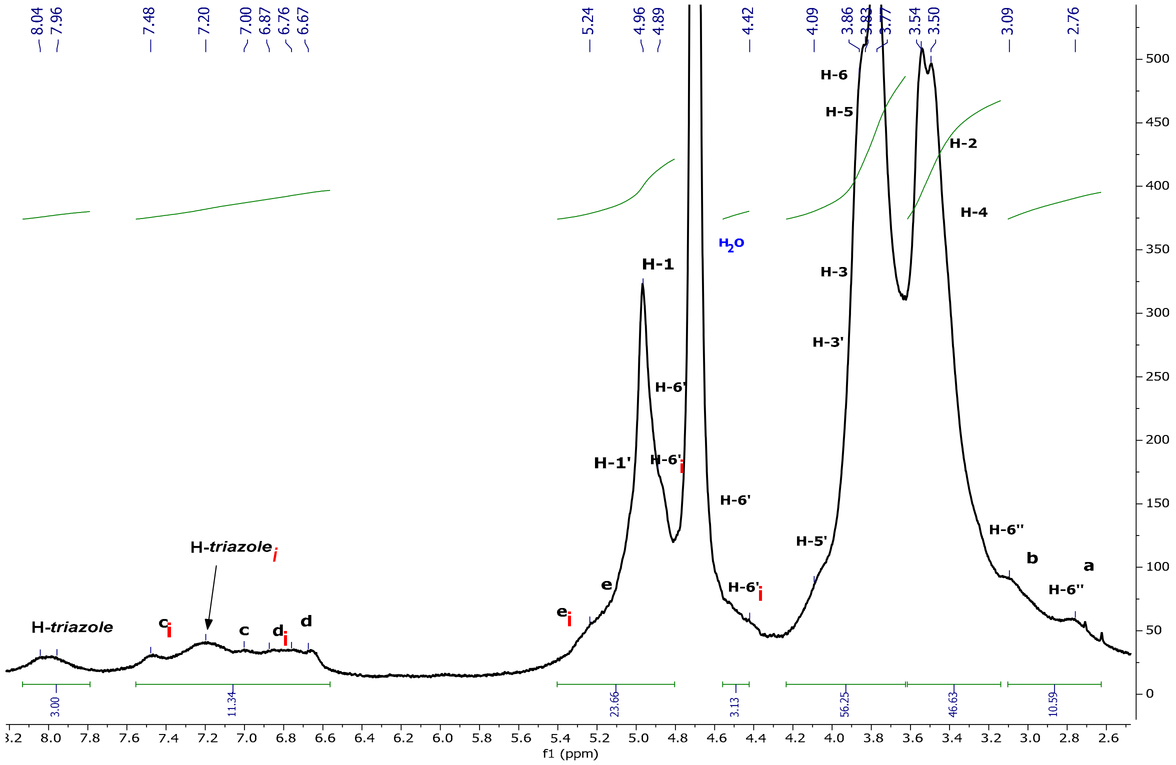

Figure S94. ^{1}H-NMR spectrum of EDTA4PhCD dendrimer (**D**) in D_2O.

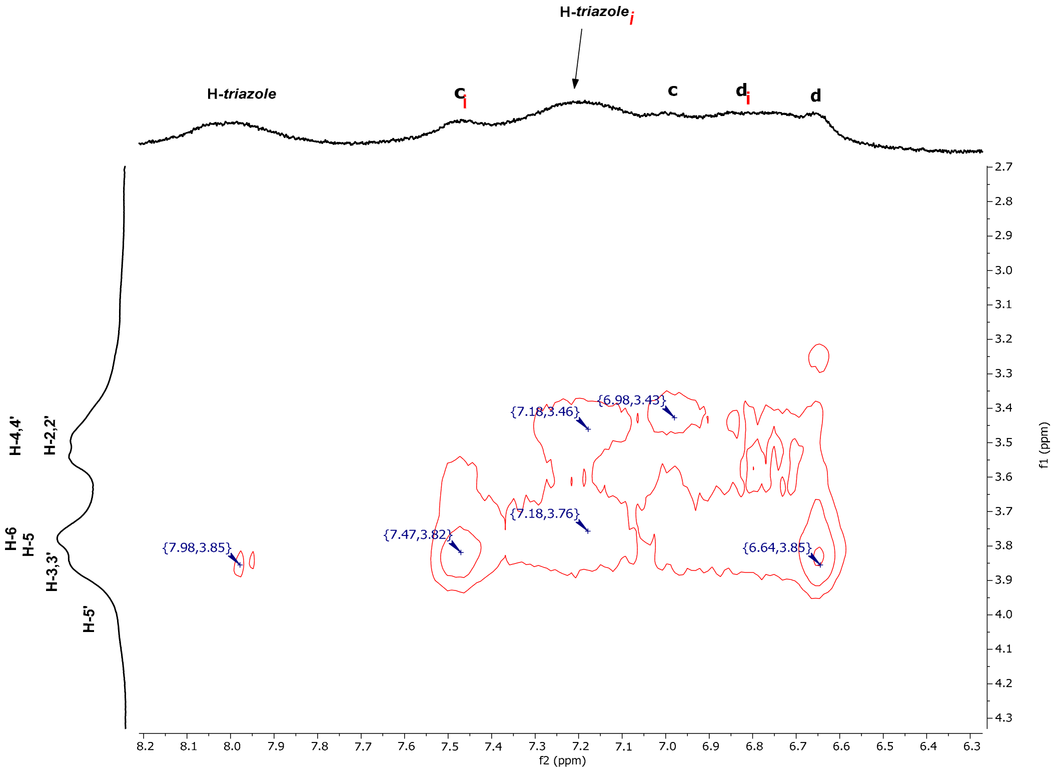

Figure S95. 2D NMR NOESY spectrum of EDTA4PhCD dendrimer (**D**) in D_2O.

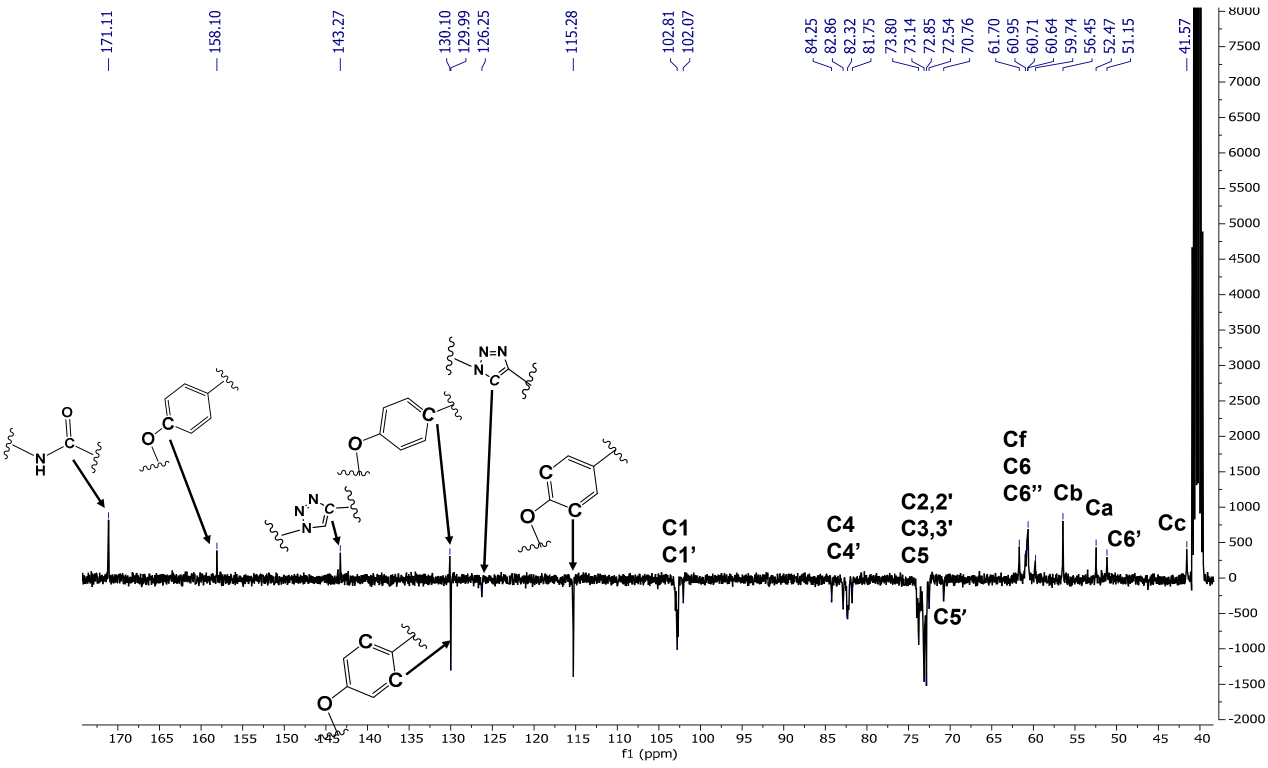

Figure S96. ^{13}C-NMR spectrum of EDTA4BenCD dendrimer (**E**) in DMSO-d_6.

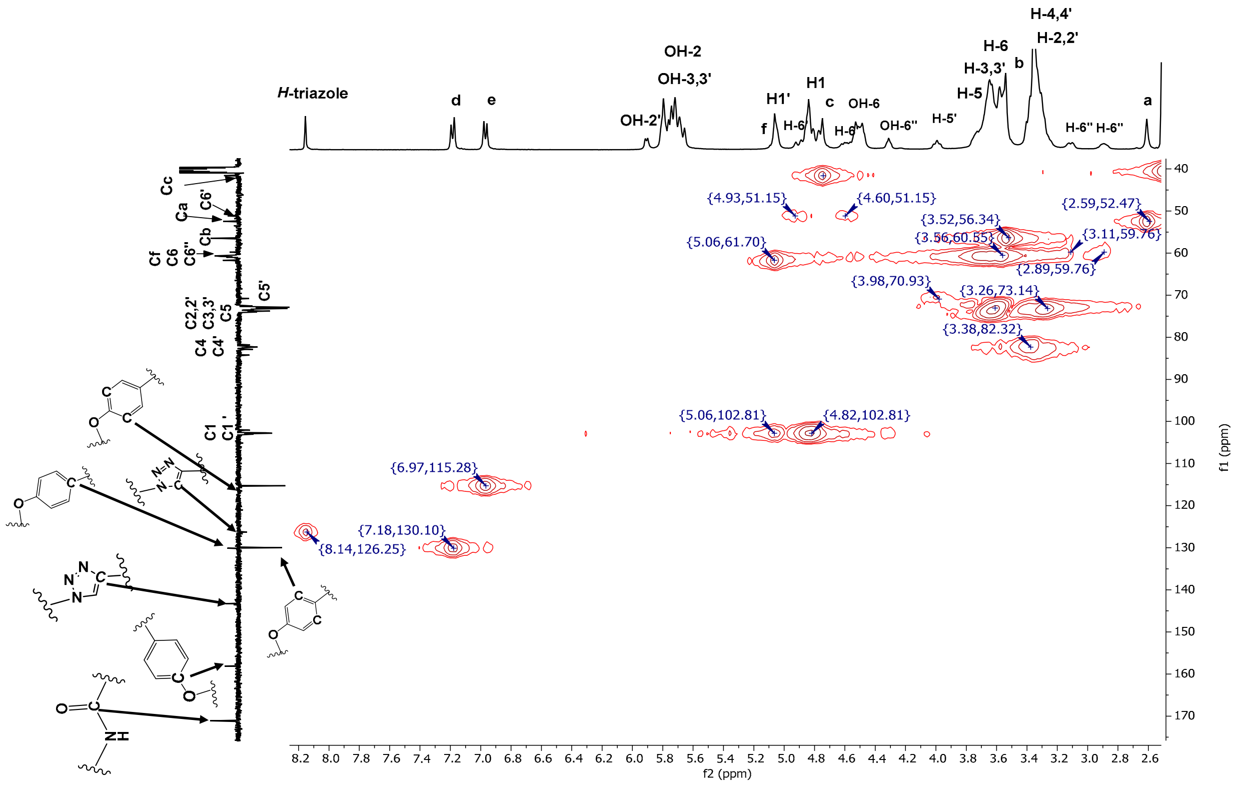

Figure S97. 2D NMR HMQC spectrum of EDTA4BenCD dendrimer (**E**) in DMSO-d_6.

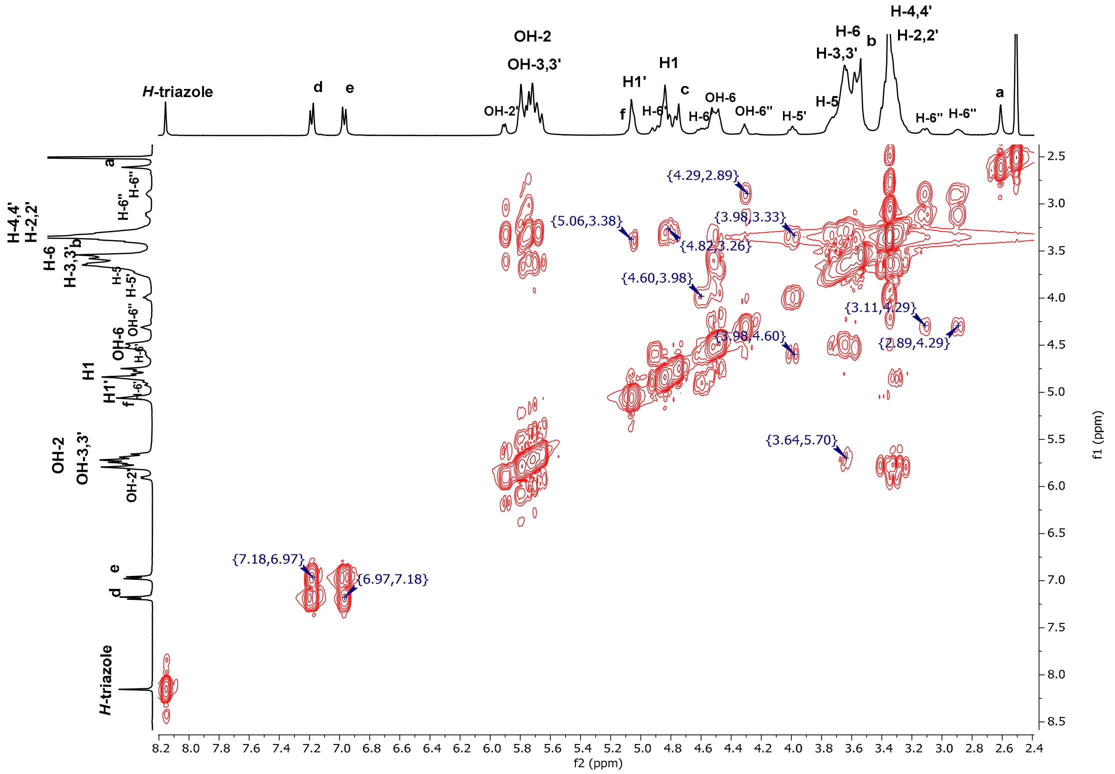

Figure S98. 2D NMR COSY spectrum of EDTA4BenCD dendrimer (**E**) in DMSO-d_6.

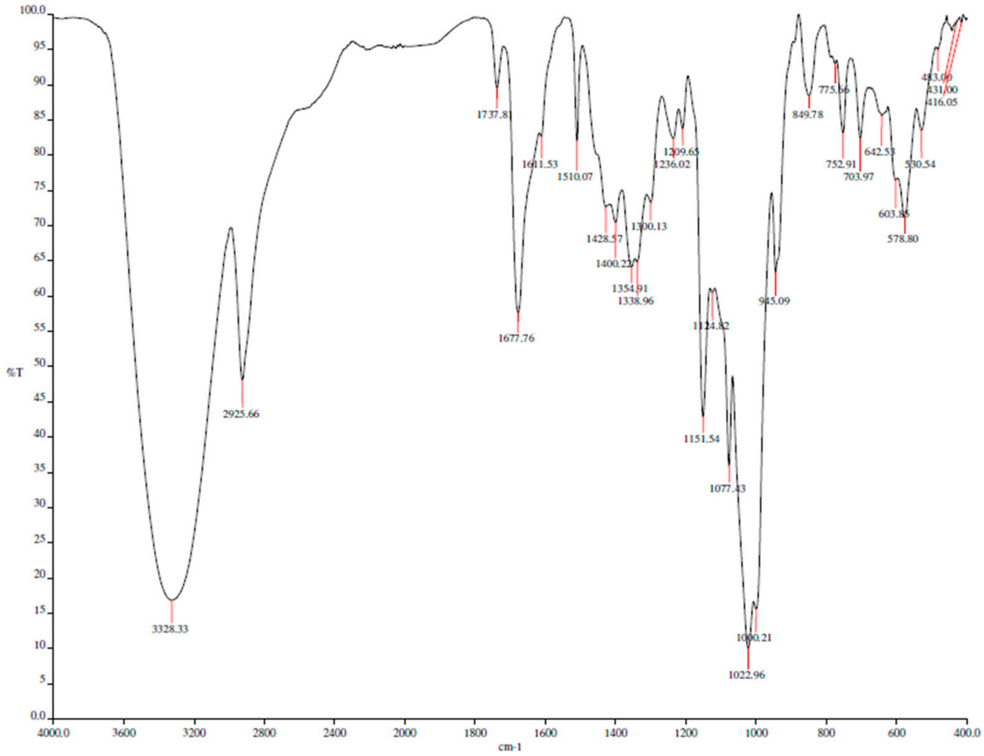

Figure S99. IR spectrum of EDTA4BenCD dendrimer (**E**).

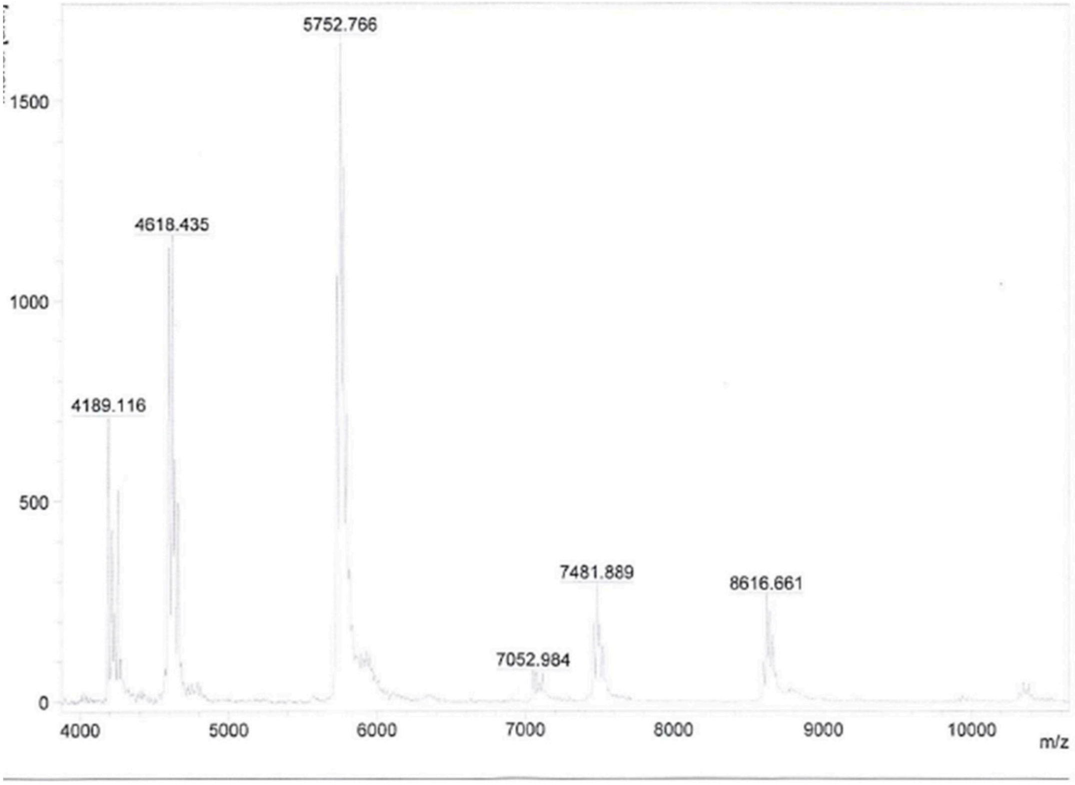

Figure S100. MALDI-TOF spectrum of EDTA4BenCD dendrimer (**E**).

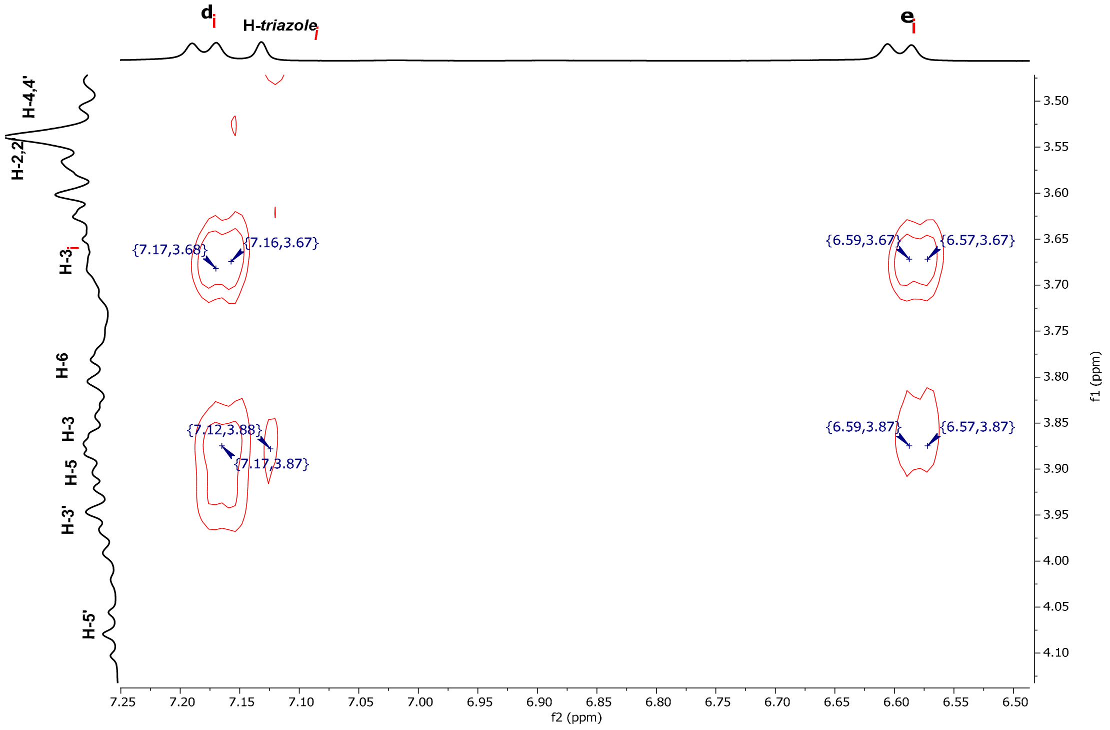

Figure S101. 2D NMR NOESY spectrum of EDTA4BenCD dendrimer (**E**) in D_2O.

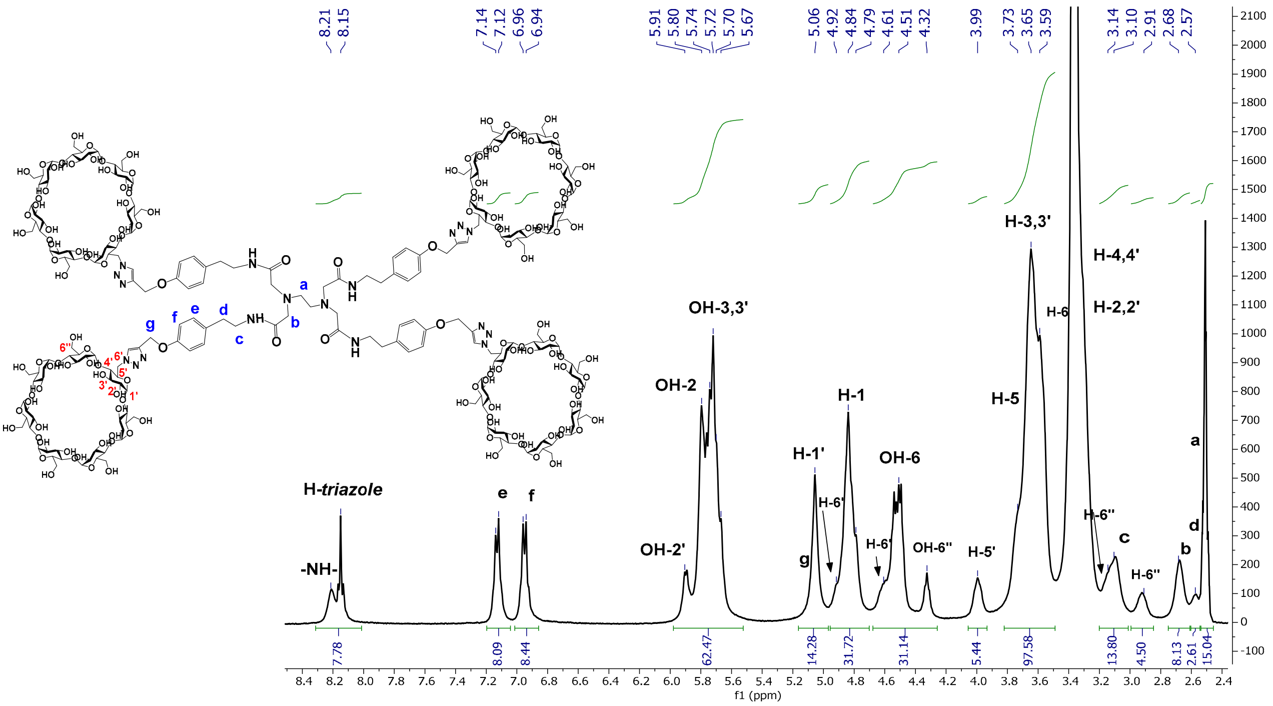

Figure S102. ^{1}H-NMR spectrum of EDTA4TyrCD dendrimer (**F**) in DMSO-d_6.

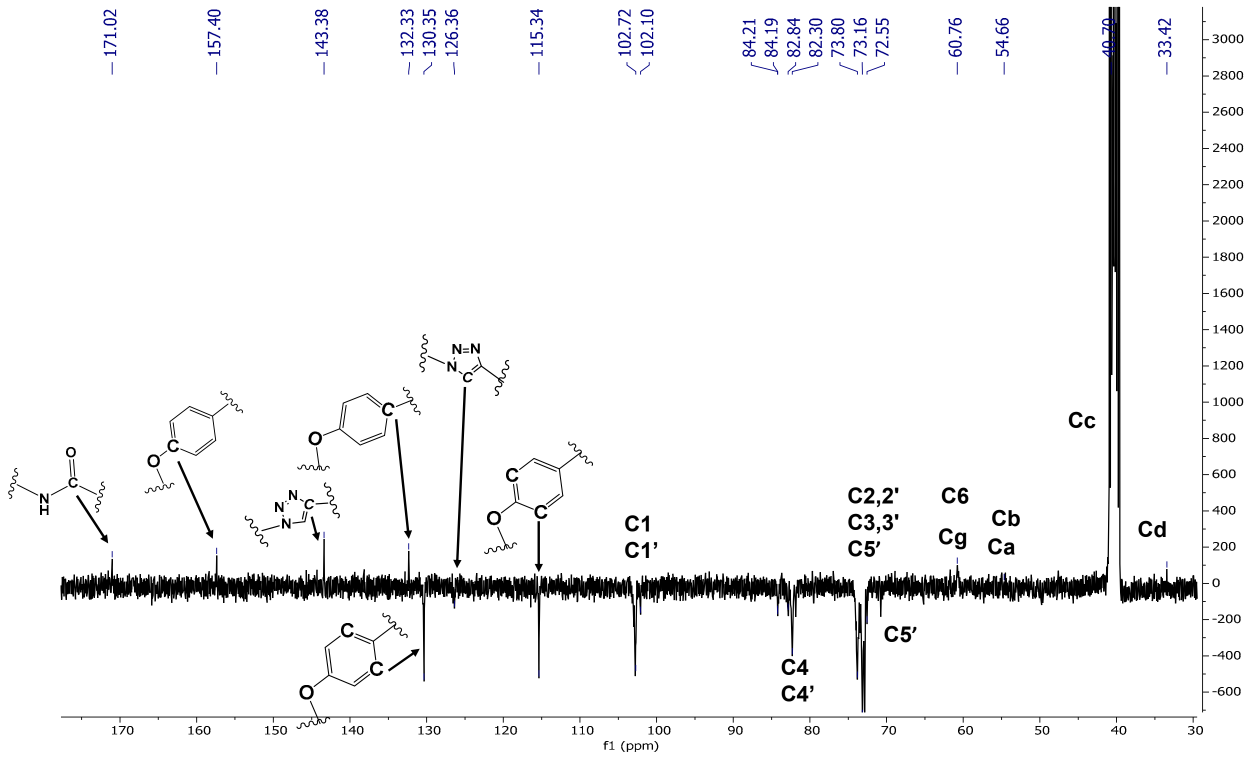

Figure S103. ^{13}C-NMR spectrum of EDTA4TyrCD dendrimer (**F**) in DMSO-*d*$_6$.

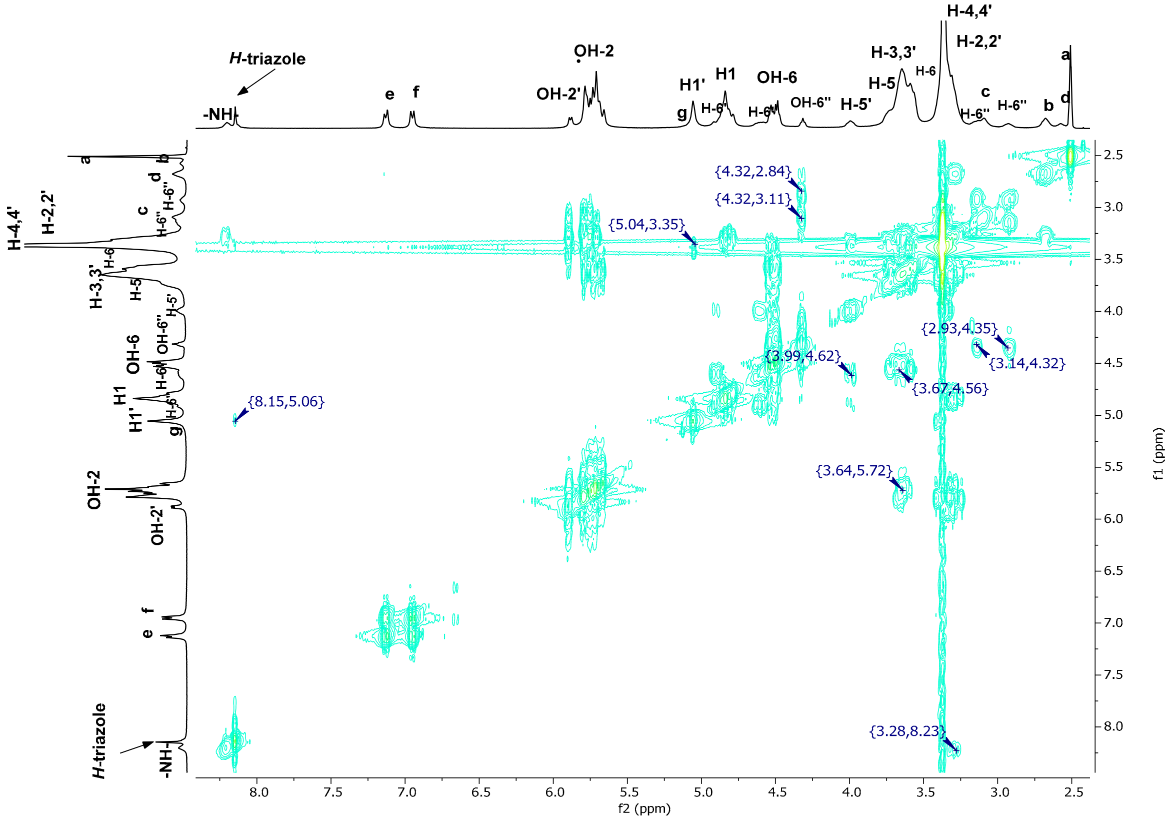

Figure S104. 2D NMR COSY spectrum of EDTA4TyrCD dendrimer (**F**) in DMSO-*d*$_6$.

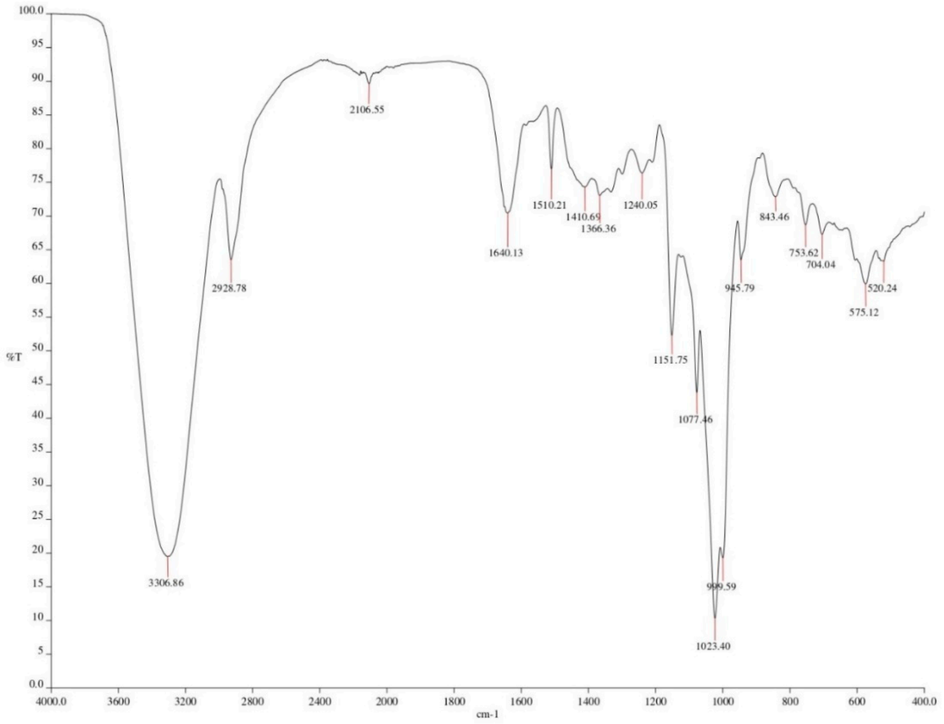

Figure S105. IR spectrum of EDTA4TyrCD dendrimer (**F**).

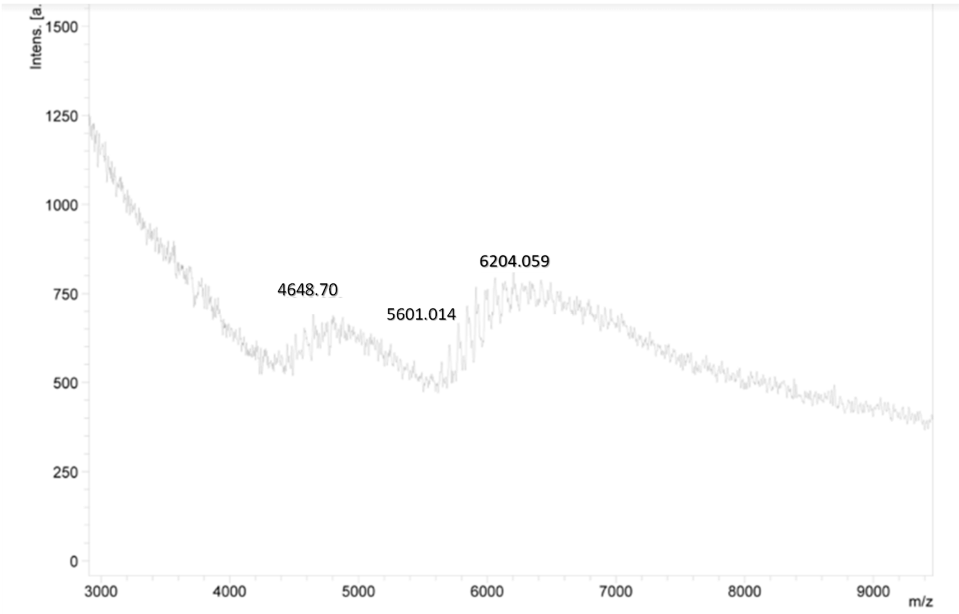

Figure S106. MALDI-TOF spectrum of EDTA4TyrCD dendrimer (**F**).

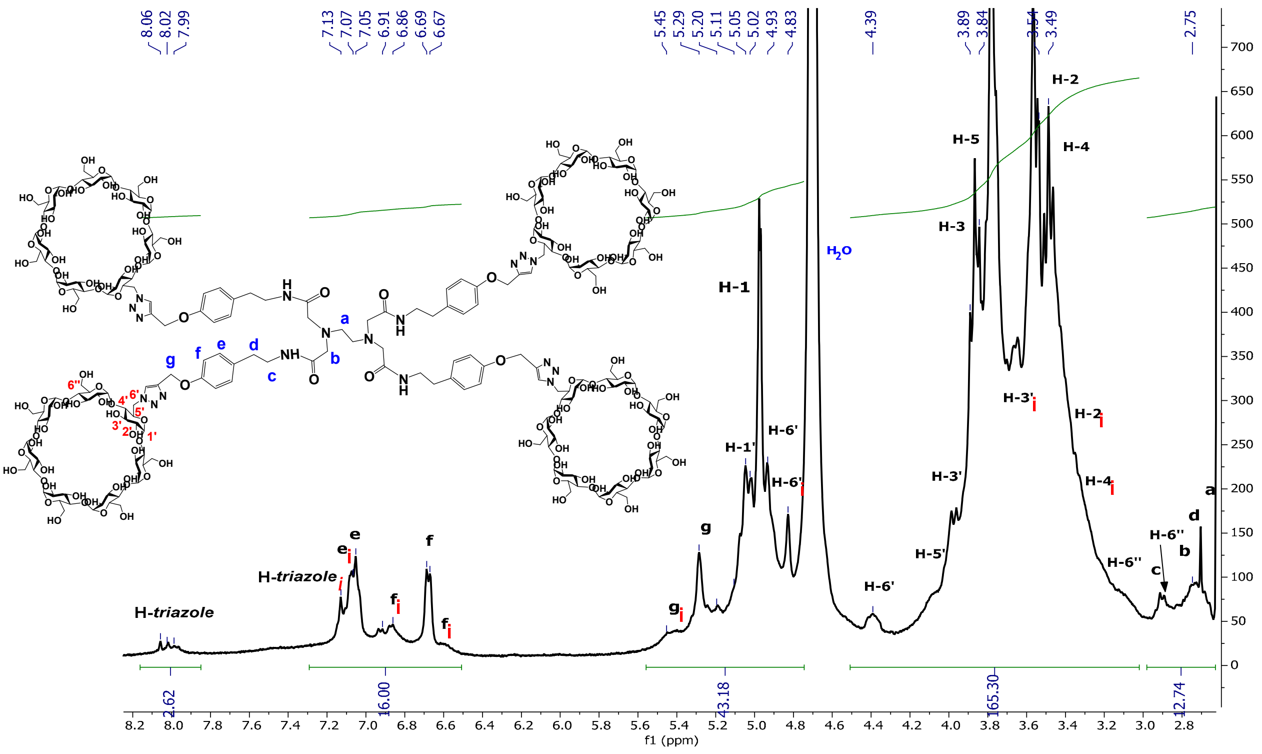

Figure S107. ^{1}H-NMR spectrum of EDTA4TyrCD dendrimer (**F**) in D_2O.

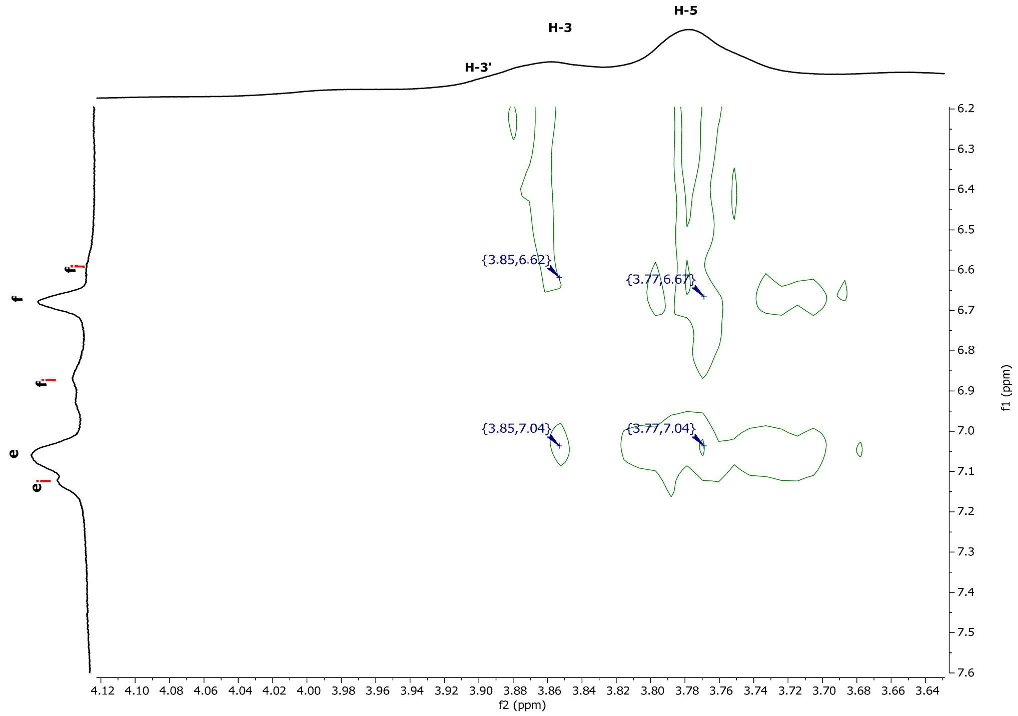

Figure S108. 2D NMR NOESY spectrum of EDTA4TyrCD dendrimer (**F**) in D_2O.

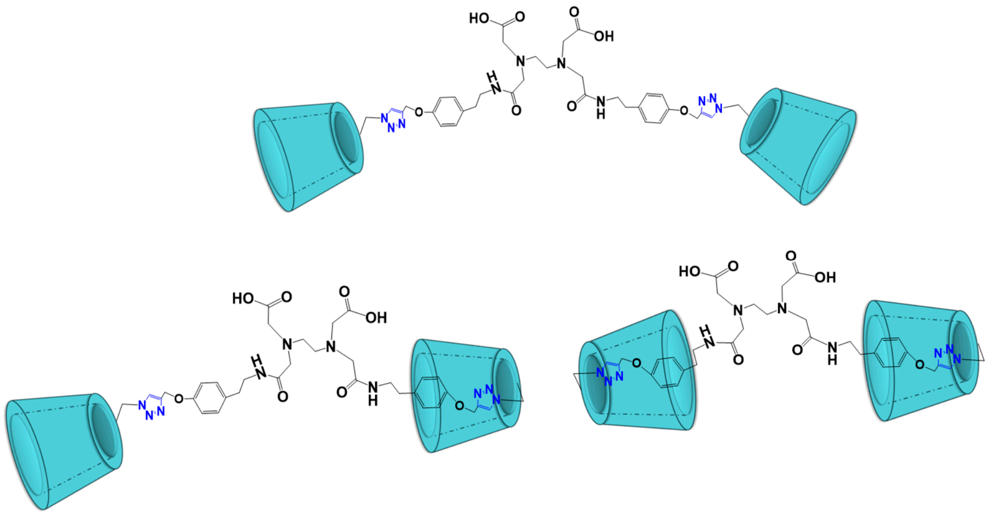

Figure S109. Possible conformations adopted for dimer EDTA2TyrCD (**C**), with 0 to 2 reversed cavities.

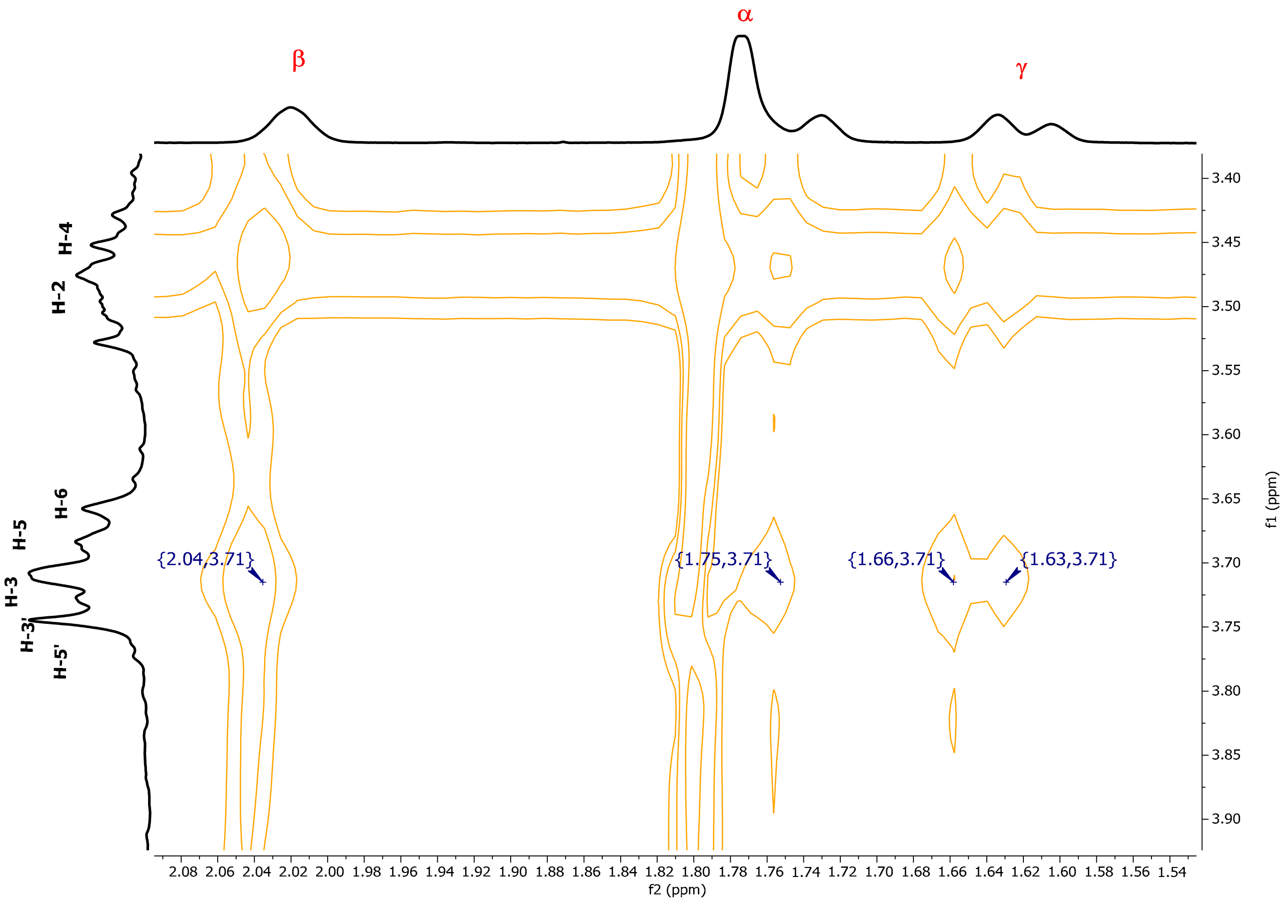

Figure S110. Amplification of aliphatic zones in 2D NMR NOESY spectrum of IC EDTA2TyrCD (**C**) with AdCOOH in D_2O.
